# Comprehensive analysis of dysregulated circular RNAs and construction of a ceRNA network involved in the pathology of Alzheimer’s disease in a 5 × FAD mouse model

**DOI:** 10.3389/fnagi.2022.1020699

**Published:** 2022-11-17

**Authors:** Ting Sun, Li Zeng, Zhongdi Cai, Qingshan Liu, Zhuorong Li, Rui Liu

**Affiliations:** ^1^Institute of Medicinal Biotechnology, Chinese Academy of Medical Sciences and Peking Union Medical College, Beijing, China; ^2^Key Laboratory of Ethnomedicine of Ministry of Education, School of Pharmarcy, Minzu University of China, Beijing, China

**Keywords:** Alzheimer’s disease, circular RNAs, competing endogenous RNA, 5 × FAD mice, microRNAs

## Abstract

**Introduction:**

Alzheimer’s disease (AD) causes a decline in cognitive function that poses a significant hazard to human health. However, the exact pathogenesis of AD and effective treatment have both proven elusive. Circular RNAs (circRNAs), which were initially deemed as meaningless non-coding RNAs, have been shown to participate in a variety of physiological and pathological processes. However, the variations and characteristics of circRNAs are not fairly well understood during the occurrence and development of AD.

**Methods:**

In this study, we performed RNA sequencing analyses, identified circRNA expression profiles, and explored the circRNA-associated competing endogenous RNA (ceRNA) relationship in the hippocampus of five familial AD (5 × FAD) mice with cognitive dysfunction.

**Results:**

The RNA sequencing results identified 34 dysregulated circRNAs in the hippocampus of 5 × FAD mice, including 17 upregulated and 17 downregulated circRNAs. The circRNA-miRNA interaction network for the dysregulated circRNAs was generated, and it was found to include 34 circRNAs and 711 miRNAs. Next, 2067 mRNAs potentially modulated by upregulated circRNA-interacting miRNAs and 2297 mRNAs potentially modulated by downregulated circRNA-interacting miRNAs were identified. Pathway enrichment analyses revealed that the circRNA-miRNA-mRNA network modulated AD development *via* multiple pathways, such as axon guidance, mitogen-activated protein kinase, and neurotrophin. The associated biological processes were mainly related to neuron projection development, cell morphogenesis, and head development. Their corresponding distributions were especially high in the axon, postsynapse, and neuronal body. We constructed a ceRNA network that included five circRNAs, four miRNAs, and 188 mRNAs. In this network, the differential expressions of three circRNAs (circRNA04655, circRNA00723, and circRNA01891), two miRNAs (miR-3470b and miR-6240), and 13 mRNAs (*Vgll3*, *Nhsl2*, *Rab7*, *Tardbp*, *Vps33b*, *Fam107a*, *Tacr1*, *Ankrd40*, *Creb1*, *Snap23*, *Csnk1a1*, *Bmi1*, and *Bfar*) in the hippocampus of 5 × FAD mice using qRT-PCR analyses were consistent with the RNA sequencing results. Another one circRNAs (circRNA00747) and two mRNAs (*Zfp37* and *Polr1e*) had similar expression trends to the sequencing data, while circRNA03723 and *Mapk10* had deviated expression trends to the sequencing data.

**Conclusions:**

In conclusion, our study uncovered dysregulated circRNA expression profiles in the hippocampus of 5 × FAD mice, stretched comprehension of ceRNA biology, investigated the potential role of this ceRNA network in pathogenesis and progression, and identified potential biomarkers and therapeutic targets for AD.

**Graphical abstract:**
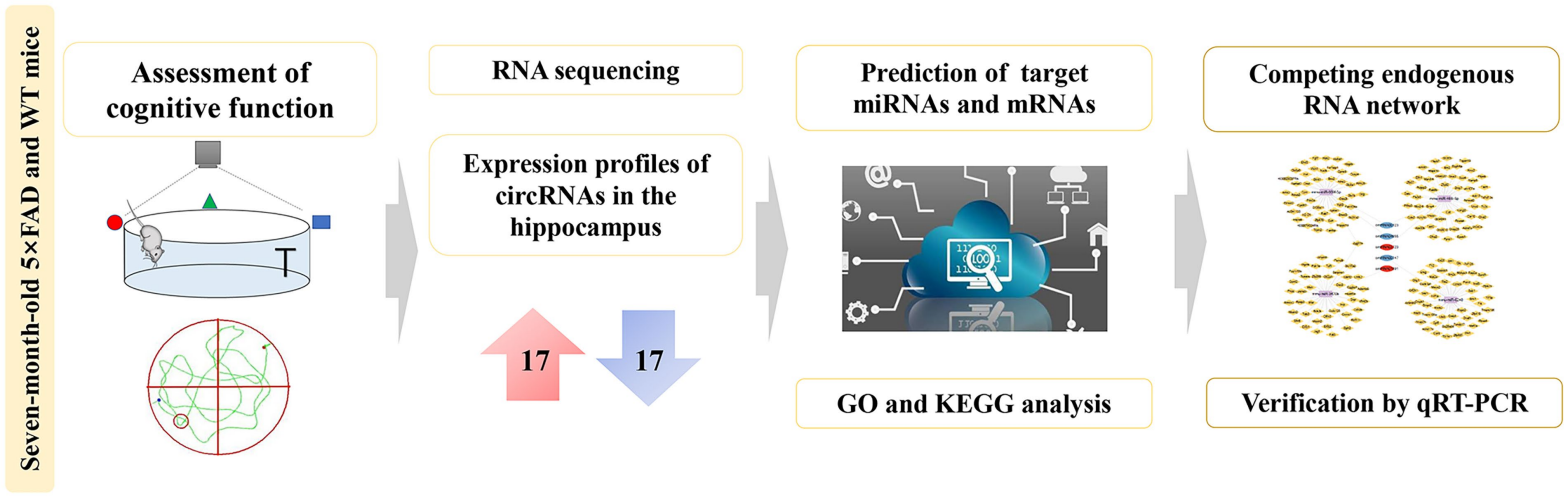
Comprehensive analysis of dysregulated circRNAs and construction of a ceRNA network involved in the pathology of Alzheimer’s disease in a 5 × FAD mouse model.

## Introduction

Alzheimer’s disease (AD) is a critical degenerative disorder that causes progressive impairment of cognition and behavior (Gonzales et al., 2022). The main pathological features of AD are bound up with two protein abnormalities, plaques with excessive accumulation of amyloid and neurofibrillary tangles formed by tau protein hyperphosphorylation. In addition, neuronal loss, neuroinflammation, and synaptic dysfunction can be observed in AD brains (Scheltens et al., 2021). Although there are numerous strategies for slowing or preventing the rapid progression of AD, no feasible treatments are currently available (Mahaman et al., 2021). Therefore, the discovery of possible biomarkers and curative perspectives for AD is of great importance for clinical diagnosis and drug discovery.

Circular RNAs (circRNAs), linked with covalent bonds to form a closed ring, are a particular category of non-coding RNAs (Chen et al., 2021). Unlike linear RNAs, there are neither 5′ caps nor 3′ polyadenylated tails in circRNAs (Zhang L. et al., 2021). The structure types of circRNAs encompass single exons, multiple exons, introns, and combinations of introns and exons (Chen, 2020). Because their loop structure is fixed by covalent bonds, circRNAs are resistant to exonucleases and highly stable (Sanger et al., 1976). circRNAs were originally considered an artifact of alternative splicing caused by exon transcription errors and did not serve any physiological function (Hansen et al., 2013). However, evidence is emerging that circRNAs are implicated in the occurrence and development of multiple diseases, including nervous system disorders, cardiovascular diseases, cancer, and diabetes. Moreover, circRNAs are thought to play different functional roles depending on their location (Yang et al., 2017). Thus, circRNAs in the nucleus mainly transcribe their parental genes (Zhou et al., 2020), while those in the cytoplasm are mainly miRNA sponges, protein sponges, or templates for peptides translation (Piwecka et al., 2017).

circRNAs are abundantly distributed in the mammalian nervous system (compared with other organs), where they are involved in neuronal differentiation and synaptogenesis (Gokool et al., 2020). Research in *Drosophila* and mouse has revealed that circRNA expression is dramatically increased (relative to linear RNAs) in the central nervous system with aging (Westholm et al., 2014; Gruner et al., 2016). As a consequence, circRNAs may be indispensable signals in neurodegenerative diseases. In line with the broad spectrum and accuracy of RNA sequencing technologies, circRNAs functions and changes in their expression profile during AD progression have attracted increasing attention. In AD mice, circRNAs may function as miRNA sponges *via* the competing endogenous RNA (ceRNA) action. A study revealed that the serum of AD patients contains abnormally high levels of circLPAR1, which increases target gene levels by competitively binding to miR-212-3p (Wu et al., 2021). Likewise, ciRS-7 functions as a ceRNA to sponge miR-7 in the regulation of ubiquitin-protein ligase A, which is well-known to coordinate the scavenging of amyloid (Zhao et al., 2016). Currently, the role of circRNAs in the pathogenesis of AD and the complexity involved in the circRNA-centered ceRNA networks have not been widely studied.

**Figure 1:**
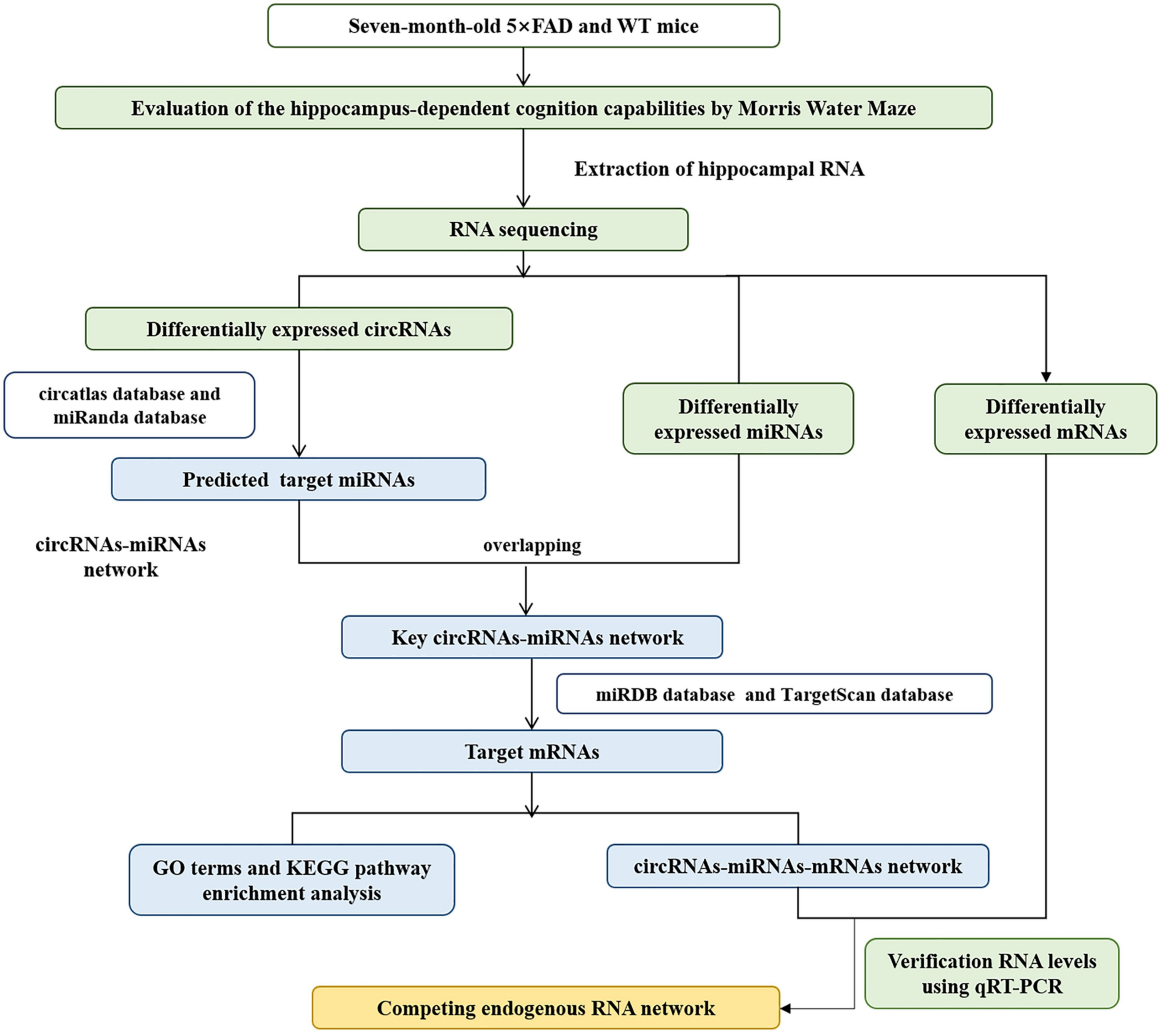
Workflow for circRNA analysis and ceRNA network construction.

In this study, we firstly reported the differentially expressed circRNA profiles in the hippocampus of five familial AD (5 × FAD) mice compared with wild-type (WT) mice using RNA sequencing analysis. Gene Ontology (GO) and Kyoto Encyclopedia of Genes and Genomes (KEGG) enrichment analysis presented the potential biological functions and signaling pathways of these dysregulated circRNAs. Furthermore, we established a circRNA-miRNA-mRNA network from the present sequencing data to obtain new potential biomarkers and drug targets for AD. Finally, we verified the expression of key circRNAs, miRNAs, and mRNAs within the network using qRT-PCR analysis (Figure 1). These findings might provide a better understanding of the characteristics and functions of circRNAs in the pathogenesis of AD.

## Materials and methods

### Animals

Seven-month-old 5 × FAD mice and age-matched WT mice, with one male and two females in each group, were used in this study. The 5 × FAD mice are typical transgenic mice for AD research that co-express a total of five familial AD mutations [*APP K670N/M671L* (Swedish) + *I716V* (Florida) + *V717I* (London) and *PS1 M146L* + *L286V*]. The Experimental Animal Care and Use Committee of Institute of Medicinal Biotechnology approved the experimental animal protocols (No. IMB-20210602D101). All mice were housed in an environment with a standard diet and clean water.

### Cognitive behavior test

The Morris water maze (MWM) test was used to evaluate the impairment of spatial learning and memory of 5 × FAD mice (Liu et al., 2018; Jiang et al., 2022; Sun et al., 2022). A different cohort from the Cai et al. (2022) study was used for MWM test. Briefly, The testing included place navigation training and space memory trials. During the first 5 days, place navigation training of mice was conducted. Each mouse performed four tests from four quadrants as starting position, and each investigation was limited to 60 s. The time that mice found the platform was recorded as escape latency. Those mice that did not find the platform within 60 s were directed to the platform and stayed for 60 s. On the 6th and 7th days, spatial memory trials were performed in the pool without the platform. The number of crossings (as mice sought the original platform) and the duration within the target quadrant (where the original platform was located) were recorded.

### RNA extraction

After the MWM, the mice were euthanized *via* cervical dislocation, and the obtained samples were preserved in liquid nitrogen. The total RNA of the hippocampus was isolated with TRIzol reagent (Invitrogen, CA, United States) according to the previous protocol (Zhao et al., 2022). The RNA sample used in this study was the same cohort as our reported investigation (Cai et al., 2022). Subsequently, the RNA integrity was determined using gel electrophoresis (Supplementary Figure 1). The RNA concentration was determined on Spark 20 M microplate reader (Tecan Group Ltd., Switzerland; Supplementary Table 1).

### RNA sequencing

Ribosomal and linear RNAs were removed from total RNA using the Ribo-off rRNA Depletion kit and RNase R (Vazyme Biotech Co., Ltd., China). The rRNA free residue was cleaned up by ethanol precipitation. cDNA libraries were constructed from rRNA-depleted RNA using the VAHTS^™^ Stranded mRNA-seq Library Prep Kit for Illumina (Vazyme Biotech Co., Ltd., China). The quality of the libraries was then estimated using Agilent 2100 Bioanalyzer system and FastQC (Version 0.11.2). Mass shear was performed by Trimmomatic (Version 0.36) to obtain relatively accurate and effective data. An Illumina HiSeq 2500 platform was used to analyze the library sequences. CircRNAs were identified using CIRI2 (Version 2.06) after alignment with the reference genome using Burrows-Wheeler-Alignment Tool. Subsequently, variable shear analysis was performed using CIRI-AS (Version 1.2). BEDtools was used to determine the origin of circRNAs based on circRNA location information and gene annotations. The Reads Per Kilobase Million (RPKM) formula based on BSJ reads was used to calculate the expression of circRNA and DESeq2 (Version 1.12.4) was used for differential analysis of transcript expression. The RNA sequencing procedures were undertaken by Sangon Biotech (Shanghai, China). Data have been deposited in Gene Expression Omnibus (GEO) with the accession code GSE206562.

### Prediction of miRNA targets of circRNAs and ceRNA network construction

The circatlas^1^ and miRanda^2^ were used to predict the miRNAs with the strongest binding to differently expressed circRNAs. The circatlas is a comprehensive resource database that integrated over one million circRNAs across multiple species and a variety of tissues, as well as the binding relationships between circRNAs and miRNAs. The miRanda database provided predictive information about miRNA targets in the genomes of multiple species and the expression profiles of miRNAs in different tissues. miRDB,^3^ and TargetScan^4^ databases were employed to predict the interactions of mRNA-miRNA. miRDB is a database for miRNA target prediction and functional annotations. TargetScan predicted biological targets of miRNAs by searching for the presence of conserved sites that match the seed region of each miRNA. A circRNA-miRNA-mRNA network was then formed and visualized using Cytoscape software (v3.7.2).

### Enrichment analyses of GO enrichment and KEGG enrichment

KEGG enrichment was performed to analyze the enriched pathways associated with circRNA target genes (Chen et al., 2020; Shen et al., 2020). The GO knowledge base was used to describe information on the functions of genes, including biological processes (BP), molecular functions (MF), and cellular components (CC). GO and KEGG analyses were implemented using GENEONTOLOGY^5^ and KEGG^6^ databases.

### Detection of RNA expression levels by qRT-PCR

The remaining RNA samples from the RNA sequencing were used to perform qRT-PCR. For circRNA, mRNA and GAPDH, cDNA was synthesized by HiscriptIII RT SuperMix 1st Strand cDNA synthesis kit and the qRT-PCR was completed using SYBR Master Mix kits (Vazyme, Nanjing, China). For miRNA and U6, miRNA Synthesis Kit and miRNA SYBR Master Mix were used (Vazyme, Nanjing, China). Sangon Biotech (Shanghai, China) produced all primers (Table 1). Relative gene expression levels were calculated using the 2^−ΔΔCT^method.

**Table 1:** Primer sequences of the RNAs in the present study.

Name	Sequence (5′ → 3′)
miR-3470b-F	AGGTGTTCTCACTCTGTAGACC
miR-3470b-R	CAGTGCAGGGTCCGAGGT
miR-3470b-RT	GTCGTATCCAGTGCAGGGTCCGAGGTATTCGCACTGGATACGAC
miR-6240-F	AAGTATTCCCAAAGCATCGCG
miR-6240-R	CAGTGCAGGGTCCGAGGT
miR-6240-RT	GTCGTATCCAGTGCAGGGTCCGAGGTATTCGCACTGGATACGAC
miR-466i-5p-F	ACGAGATCTGTGTGTGTGTGT
miR-466i-5p-R	CAGTGCAGGGTCCGAGGT
miR-466i-5p-RT	GTCGTATCCAGTGCAGGGTCCGAGGTATTCGCACTGGATACG
miR-669f-5p-F	AATGCACAGTTGTGTGTGCAT
miR-669f-5p-R	CAGTGCAGGGTCCGAGGT
miR-669f-5p-RT	GTCGTATCCAGTGCAGGGTCCGAGGTATTCGCACTGGATACG
circRNA01891-F	CGCTGCTCAGGAATGGAAGTT
circRNA01891-R	ACAAACCACATGAGCCCAACC
circRNA00747-F	TCAACACTGGTAGTGCGGTAA
circRNA00747-R	ATTCTCTTGACCTTTCCTTTCTCCT
circRNA03723-F	TCCTGCTCACCTGCACTACA
circRNA03723-R	TCCAGCGGAACCACAGTTTC
circRNA00723-F	AGCATGGTGAGTAATGCCTTGA
circRNA00723-R	ATGAGAGAGGCAGCTGACAGTA
circRNA04655-F	TGACCCCAAGGATGCAGAGA
circRNA04655-R	GCAGAGAAGCCACATCACACA
Vgll3-F	GCGTCTGAGTCTTGGCACTATCC
Vgll3-R	TGAGGTAGCCGTGGTGACTGTAG
Nhsl2-F	AGGAACCTGGAGCAGAGGAAGTC
Nhsl2-R	TGAGAGGACAAGGCTGGAGGATG
Rab7-F	AGGAGGTGATGGTGGACGACAG
Rab7-R	GGCACTGGTCTCGAAGTAAGGAATG
Tardbp-F	GATAGATGGGCGATGGTGTGACTG
Tardbp-R	GCAAAGGTGACGAAGGCAAAAGC
Zfp37-F	CACACTGCCGTACTCCATCTAAACC
Zfp37-R	TGCCACACTGTTCACACTCGTATG
Vps33b-F	TGGAGGAAACTGGAGGAGGAAGAAG
Vps33b-R	TTGCGGATCTCACTGAACACCTTG
Mapk10-F	GTTAGTGATTGACCCAGCGAAGAGG
Mapk10-R	GACAGACGAGGATGGAGGGAGAC
Fam107a-F	CAAAAGGGGCTTGGGTATGGACAG
Fam107a-R	GTCAGTGTGGTGATTCTGCGTAGG
Tacr1-F	AACTTCACCTACGCAGTCCACAAC
Tacr1-R	GCATAGCCAATCACCAGCAGAGG
Ankrd40-F	AACCGCTCCCTGTTCTCTGTCC
Ankrd40-R	CAGCAGTTCTTGGTAGGTGAGTTCC
Creb1-F	GCTGGCTAACAATGGTACGGATGG
Creb1-R	GCTGCCTCCCTGTTCTTCATTAGAC
Snap23-F	GCCTCTGCATCTGCCCTTGTAATAG
Snap23-R	TATCCACCACTGGCTGCTCCTG
Csnk1a1-F	ACAACAGGACAAGGCAACACATACC
Csnk1a1-R	CAACACCTCAACAGGAGTGGACATC
Bfar-F	GGAGTGAAGCCACCGCAGAATC
Bfar-R	AACAGAGAGCAGCACGGCATTG
Polr1e-F	CCTGCTGGATGACGATGCTATTGAG
Polr1e-R	GGAGGAAGGTAGAGGACGCCATC
Bmi1-F	GATGGACTGACGAATGCTGGAGAG
Bmi1-R	TGGCAAAGGAAGATTGGTGGTTAGC
GAPDH-F	TGACTTCAACAGCGACACCCA
GAPDH-R	CACCCTGTTGCTGTAGCCAAA

### Acquisition of circRNA datasets and data processing

To clarify the novelty of these key circRNAs in the hippocampus of 5 × FAD mice, we compared the circRNAs from different datasets in various AD models that were downloaded from the GEO, with the species limitation (mouse and rat) and the biological sample type (different brain regions). The circatlas^7^ and circBase^8^ were used to convert the circRNA ID and name. The GEO dataset containing the expression level of cicrRNA and miRNA associated with AD were selected, including GSE138382, GSE129053, GSE121769, GSE52023, GSE166393, GSE48028, GSE158995, GSE190880, GSE132177, GSE194137, GSE153180, GSE20447, GSE186929, and GSE129054. The significantly dysregulated RNAs were screened with|Fold Change| ≥ 1 and *p-*value <0.05.

### Statistical analysis

Data analyses were conducted by SPSS software (Version 18.0, SPSS, Inc., Chicago, IL, United States). One-way Repeated Measures ANOVA was used to analyze escape latency and swimming speed. Student’s *t*-test was used to analyze the other data. Differentially expressed circRNAs, miRNAs, and mRNA following the RNA sequencing were screened with |Fold Change| ≥ 1 and *p-*value <0.05. Results are presented as mean ± standard deviation (S.D.). The statistical criterion used was *p* < 0.05.

## Results

### Spatial cognition dysfunction in 5 × FAD mice

**Figure 2:**
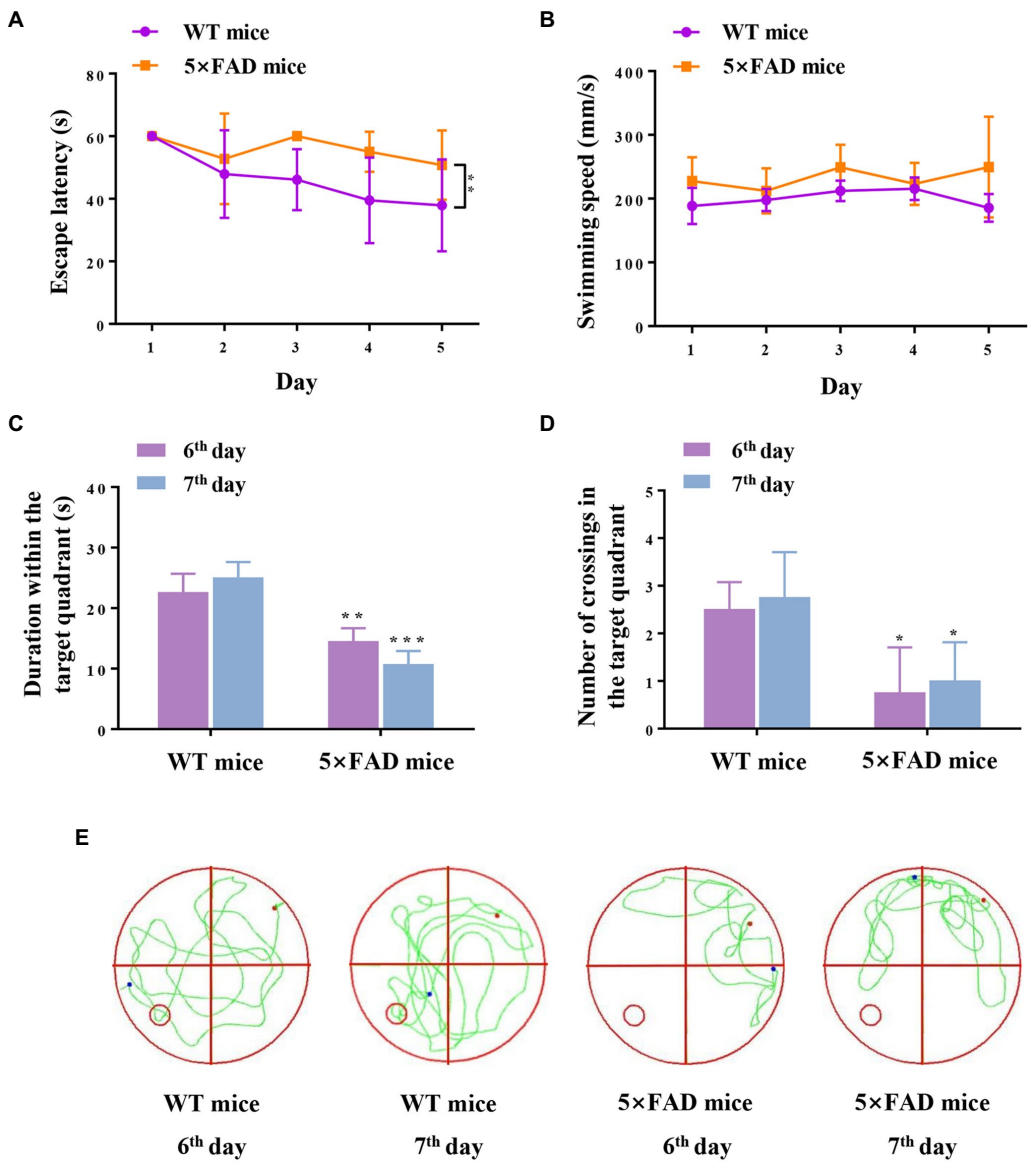
Cognition and memory capabilities in WT and 5 × FAD mice. **(A)** Escape latency of mice during five-day navigation training in MWM test. **(B)** Swimming speed during five-day navigation training in MWM test. **(C)** Duration within the target quadrant in space memory trials on the 6th and 7th days. **(D)** Number of crossings through the original platform position in space memory trials on the 6th and 7th days. **(E)** Representative traces of mice in the spatial probe test on the 6th and 7th days. The red dots indicate the position of the mouse at the beginning of the spatial exploration trial, the blue dots indicate the position of the mouse at the end of the 60-s trial, and the green line indicates the representative traces of mice. Data are displayed as mean ± SD (*n* = 3). *p < 0.05, **p < 0.01, ***p < 0.001 vs. WT mice.

We estimated the hippocampus-dependent cognition capabilities of 5 × FAD mice using the MWM test prior to RNA-sequencing detection. The results revealed that 5 × FAD mice exhibited impairments in recognition ability (compared with WT mice), as demonstrated by longer escape latencies (*p* = 0.0072; Figures 2A), less duration within the target quadrant (*p* = 0.0052 and *p* = 0.0001, respectively; Figures 2C), as well as fewer platform crossings (*p* = 0.02 and *p* = 0.03, respectively; Figures 2D). 5 × FAD mice did not exhibit motor dysfunction, reflected in the same swimming speeds as WT mice (Figure 2B). Representative traces of the spatial memory trials are shown in Figure 2E, and these indicate that 5 × FAD mice exhibited an aimless travel path. Collectively, the above results reveal that 7-month-old 5 × FAD mice present hippocampus-dependent spatial cognitive impairment.

### Identification of circRNAs in 5 × FAD mice

**Figure 3:**
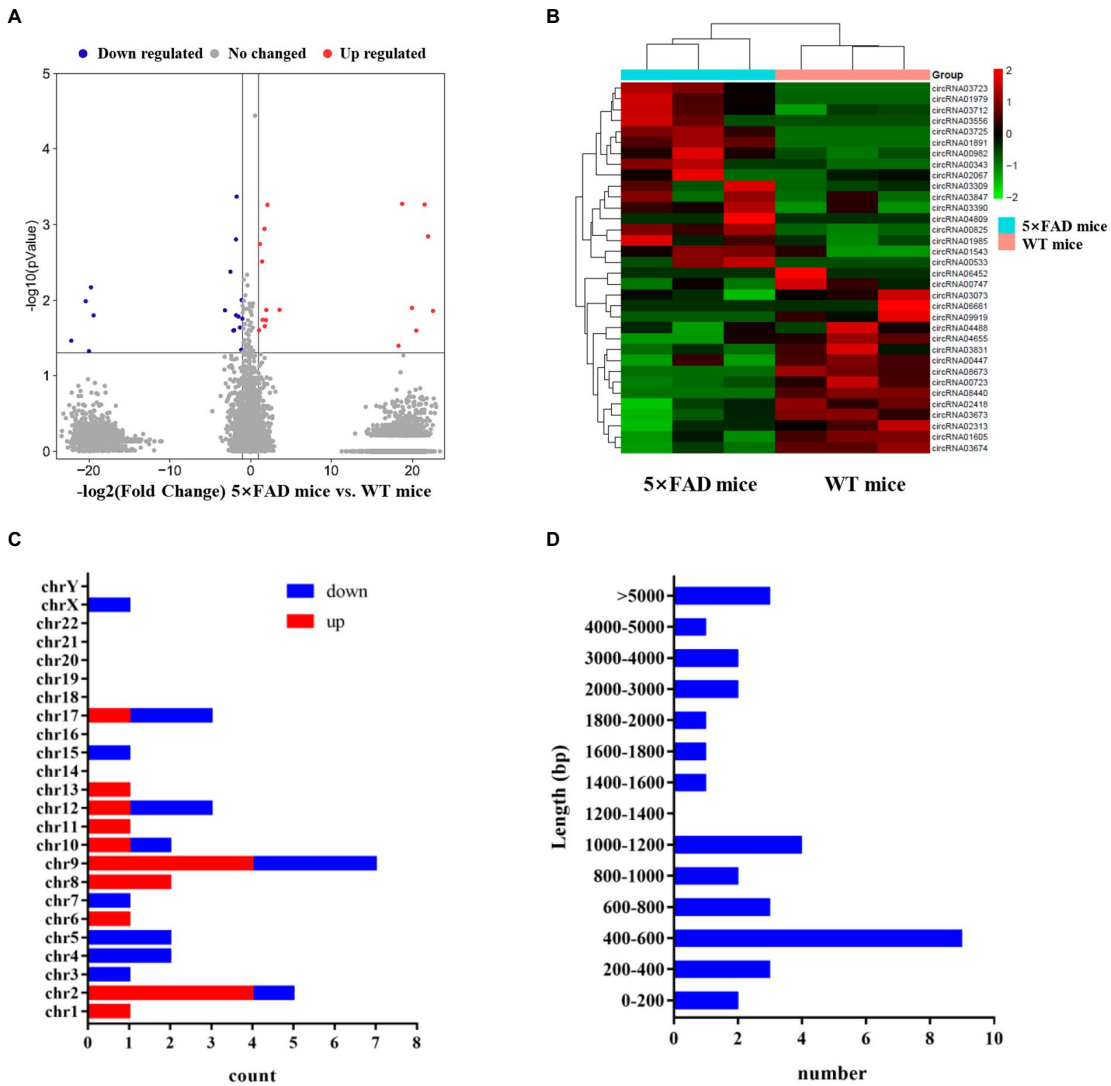
Expression profiles of circRNAs in 5 × FAD mice and WT mice. **(A)** Volcano plot of the circRNAs in the hippocampus of 5 × FAD mice and WT mice. The red dots indicate significantly upregulated circRNAs, the blue dots indicate the significantly downregulated circRNAs, and the gray dots indicate the circRNAs that have no change. **(B)** Heatmap of the aberrant circRNAs indicating the variation of significantly dysregulated circRNAs in the hippocampus of 5 × FAD mice. **(C)** Chromosomal locations of the aberrant circRNAs. The *X*-axis shows the number of circRNAs, and the *Y*-axis represents the chromosomal number. **(D)** The sequence length of the aberrant circRNAs. The *X*-axis indicates the number of circRNAs, and the *Y*-axis represents the length ranges.

The next-generation sequencing was performed to profile circRNA expression in the hippocampus of 5 × FAD and WT mice. In total, 11,112 circRNAs were detected in the hippocampus through sequencing. The expression distribution of all circRNAs was presented in the volcano diagram, in which the expression of 34 circRNAs was significantly different between 5 × FAD and WT mice, including 17 upregulated and 17 downregulated circRNAs (Figure 3A). The details of these circRNAs are listed in Table 2. A clear distinction between the circRNA expression patterns in different species of mice was shown in the hierarchical clustering diagram (Figure 3B). In addition, we classified the aberrantly expressed circRNAs in the light of their chromosomal location (Figure 3C). The results reveal that 20.58 and 14.7% of circRNAs were derived from chromosome 9 and 2, respectively. Finally, we found that circRNAs with lengths between 400 and 600 bp accounted for the major proportion (Figure 3D).

**Table 2:** Information of differentially expressed circRNAs.

circRNAs	log2Fold change	*P*-value	Regulation	GC content (%)	circRNAs type
circRNA03556	22.53377519	0.013892798	Up	45.40229885	Exonic
circRNA03725	21.92096778	0.001434715	Up	40.90909091	Exonic
circRNA01979	21.48389791	0.000544358	Up	47.88069074	Exonic
circRNA00533	20.45822601	0.025267743	Up	43.75	Exonic
circRNA01891	19.93244316	0.012664519	Up	44.94382022	Exonic
circRNA03723	18.71480295	0.000533453	Up	39.52113509	Exonic
circRNA04809	18.26752047	0.040125467	Up	60.10781671	Exonic
circRNA00343	3.559259969	0.013435605	Up	44.24951267	Exonic
circRNA03712	2.066163958	0.000549785	Up	41.26106195	Exonic
circRNA01543	1.931690653	0.013484152	Up	68.37606838	Exonic
circRNA03390	1.881637631	0.018354902	Up	52.32903865	Exonic
circRNA03847	1.734807686	0.022120789	Up	45.40441176	Exonic
circRNA00982	1.695434754	0.001141365	Up	34.39878234	Exonic
circRNA02067	1.469244466	0.018185027	Up	51.09649123	Exonic
circRNA01985	1.402530297	0.003080891	Up	51.76056338	Exonic
circRNA00825	1.140773707	0.001808919	Up	44.38239893	Exonic
circRNA03309	1.030662355	0.025049036	Up	52.29681979	Exonic
circRNA02418	−1.033076974	0.017550638	Down	42.22440945	Exonic
circRNA04488	−1.133775465	0.009987767	Down	49.91776316	Exonic
circRNA01605	−1.177668696	0.045440296	Down	55.20446097	Exonic
circRNA03073	−1.325214463	0.023002072	Down	41.03819785	Exonic
circRNA02313	−1.512189587	0.016491331	Down	48.03293687	Exonic
circRNA00723	−1.734546089	0.000427638	Down	46.40522876	Exonic
circRNA00447	−1.803989196	0.015843509	Down	41.18476728	Exonic
circRNA03673	−1.817298272	0.001569667	Down	50.70063694	Exonic
circRNA04655	−2.051676194	0.025000319	Down	46.46924829	Exonic
circRNA00747	−2.153043019	0.0251707	Down	38.07649044	Exonic
circRNA03674	−2.508877673	0.004209075	Down	49.73758579	Exonic
circRNA03831	−3.183369042	0.013633279	Down	41.19496855	Exonic
circRNA06452	−19.42529847	0.015893812	Down	46.24277457	Exonic
circRNA08673	−19.74008741	0.006766295	Down	50.51282051	Exonic
circRNA06661	−19.98418488	0.047256341	Down	38.2183908	Exonic
circRNA08440	−20.39221528	0.010326992	Down	52.07995678	Exonic
circRNA09919	−22.18106979	0.034379652	Down	46.51685393	Exonic

### Prediction of circRNA-miRNA interactions

**Figure 4:**
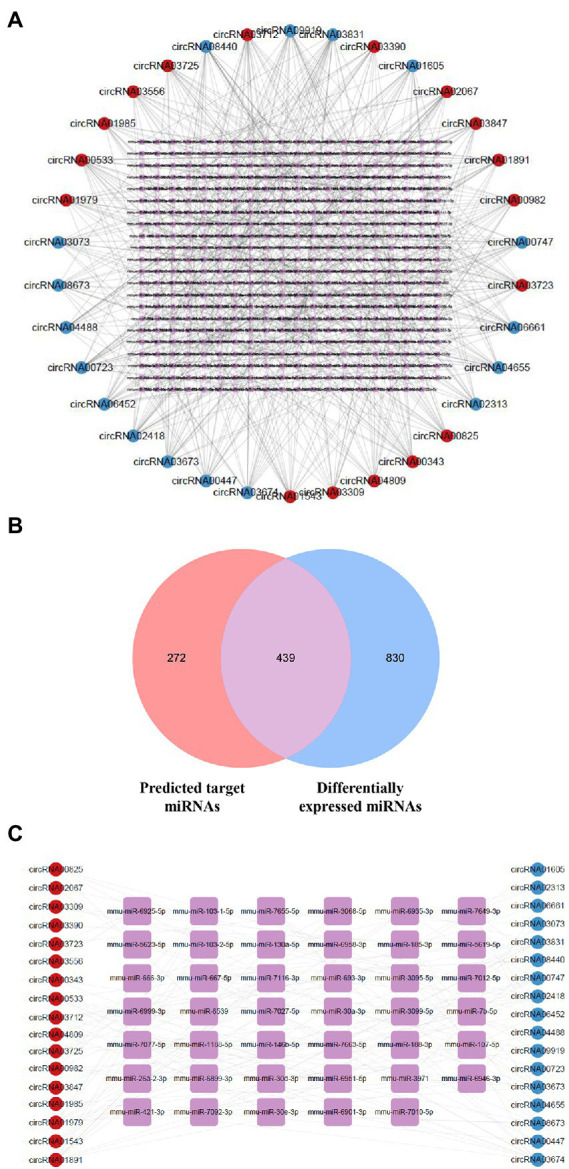
Network of dysregulated circRNAs and target miRNAs. **(A)** Network of dysregulated circRNAs and their predicted miRNAs using the database. Upregulated circRNAs are shown using red circles, downregulated circRNAs using blue circles, and potential miRNAs using purple squares. **(B)** Venn diagram of predicted target miRNAs and abnormally expressed miRNAs in 5 × FAD mice. The number of predicted miRNAs is shown in red, and the number of differentially expressed miRNAs is shown in blue. **(C)** Network of dysregulated circRNAs and key miRNAs selected by the Degree algorithm. Upregulated circRNAs are shown using red circles, downregulated circRNAs are shown using blue circles, and key miRNAs are shown using purple squares.

circRNAs regulate gene expression *via* functioning as sponges that sequester specific target miRNAs (Kristensen et al., 2019). We utilized the circatlas database and the miRanda database to identify miRNAs that potentially correspond to the circRNAs of interest. There were 1,020 circRNA-miRNA interactions involving 34 circRNAs and 711 miRNAs (Figure 4A; Supplementary Table 2). For a more accurate prediction of potential miRNA targets, differentially expressed miRNAs identified in the RNA sequencing experiment were used for further analysis (Supplementary Table 3; Supplementary Figure 2). By intersecting the predicted target miRNAs with the miRNAs identified through sequencing, we identified 439 overlapping miRNAs in the hippocampus of 5 × FAD mice (Figure 4B; Supplementary Table 4). In the resulting network constructed using Cytoscape, we set the algorithm of the Degree to greater than three times the median to obtain 41 key miRNAs. Several types of interactions could be identified within this group of key miRNAs, including four upregulated circRNAs (circRNA03725, circRNA02067, circRNA03847, and circRNA03390) that interacted with downregulated miRNAs, one upregulated circRNA (circRNA03556) that interacted with upregulated miRNAs, three downregulated circRNAs (circRNA03073, circRNA02313, and circRNA04655) that interacted with upregulated miRNAs, five downregulated circRNAs (circRNA00447, circRNA00747, circRNA06661, circRNA00723, and circRNA01605) that interacted with downregulated miRNAs, and circRNAs that exhibited double interactions with upregulated or downregulated miRNAs (Figure 4C; Supplementary Table 5). Moreover, based on higher betweenness centrality and closeness centrality in the network, it could be deduced that a single circRNA might potentially interrelate with one or more miRNAs, and that one miRNA might be simultaneously affected by multiple circRNAs. For instance, circRNA00723 potentially interacted with miR-466i-5p, miR-5623-5p, miR-7058-5p, and miR-669f-5p. Conversely, miR-3971 might be co-connected with circRNA03673, circRNA06452, and circRNA08440.

### Functional enrichment analyses

To analyze the functional annotation of these circRNAs, the differentially upregulated and downregulated circRNAs were separately implemented to GO and KEGG analysis. There were 417 target miRNAs of upregulated circRNAs, and 2067 target mRNAs were obtained by merging predicted mRNAs from the miRDB and TargetScan databases. In addition, 2,297 overlapping mRNAs from the above two databases were identified for the 413 target miRNAs of downregulated circRNAs.

**Figure 5:**
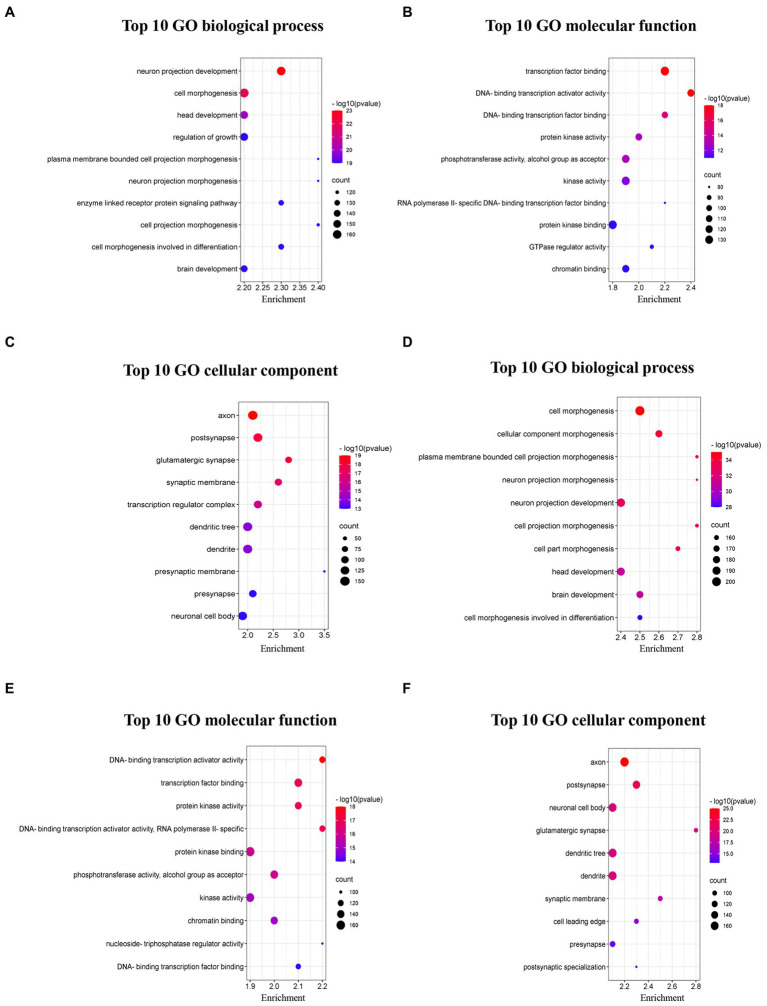
Top 10 GO pathways of dysregulated circRNAs in 5 × FAD mice. **(A)** Biological processes of upregulated circRNAs. **(B)** Molecular function of upregulated circRNAs. **(C)** Cellular component of upregulated circRNAs. **(D)** Biological processes of downregulated circRNAs. **(E)** Molecular function of downregulated circRNAs. **(F)** Cellular component of downregulated circRNAs. The bubble size represents the number of enriched genes, while the bubble color represents the significance of enrichment based on the corrected value of *p*.

The top 10 GO terms from the BP sub-ontologies showed that the aberrantly upregulated circRNAs were mostly involved in neuron projection development (GO:0031175), cell morphogenesis (GO:0000902), and head development (GO:0060322; Figure 5A). The MF sub-ontologies were mainly related to transcription factor binding (GO:0008134), DNA-binding transcription activator activity (GO:0001216), and DNA-binding transcription factor binding for upregulated circRNAs (GO:0140297; Figure 5B). Moreover, CC sub-ontologies were mostly enriched in the axon (GO:0030424), the postsynapse (GO:0098794), and the glutamatergic synapse for aberrantly upregulated circRNAs (GO:0098978; Figure 5C). The downregulated circRNAs were mostly enriched in cell morphogenesis (GO:0000902), plasma membrane-bounded cell projection morphogenesis (GO:0120039), and cellular component morphogenesis (GO:0032989) in the BP sub-ontologies (Figure 5D), DNA-binding transcription activator activity (GO:0001216), transcription factor binding (GO:0008134), and protein kinase binding (GO:0004672) in the MF sub-ontologies (Figure 5E), and the axon (GO:0030424), post-synapse (GO:0098794), and neuronal cell body (GO:0043025) in the CC sub-ontologies (Figure 5F).

**Figure 6:**
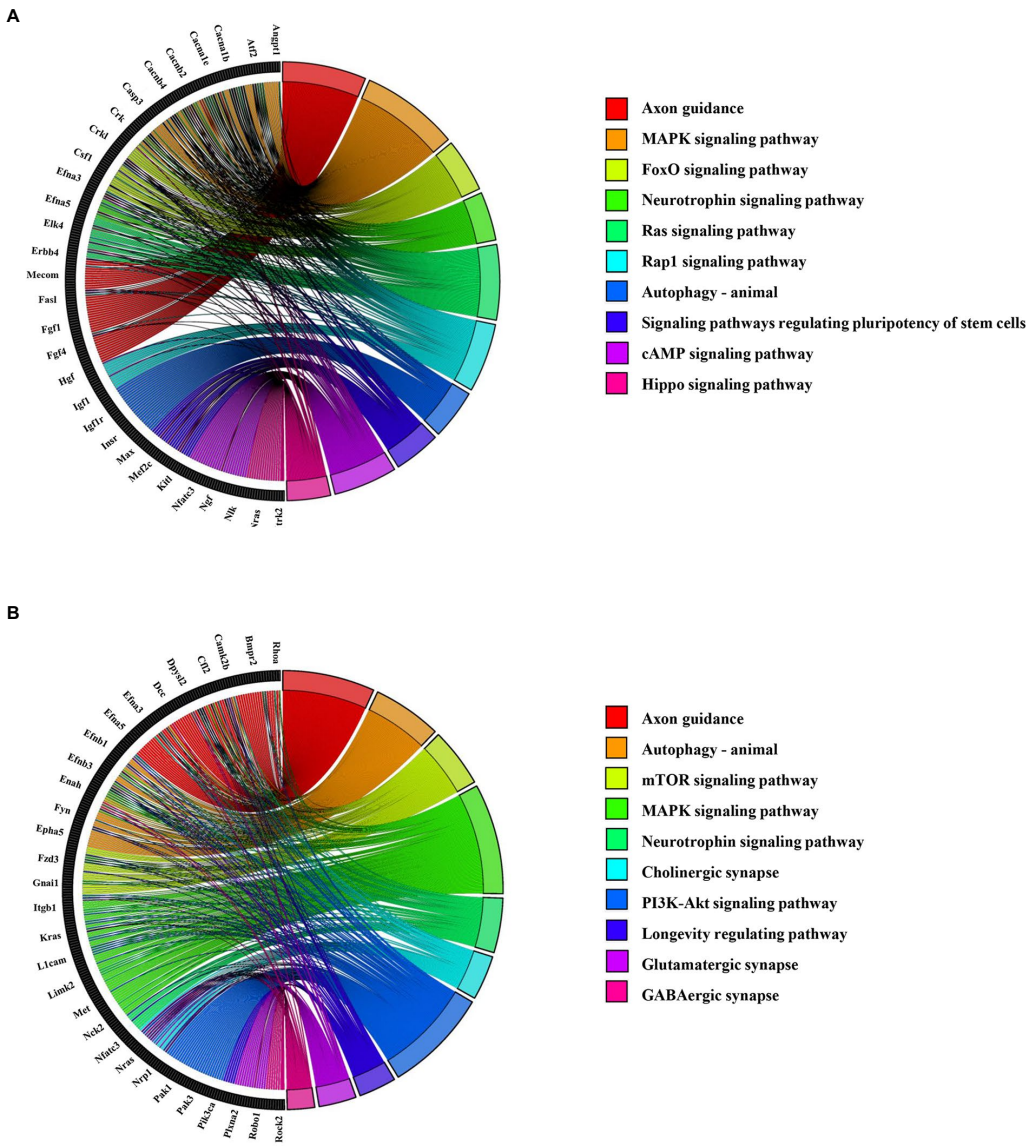
Top 10 KEGG enrichment pathways for the abnormally expressed circRNAs. **(A)** KEGG pathways for upregulated circRNA-related mRNAs. The left section shows the circRNA-related mRNA name, and the right section shows the top 10 pathways. **(B)** KEGG pathways for downregulated circRNA-related mRNAs. The left section shows the circRNA-related mRNAs, and the right section shows the top 10 KEGG pathways.

KEGG pathway analysis revealed that axon guidance (mmu04360), MAPK signaling pathway (mmu04010), and Neurotrophin signaling pathway (mmu04722) were closely linked to the target gene of abnormally upregulated circRNAs (Figure 6A; Supplementary Table 6). Additionally, axon guidance (mmu04360), autophagy (mmu04140), and the mTOR signaling pathway (mmu04150) were closely connected with the target gene of abnormally downregulated circRNAs (Figure 6B; Supplementary Table 6).

### Construction of ceRNA network

**Figure 7:**
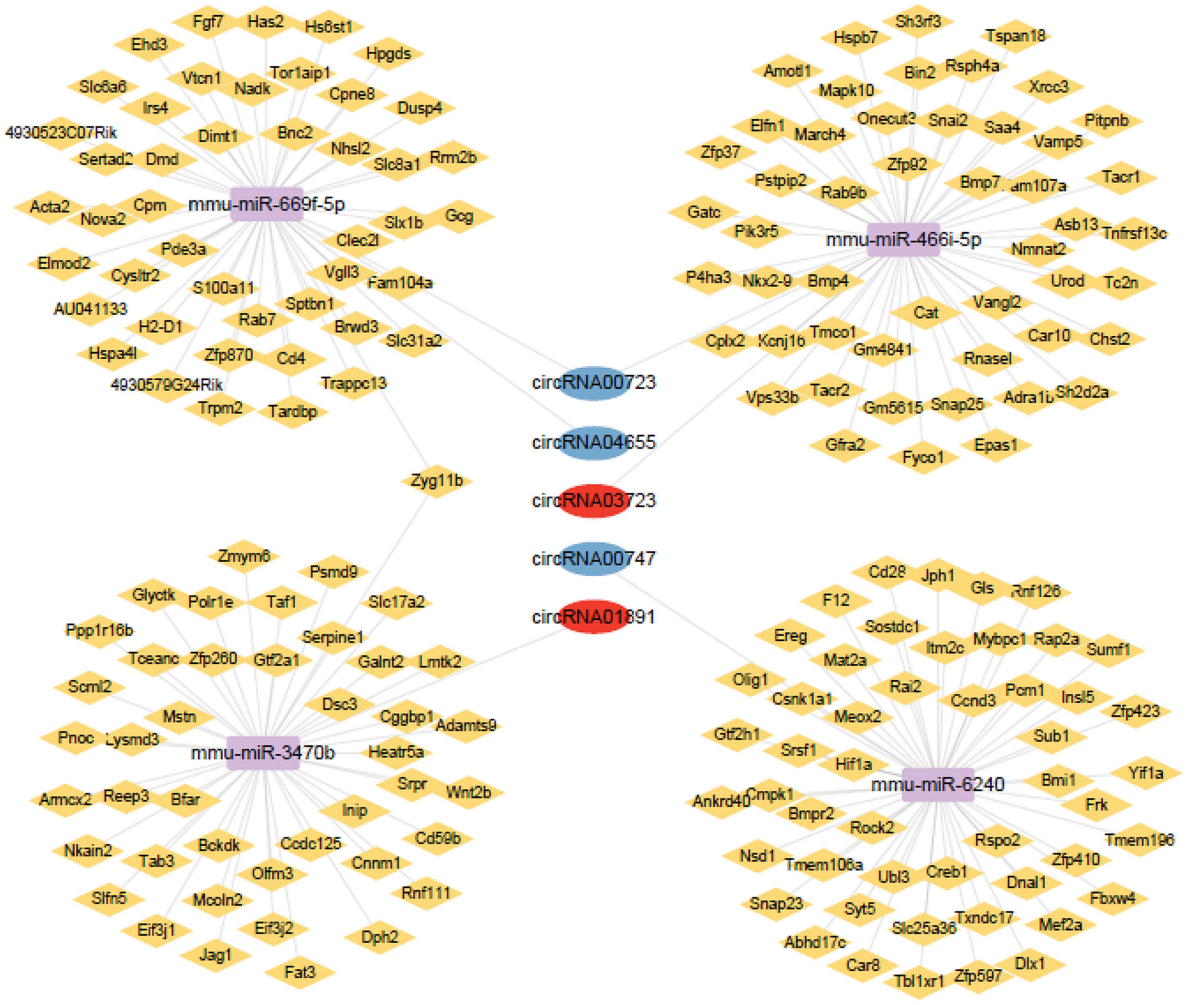
Network of circRNAs-miRNAs-mRNAs. Upregulated circRNAs are shown using red circles, downregulated circRNAs are shown using blue circles, potential miRNAs are shown using purple squares, and potential mRNAs are shown using yellow rhombus.

ceRNA networks accurately and intuitively reflect the interactions among circRNAs, miRNAs, and mRNA. We established a ceRNA network using the top five circRNAs with the highest intermediate centrality and intimacy centrality values that have not been reported to have an association with AD. To establish a ceRNA network centered on circRNA00723, circRNA04655, circRNA01891, circRNA03723, and circRNA00747, we screened for aberrantly expressed miRNAs in RNA sequencing (Table 3). We identified six circRNA-miRNA interactions involving four miRNAs and five circRNAs (Table 4). Next, we identified 188 target mRNAs by merging the predicted mRNAs for these four miRNAs using the miRDB and TargetScan databases. In addition, we integrated the five circRNAs, four miRNAs, and 188 target mRNAs into a circRNA–miRNA–mRNA network (Figure 7).

**Table 3:** Information of differentially expressed miRNAs.

miRNAs	log2Fold Change	*P*-value	Regulation
miR-33-5p	1.059582	0.028052	Up
miR-3470b	1.121627	0.01195	Up
miR-3963	1.120086	0.031801	Up
miR-466i-5p	1.685278	0.001582	Up
miR-490-5p	1.036711	0.018928	Up
miR-511-3p	1.199429	0.011024	Up
miR-5121	4.467184	9.38E-09	Up
miR-6240	1.683446	0.009093	Up
miR-669f-5p	1.835861	0.044532	Up
miR-1839-3p	−1.68766	0.034451	Down
miR-1964-3p	−1.35741	0.037749	Down
miR-1969	−2.56641	0.008142	Down

**Table 4:** Interactions of aberrantly expressed circRNAs and miRNAs.

cicrRNAs	Regulation	miRNAs	Regulation
circRNA04655	Down	miR-669f-5p	Up
circRNA00723	Down	miR-669f-5p	Up
circRNA00723	Down	miR-466i-5p	Up
circRNA03723	Up	miR-466i-5p	Up
circRNA00747	Down	miR-6240	Up
circRNA01891	Up	miR-3470b	Up

### Expression verification of the circRNAs, miRNAs, and mRNAs in the hippocampus of 5 × FAD mice

**Figure 8:**
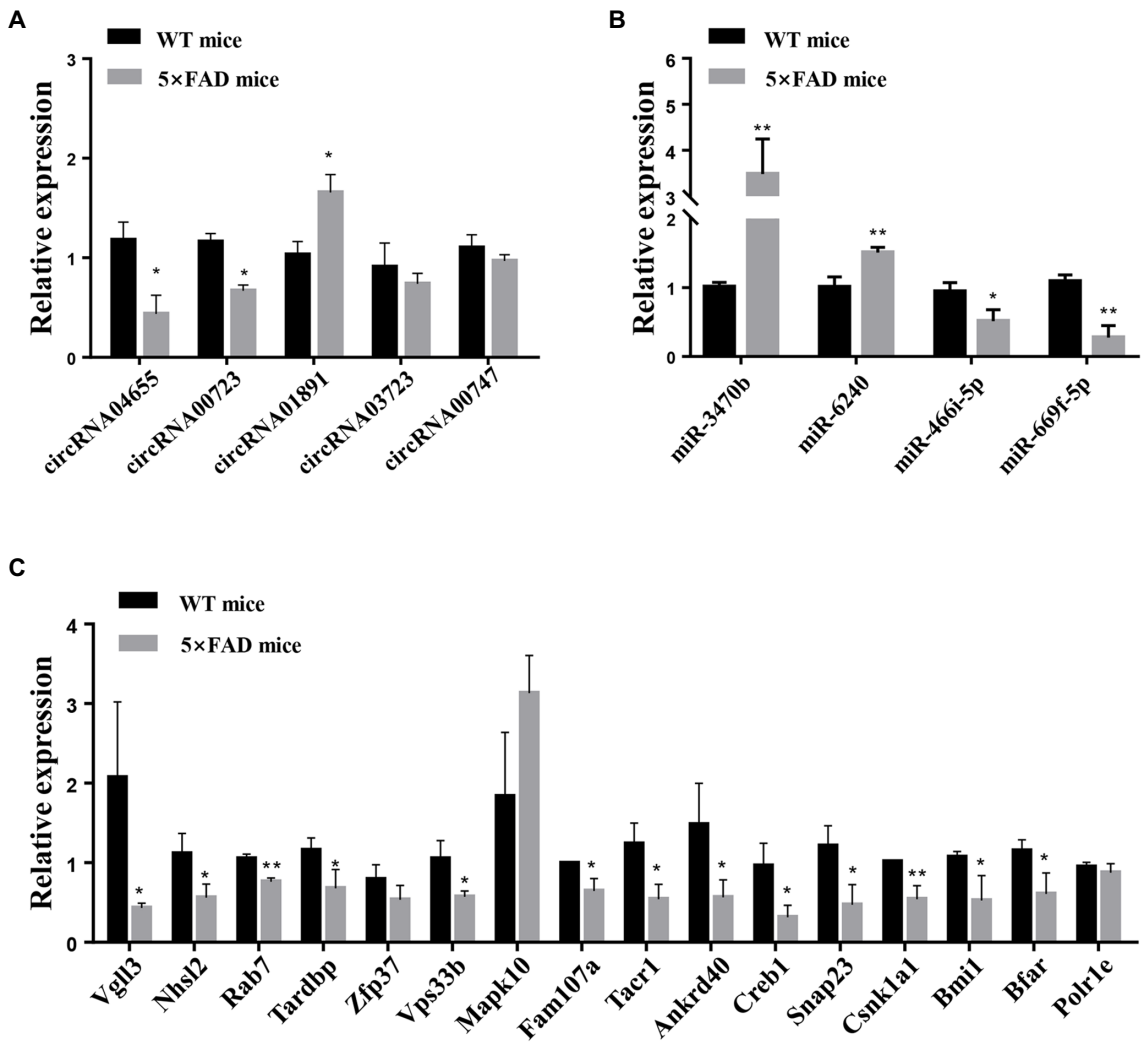
qRT-PCR analysis of circRNAs, miRNAs, and mRNAs. **(A)** Expression levels of circRNAs, including circRNA04655, circRNA00723, circRNA01891, circRNA03723, and circRNA00747, in the hippocampus of 5 × FAD mice and WT mice. **(B)** Expression of miRNAs, including miR-3470b, miR-6240, miR-466i-5p, and miR-669f-5p, in the hippocampus of 5 × FAD mice and WT mice. **(C)** Expression of mRNAs, including *Vgll3*, *Nhsl2*, *Rab7*, *Tardbp*, *Zfp37*, *Vps33b*, *Mapk10*, *Fam107a*, *Tacr1*, *Ankrd40*, *Creb1*, *Snap23*, *Csnk1a1*, *Bmi1*, *Bfar*, and *Polr1e*, in the hippocampus of 5 × FAD mice and WT mice. Results are shown as mean ± SD (*n* = 3). *p < 0.05, **p < 0.01 vs. WT mice.

To investigate the potential relationship within the predicted circRNA–miRNA–mRNA network, qRT-PCR analysis was used to verify circRNA, miRNA, and mRNA expression in the hippocampus of 5 × FAD mice. Consistent with RNA sequencing results, the expression of circRNA04655 and circRNA00723 in the hippocampus of 5 × FAD mice was significantly decreased (*p* = 0.045, *p* = 0.023 vs. WT mice; Figure 8A), and the expression of circRNA01891 was increased (*p* = 0.048 vs. WT mice; Figure 8A). The expression of circRNA00747 was reduced in the hippocampus of 5 × FAD mice, which had a similar trend to the sequencing data, but the statistical difference compared to WT mice was insignificant (Figure 8A). The expression of circRNA03723 was insignificantly reduced in the hippocampus of 5 × FAD mice, and the expression trend was inconsonant with its RNA sequencing result (Figure 8A). miR-3470b and miR-6240 were significantly upregulated in the hippocampus of 5 × FAD mice (*p* = 0.0049, *p* = 0.0062 vs. WT mice; Figure 8B), and the expression alteration was in line with the sequencing results. However, miR-669f-5p and miR-466i-5p were significantly downregulated in the hippocampus of 5 × FAD mice (*p* = 0.0018, *p* = 0.0239 vs. WT mice; Figure 8B), deviating from the sequencing results. By intersecting the mRNAs in the circRNA–miRNA–mRNA network (Figure 7) with the differential expressed mRNAs identified through sequencing, we identified 16 overlapping mRNAs, which presented the opposite trend with miRNAs. Consistent with sequencing results, the expression level of 13 mRNAs was significantly decreased in the hippocampus of 5 × FAD mice, including *Vgll3*, *Nhsl2*, *Rab7*, *Tardbp*, *Vps33b*, *Fam107a*, *Tacr1*, *Ankrd40*, *Creb1*, *Snap23*, *Csnk1a1*, *Bmi1*, and *Bfar* (*p* = 0.041, *p* = 0.035, *p* = 0.003, *p* = 0.041, *p* = 0.025, *p* = 0.019, *p* = 0.019, *p* = 0.035, *p* = 0.048, *p* = 0.024, *p* = 0.022, *p* = 0.008, *p* = 0.034, vs. WT mice; Figure 8C). Among the remaining three mRNAs, *Zfp37* and *Polr1e* were downregulated and *Mapk10* was upregulated in the hippocampus of 5 × FAD mice, but the differences were insignificant (Figure 8C). According to the qRT-PCR analysis, the *Zfp37* and *Polr1e* had similar expression trends to the sequencing data, while *Mapk10* had an inconsonant expression trend to the sequencing data. Overall, the qRT-PCR results of three circRNAs, two miRNAs, and 13 mRNAs were consistent with the RNA sequencing data.

## Discussion

AD has enormous and detrimental impacts on the elderly, not only limited to cognitive impairment but also the shadow of developing dementia (Xia et al., 2018). While the etiopathogenesis of AD is complex with many contributing factors, including (but not limited to) genetic and environmental factors, many important details of AD pathogenesis remain unknown. Recently, the properties of non-coding RNAs in AD pathogenesis have attracted considerable attention, especially miRNAs and long non-coding RNAs. However, the properties of circRNAs during AD progression are still poorly understood. We reasoned that RNA sequencing in conjunction with a systematic analysis of the profiles of circRNAs dysregulated in AD could support novel foresight into comprehending the pathophysiology of AD. This study presents for the first time a profile of circRNA in the hippocampus of 5 × FAD mice, an investigation of the functional roles of these dysregulated circRNAs, and the construction of a circRNA-related ceRNA network.

Although dysregulated circRNAs in the senescence-accelerated mouse-prone 8 (SAMP8) mice and the precursor protein (APP) and presenilin 1 (PS1) double transgenic mice have been revealed in several investigations using RNA sequencing or microarrays (Zhang et al., 2017; Ma et al., 2019; Zhang Y. et al., 2021), circRNA expression profiles have not been determined in the brain of 5 × FAD mice, the transgenic mice that co-express a total of five familial AD mutations (Oakley et al., 2006). Because of these mutations, 5 × FAD mice present very similar clinicopathological characteristics to clinical patients. Furthermore, 5 × FAD mice demonstrate clinicopathological characteristics of AD at a younger age (in comparison with other transgenic mice). In 5 × FAD mice, aggregation of amyloid-beta peptide_42_ (Aβ_42_) in neurons occurs at 6 weeks of age, amyloid deposition and gliosis appear in the subiculum and deep cortical layers at 2 months, loss of synaptic proteins and damage to the hippocampus begin at 4 months, and impaired cognition is observed thereafter (Oakley et al., 2006). In line with these observations, 5 × FAD mice exhibit restricted learning capabilities and a decline in hippocampus-dependent memory (compared to age-matched WT mice) in our finding. Hence, 5 × FAD mice are an ideal model for RNA sequencing analyses of AD pathogenesis.

So far as we know, there has never been a report on RNA sequencing of circRNA profiles in 5 × FAD mice. Our results reveal that 34 circRNAs were significantly dysregulated in the hippocampus of 5 × FAD mice, including 17 upregulated and 17 downregulated circRNAs. Since circRNAs are transformed from host linear RNAs, the biological function of the host gene may provide clues concerning the function of the circRNA. Some host genes of circRNAs directly or indirectly participated in the pathological process of AD, including circRNA03556 (*Hepacam*), circRNA00723 (*Myt1*), circRNA02418 (*Cntln*), and circRNA04655 (*Elavl2*). Research on the App^NL-F^ mouse model of AD provided evidence that the hepacam protein has a marked increase in early-stage AD mice, as well as that there was a significant increase in its colocalization with Aβ (Aladeokin et al., 2019). Some study has proposed that *Myt1* affects cholesterol 24S-hydroxylase (CYP46A1) gene transcription and is correlated with AD (Li et al., 2010). Hohman’s group revealed that the gene levels of *CNTLN* in the heart are associated with cognitive decline dependent on amyloid (Hohman et al., 2017). Lastly, neuronal ELAVL proteins modulate the regulation of Aβ precursor protein steady-state levels by inducing neuron-specific alternative splicing, alterations that have been linked to AD (Fragkouli et al., 2017).

Several differentially expressed circRNAs and miRNAs were found to be altered in the brain of different AD model mice (Supplementary Table 7). circRNA00825 and circRNA01985 were aberrantly upregulated in the brain of APP/PS1 mice (Ma et al., 2019; Wei et al., 2022). In aged C57BL/6J mice, circRNA03073 and circRNA08440 were found to have aberrant downregulation in the hippocampus of mice (Ran et al., 2022). However, the abnormal circRNAs found in the brains of SAMP8 mice and Tg2576 transgenic AD mice are different from those in the hippocampus of 5 × FAD mice in our study(Lee et al., 2019; Zhang Y. et al., 2021). This phenomenon may be related to the species differences of the animals. Although there are no identical circRNAs found in our study and other circRNA analyses, the KEGG pathways are found to be highly similar to the present study, including axon guidance, cAMP signaling pathway, and Hippo signaling pathway (Zhang Y. et al., 2021). Additionally, the aberrantly expressed miRNAs in the present study were found to be dysregulated in the hippocampus of APP/PS1 mice, Tg2576 mice, and Tg6799 mice, including miR-669f-5p, miR-6240, miR-1969, miR-3963, and miR-490-5p (Noh et al., 2014; Ma et al., 2019).

According to KEGG enrichment analysis, circRNAs that were dysregulated in the hippocampus of 5 × FAD mice are involved in numerous signaling pathways, several of which have been shown to be necessary for AD pathogenesis, including neurotrophin signaling pathway, axon guidance, the MAPK signaling pathway, and the FoxO signaling pathway. Moreover, a broad spectrum of cellular functions (e.g., proliferation, differentiation, migration, apoptosis, and autophagy) are regulated by these signaling pathways. Axon guidance molecules are essential in neural circuit formation (Charron, 2018). A recent study found that axon guidance molecules have effects on Aβ expression and tau protein hyperphosphorylation during the occurrence and process of AD (Zhang L. et al., 2021). Our research showed that upregulated and downregulated circRNAs both modulated the neurotrophin signaling pathway, which has been known to be affected in AD. During the neurotrophin signaling pathway, brain-derived neurotrophic factors ameliorate cognitive impairment and neuronal damage in AD (Gao et al., 2022; Liu et al., 2022). Enriched GO terms uncovered that dysregulated circRNAs were mainly involved in neuron projection development, cell morphogenesis, head development, and cellular component morphogenesis. The factors that influence neuron projection development and cell morphogenesis may contribute to AD occurrence.

circRNAs and miRNAs are both potentially key regulators of multiple aspects of AD. circRNAs have strong effects on regulating transcription and translation by sponging miRNAs. Considering the importance of the ceRNA hypothesis for non-coding RNAs, we first identified circRNA−miRNA interactions in 5 × FAD mice. We revealed that many circRNAs could co-associate with more than one miRNA. For example, circRNA00533 might interact with both miR-146a and miR-34c-3p, and the signaling pathways associated with these miRNAs were predicted to have a significant dysregulation in AD (Müller et al., 2014; Nikolac Perkovic et al., 2021). Next, potential target genes were added to generate a circRNA-miRNA-mRNA ceRNA network that consisted of five circRNAs, four miRNAs, and 188 mRNAs for AD. qRT-PCR was utilized to measure the level of circRNAs (circRNA00723, circRNA04655, circRNA01891, circRNA03723, and circRNA00747), miRNAs (miR-3470b, miR-6240, miR-669f-5p, and miR-466i-5p) and mRNAs (*Vgll3*, *Nhsl2*, *Rab7*, *Tardbp*, *Vps33b*, *Fam107a*, *Tacr1*, *Ankrd40*, *Creb1*, *Snap23*, *Csnk1a1*, *Zfp37*, *Polr1e*, *Mapk10*, *Bmi1*, and *Bfar*). The differential expression of circRNA00723, circRNA04655, and circRNA01891 in the hippocampus of 5 × FAD mice using qRT-PCR analyses coincided with their RNA sequencing results. Although these differentially expressed circRNAs (circRNA00723, circRNA04655, circRNA03723, and circRNA00747) have not previously been reported, their potential regulatory roles in AD could be inferred from the signaling pathways of their interacting transcripts. These pathways included the cGMP-PKG signaling pathway, hippo signaling pathway, and the Wnt signaling pathway. The expression changes of two miRNAs (miR-3470b and miR-6240) and 13 mRNAs (*Vgll3*, *Nhsl2*, *Rab7*, *Tardbp*, *Vps33b*, *Fam107a*, *Tacr1*, *Ankrd40*, *Creb1*, *Snap23*, *Csnk1a1*, *Bmi1*, and *Bfar*) in the hippocampus of 5 × FAD mice using qRT-PCR analyses had consistency with the RNA sequencing results, while the expression changes of miR-669f-5p and miR-466i-5p were inconsonant. The statistical differences of another two circRNAs (circRNA03723 and circRNA00747) and three mRNAs (*Zfp37*, *Polr1e,* and *Mapk10*) expression levels were insignificant in the hippocampus of 5 × FAD mice compared to WT mice using qRT-PCR analyses. Among them, circRNA00747, *Zfp37*, and *Polr1e* had similar expression trends to the sequencing data. Most of the RNAs for expression verification using qRT-PCR were in line with the RNA sequencing results, and a few were biased. We speculate that this discordant expression of a few RNAs may be related to the differential experimental steps and analytical methods between the two methods and the small sample size. The data obtained from both methods underwent multiple experimental and analytical steps, such as library construction, sequencing, and data processing. The qRT-PCR results can be affected by amplification region and efficiency, and RNA sequencing results can be affected by GC preference, exon number, and gene length. As a small sample size was included in this study, the error caused by different methods were not eliminated. It is generally accepted that there is 80% concordance in RNA sequencing and qRT-PCR assay (Consortium, 2014; Robert and Watson, 2015; Everaert et al., 2017). The qRT-PCR analyses demonstrated that the majority of RNAs in the circRNA–miRNA–mRNA ceRNA network had good consistency with the sequencing results regarding the differential expressions and alteration trends.

miR-6240, a predicted target of circRNA00747 (contrary to the expression trend for circRNA00747), is aberrantly upregulated in 5 × FAD mice. A recent study revealed that miR-6240 showed functional conservation in cardiomyocyte proliferation and mitosis (Adamowicz et al., 2018). Many target genes of miR-6240 have been proposed to be related to AD etiopathogenesis, including serine and arginine-rich splicing factor 1 (*SRSF1*), hypoxia-inducible factor-1α (*HIF1α*), mesenchyme homeobox 2 (*MEOX2*), cAMP-responsive element-binding protein 1 (*CREB1*), pericentriolar material-1 (*PCM1*), coiled-coil containing protein kinases (*ROCK2*), synaptosomal-associated protein 23 (*SNAP23*), and myocyte enhancer factor 2A (*MEF2A*). These genes are involved in plaque deposition, tau protein biosynthesis, neuronal dysfunction, neuroinflammation, autophagy, and other pathological processes related to AD (Chakravarthy et al., 2013; Soto et al., 2016; Muhammad et al., 2019; Adeyemi et al., 2021; Li et al., 2021; Weber and Herskowitz, 2021). SRSF1 is a member of the serine/arginine-rich proteins family, which is involved in the process of exon selection and mRNA output and localization. Recent studies have revealed that SRSF1 promotes tau mRNA formation (Jakubauskienė et al., 2021). Microtubule-associated protein tau, expressed mainly in axons, regulates the aggregation and stability of microtubules (Gao et al., 2018). When tau protein is hyperphosphorylated, it dissociates from the axon microtubule, misfolds, and aggregates, eventually forming neurofibrillary tangles, considered one of the emblematic hallmarks of AD (Chong et al., 2018; Naseri et al., 2019). Nevertheless, regulation of these interactions by circRNAs-miRNAs-mRNAs networks in AD has not been investigated. Additional research on ceRNA networks will help expand our knowledge of their biological functions and help us to understand their regulatory roles in AD pathogenesis.

Our study has two limitations. Firstly, to obtain a more accurate expression profile of circRNAs, more samples should be analyzed, including samples from different animal models of AD, and blood and tissue samples from AD patients. Secondly, the interactions and underlying mechanisms of these identified circRNAs, miRNAs, and mRNAs need to be further investigated.

## Conclusion

For the first time, this study revealed dysregulated expression profiles of circRNAs in the hippocampus of 5 × FAD mice using RNA sequencing. From this data, we identified the potential biological functions of these circRNAs and their signaling pathways in AD. In addition, we have constructed a ceRNA network centered on circRNAs and verified key circRNA, miRNA, and mRNA expressions using qRT-PCR. Our findings should broaden our knowledge of circRNA regulatory action in the pathogenesis of AD.

## Data availability statement

The datasets presented in this study can be found in online repositories. The names of the repository/repositories and accession number(s) can be found at: Data have been deposited in Gene Expression Omnibus (GEO) with the accession code GSE206562.

## Ethics statement

The animal study was reviewed and approved by Experimental Animal Care and Use Committee of the Institute of Medicinal Biotechnology.

## Author contributions

RL and ZL: concept and design, manuscript editing, and manuscript review. RL: definition of intellectual content. TS: literature search and manuscript preparation. TS and LZ: experimental studies. TS and ZC: data acquisition and analysis. QL: statistical analysis. All authors contributed to the article and approved the submitted version.

## Funding

This study was supported by the National Natural Science Foundation of China (Nos. U1803281, 82173806, and 82204366), Chinese Academy of Medical Sciences (CAMS) Innovation Fund for Medical Science (2021-1-I2M-030 and 2022-I2M-2-002), and Non-profit Central Research Institute Fund of Chinese Academy of Medical Sciences (2022-JKCS-08 and 3332022054).

## Conflict of interest

The authors declare that the research was conducted in the absence of any commercial or financial relationships that could be construed as a potential conflict of interest.

## Publisher’s note

All claims expressed in this article are solely those of the authors and do not necessarily represent those of their affiliated organizations, or those of the publisher, the editors and the reviewers. Any product that may be evaluated in this article, or claim that may be made by its manufacturer, is not guaranteed or endorsed by the publisher.

## Footnotes

^1^ http://circatlas.biols.ac.cn/

^2^ http://www.microrna.org/

^3^ http://www.mirdb.org/

^4^ http://www.targetscan.org/

^5^ http://www.geneontology.org

^6^ https://www.kegg.jp/

^7^ http://circatlas.biols.ac.cn/

^8^ http://circrna.org/
