# Supplementary material for: Comprehensive analysis of dysregulated circular RNAs and construction of a ceRNA network involved in the pathology of Alzheimer’s disease in a 5 × FAD mouse model
Source: Front Aging Neurosci. 2022 Nov 17;14:1020699. doi: 10.3389/fnagi.2022.1020699 (PMC9712785; doi:10.3389/fnagi.2022.1020699)
Supplement: Supplementary file 1 [file Data_Sheet_1.docx]

Supplementary Material

# Supplementary Data

**
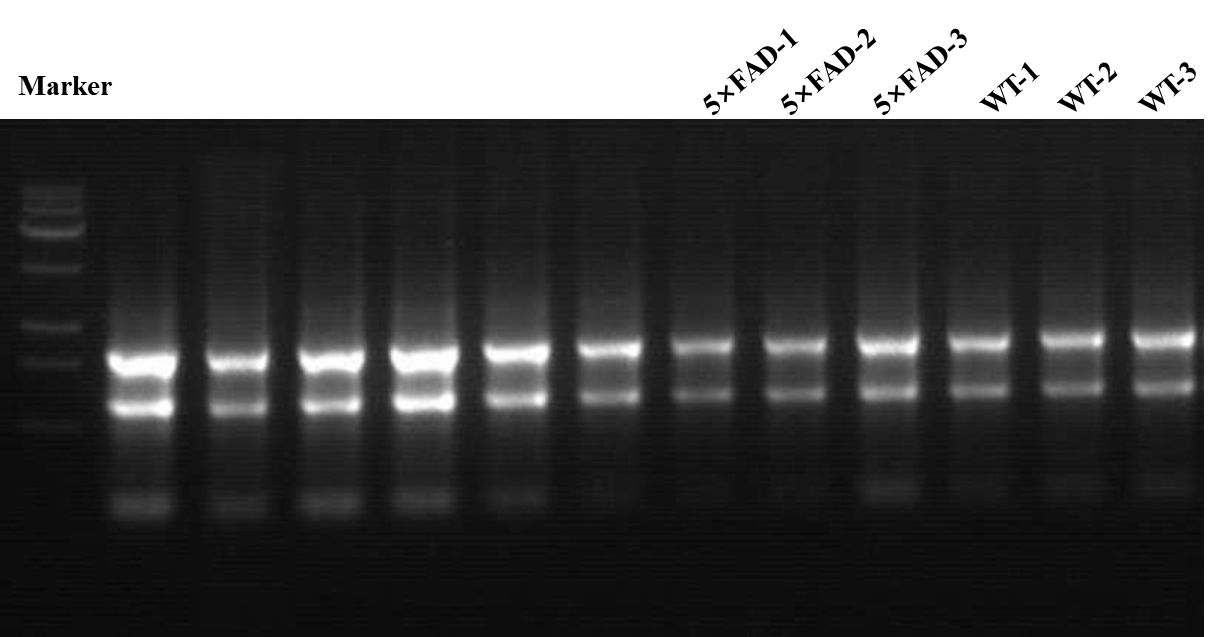
**

**Supplementary Figure 1 The gel electrophoresis of RNA.**

**
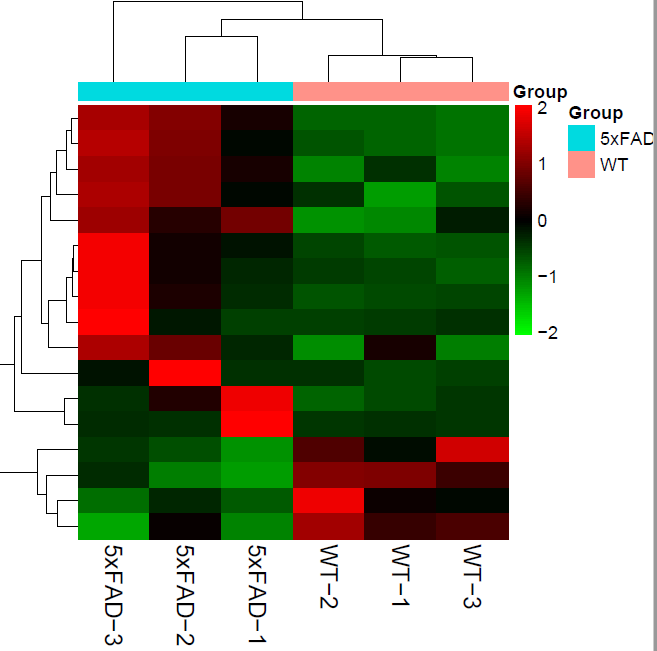
**

**Supplementary Figure 2 The heatmap of the differently expressed miRNAs in the hippocampus of 5×FAD mice.**

**Supplementary Table 1 The RNA concentration of the RNA sequencing**

| RNA sample | concentration (ng/ul) |
| --- | --- |
| 5×FAD-1 | 655 |
| 5×FAD -2 | 1040 |
| 5×FAD -3 | 1485 |
| WT-1 | 1155 |
| WT-2 | 965 |
| WT-3 | 1300 |

**Supplementary Table 2 Predicted miRNAs with circatlas database and miRanda database**

| **circRNAs** | **Target miRNAs** |
| --- | --- |
| circRNA03556 | mmu-miR-1903, mmu-miR-3103-5p, mmu-miR-6965-5p, mmu-miR-12183-5p, mmu-miR-214-3p, mmu-miR-761, mmu-miR-12195-3p, mmu-miR-7231-3p, mmu-miR-207, mmu-miR-384-3p, mmu-miR-7054-3p, mmu-miR-7032-5p, mmu-miR-6915-3p, mmu-miR-19a-5p, mmu-miR-686, mmu-miR-6990-5p, mmu-miR-1970c-5p, mmu-miR-6961-3p, mmu-miR-19b-2-5p, mmu-miR-8097, mmu-miR-29a-5p, mmu-miR-101b-5p, mmu-miR-3059-5p, mmu-miR-666-5p, mmu-miR-6916-5p, mmu-miR-6925-5p, mmu-miR-7017-5p, mmu-miR-877-3p, mmu-miR-7078-3p, mmu-miR-19b-1-5p |
| circRNA03725 | mmu-miR-7094-1-5p, mmu-miR-7647-3p, mmu-miR-181b-5p, mmu-miR-7094-3p, mmu-miR-181d-5p, mmu-miR-9768-3p, mmu-miR-664-3p, mmu-miR-6970-5p, mmu-miR-7689-5p, mmu-miR-12205-5p, mmu-miR-6414, mmu-miR-3068-5p, mmu-miR-125a-3p, mmu-miR-7116-3p, mmu-miR-181a-5p, mmu-miR-7b-5p, mmu-miR-146b-5p, mmu-miR-669g, mmu-miR-683, mmu-miR-871-3p, mmu-miR-34b-3p, mmu-miR-6481, mmu-miR-141-5p, mmu-miR-599, mmu-miR-6385, mmu-miR-7685-5p, mmu-miR-532-5p, mmu-miR-1904, mmu-miR-6386, mmu-miR-30b-3p |
| circRNA01979 | mmu-miR-107-5p, mmu-miR-6946-3p, mmu-miR-1903, mmu-miR-103-1-5p, mmu-miR-103-2-5p, mmu-miR-3089-3p, mmu-miR-6348, mmu-miR-6999-3p, mmu-miR-5625-5p, mmu-miR-3099-5p, mmu-miR-320-5p, mmu-miR-684, mmu-miR-6344, mmu-miR-7231-3p, mmu-miR-12183-5p, mmu-miR-3098-3p, mmu-miR-7116-3p, mmu-miR-6932-3p, mmu-miR-3092-5p, mmu-miR-7092-3p, mmu-miR-1190, mmu-miR-6935-3p, mmu-miR-1968-5p, mmu-miR-7083-3p, mmu-miR-693-3p, mmu-miR-207, mmu-miR-7b-5p, mmu-miR-7014-3p, mmu-miR-1955-5p, mmu-miR-7028-3p |
| circRNA00533 | mmu-miR-7680-5p, mmu-miR-883b-3p, mmu-miR-883a-3p, mmu-miR-5104, mmu-miR-30d-3p, mmu-miR-30e-3p, mmu-miR-15b-3p, mmu-miR-34b-3p, mmu-miR-5619-5p, mmu-miR-290b-3p, mmu-miR-6963-5p, mmu-miR-6991-3p, mmu-miR-7013-5p, mmu-miR-124-3p, mmu-miR-146b-5p, mmu-miR-6951-5p, mmu-miR-12181-3p, mmu-miR-6901-3p, mmu-miR-7662-3p, mmu-miR-761, mmu-miR-145b, mmu-miR-3068-3p, mmu-miR-218-5p, mmu-miR-7659-5p, mmu-miR-7663-5p, mmu-miR-34c-3p, mmu-miR-145a-5p, mmu-miR-146a-5p, mmu-miR-6999-5p, mmu-miR-7045-5p |
| circRNA01891 | mmu-miR-6946-3p, mmu-miR-145a-3p, mmu-miR-7116-3p, mmu-miR-7092-3p, mmu-miR-107-5p, mmu-miR-26a-2-3p, mmu-miR-7059-3p, mmu-miR-103-1-5p, mmu-miR-103-2-5p, mmu-miR-691, mmu-miR-7236-5p, mmu-miR-7231-3p, mmu-miR-130a-5p, mmu-miR-6932-3p, mmu-miR-3081-5p, mmu-miR-6951-3p, mmu-miR-6967-3p, mmu-miR-6935-3p, mmu-miR-3074-5p, mmu-miR-324-3p, mmu-miR-9-3p, mmu-miR-7008-3p, mmu-miR-710, mmu-miR-12202-3p, mmu-miR-494-3p, mmu-miR-3068-5p, mmu-miR-7084-3p, mmu-miR-206-5p, mmu-miR-3470a, mmu-miR-5098 |
| circRNA03723 | mmu-miR-466b-5p, mmu-miR-466o-5p, mmu-miR-7210-3p, mmu-miR-466p-5p, mmu-miR-7661-5p, mmu-miR-466i-5p, mmu-miR-6970-5p, mmu-miR-3112-3p, mmu-miR-3068-5p, mmu-miR-297c-5p, mmu-miR-1187, mmu-miR-297a-5p, mmu-miR-297b-5p, mmu-miR-429-5p, mmu-miR-6984-3p, mmu-miR-6414, mmu-let-7f-5p, mmu-miR-466l-3p, mmu-miR-466c-5p, mmu-miR-466l-5p, mmu-miR-466a-5p, mmu-miR-466e-5p, mmu-miR-7093-5p, mmu-let-7b-5p, mmu-miR-6925-5p, mmu-miR-466a-3p, mmu-miR-466e-3p, mmu-miR-7094-1-5p, mmu-let-7e-5p, mmu-miR-3475-3p |
| circRNA04809 | mmu-miR-6998-3p, mmu-miR-667-5p, mmu-miR-708-5p, mmu-miR-7651-5p, mmu-miR-320-5p, mmu-miR-432, mmu-miR-7030-5p, mmu-miR-6921-5p, mmu-miR-6899-3p, mmu-miR-7688-3p, mmu-miR-7075-5p, mmu-miR-6948-3p, mmu-miR-6958-3p, mmu-miR-28a-5p, mmu-miR-6938-5p, mmu-miR-7117-3p, mmu-miR-881-3p, mmu-miR-23b-5p, mmu-miR-7090-5p, mmu-miR-7654-3p, mmu-miR-1903, mmu-miR-6919-3p, mmu-miR-6927-3p, mmu-miR-7652-3p, mmu-miR-7656-3p, mmu-miR-7663-5p, mmu-miR-12183-5p, mmu-miR-3966, mmu-miR-7226-5p, mmu-miR-7017-3p |
| circRNA00343, | mmu-miR-6388, mmu-miR-3081-5p, mmu-miR-7025-5p, mmu-miR-669d-5p, mmu-miR-8097, mmu-miR-290b-3p, mmu-miR-22-3p, mmu-miR-135a-5p, mmu-miR-135b-5p, mmu-miR-216b-3p, mmu-miR-7051-3p, mmu-miR-380-3p, mmu-miR-7031-5p, mmu-miR-7050-3p, mmu-miR-6393, mmu-miR-376c-3p, mmu-miR-7683-3p, mmu-miR-297a-5p, mmu-miR-297c-5p, mmu-miR-345-3p, mmu-miR-742-5p, mmu-miR-7027-5p, mmu-miR-9768-5p, mmu-miR-204-3p, mmu-miR-7689-3p, mmu-miR-3472, mmu-miR-7010-5p, mmu-miR-298-3p, mmu-miR-873a-5p, mmu-miR-467e-5p |
| circRNA03712, | mmu-miR-5620-3p, mmu-miR-3618-3p, mmu-miR-153-5p, mmu-miR-466q, mmu-miR-27b-5p, mmu-miR-1904, mmu-miR-6951-5p, mmu-miR-6981-3p, mmu-miR-194-5p, mmu-miR-322-3p, mmu-miR-3095-5p, mmu-miR-30a-3p, mmu-miR-6335, mmu-miR-6516-5p, mmu-miR-30e-3p, mmu-miR-7680-5p, mmu-miR-7a-1-3p, mmu-miR-700-5p, mmu-miR-693-3p, mmu-miR-7013-3p, mmu-miR-12205-5p, mmu-miR-92b-3p, mmu-miR-145b, mmu-miR-6963-5p, mmu-miR-100-3p, mmu-miR-384-3p, mmu-miR-6999-3p, mmu-miR-7656-5p, mmu-miR-216c-5p, mmu-miR-6540-3p |
| circRNA01543, | mmu-miR-7649-3p, mmu-miR-744-5p, mmu-miR-7036b-5p, mmu-miR-1963, mmu-miR-7032-3p, mmu-miR-7073-3p, mmu-miR-130b-5p, mmu-miR-7211-5p, mmu-miR-7009-3p, mmu-miR-6961-3p, mmu-miR-128-2-5p, mmu-miR-6921-3p, mmu-miR-204-5p, mmu-miR-211-5p, mmu-let-7i-3p, mmu-miR-7116-3p, mmu-miR-6947-3p, mmu-miR-3473c, mmu-miR-6963-3p, mmu-miR-9768-5p, mmu-miR-1893, mmu-miR-128-1-5p, mmu-miR-1947-5p, mmu-miR-7056-3p, mmu-miR-691, mmu-miR-1191b-3p, mmu-miR-31-5p, mmu-miR-1198-3p, mmu-miR-874-5p, mmu-miR-7660-5p |
| circRNA03390 | mmu-miR-669g, mmu-miR-6715-5p, mmu-miR-7221-3p, mmu-miR-6915-5p, mmu-miR-290a-3p, mmu-miR-6996-3p, mmu-miR-7033-5p, mmu-miR-7013-3p, mmu-miR-12196-3p, mmu-miR-6937-3p, mmu-miR-26a-5p, mmu-miR-3095-3p, mmu-miR-29b-2-5p, mmu-miR-743a-5p, mmu-miR-6987-5p, mmu-miR-367-5p, mmu-miR-6900-5p, mmu-miR-6900-3p, mmu-miR-709, mmu-miR-5619-5p, mmu-miR-7010-3p, mmu-miR-34a-3p, mmu-miR-12201-3p, mmu-miR-292a-3p, mmu-miR-26b-5p, mmu-miR-540-3p, mmu-miR-1929-5p, mmu-miR-871-5p, mmu-miR-7032-5p, mmu-miR-7218-5p |
| circRNA03847 | mmu-miR-693-3p, mmu-miR-717, mmu-miR-743b-5p, mmu-miR-465a-5p, mmu-miR-7670-5p, mmu-miR-465d-5p, mmu-miR-743a-5p, mmu-miR-6948-5p, mmu-miR-6405, mmu-miR-674-3p, mmu-miR-871-5p, mmu-miR-6962-5p, mmu-miR-300-5p, mmu-miR-29c-5p, mmu-miR-6352, mmu-miR-7668-5p, mmu-miR-141-5p, mmu-miR-669c-5p, mmu-miR-3154, mmu-miR-3083b-3p, mmu-miR-21b, mmu-miR-12205-3p, mmu-miR-5112, mmu-miR-7018-5p, mmu-miR-450a-1-3p, mmu-miR-1947-5p, mmu-miR-1224-5p, mmu-miR-465b-5p, mmu-miR-465c-5p, mmu-miR-6954-5p |
| circRNA00982 | mmu-miR-6951-5p, mmu-miR-7214-5p, mmu-miR-1192, mmu-miR-12200-5p, mmu-miR-489-5p, mmu-miR-6377, mmu-miR-6951-3p, mmu-miR-6344, mmu-miR-495-3p, mmu-miR-590-3p, mmu-miR-883b-5p, mmu-miR-223-5p, mmu-miR-183-3p, mmu-miR-103-1-5p, mmu-miR-103-2-5p, mmu-miR-107-5p, mmu-miR-6899-3p, mmu-miR-26b-5p, mmu-miR-7646-5p, mmu-miR-7116-3p, mmu-miR-7010-5p, mmu-miR-1958, mmu-miR-8092, mmu-miR-6912-3p, mmu-miR-219c-3p, mmu-miR-20a-5p, mmu-miR-539-3p, mmu-miR-8118, mmu-miR-7681-3p, mmu-miR-381-3p |
| circRNA02067, | mmu-miR-7029-3p, mmu-miR-6972-5p, mmu-miR-6900-5p, mmu-miR-677-3p, mmu-miR-1291, mmu-miR-582-5p, mmu-miR-6985-5p, mmu-miR-8094, mmu-miR-712-5p, mmu-miR-7018-3p, mmu-miR-6987-5p, mmu-miR-363-5p, mmu-miR-1843a-5p, mmu-miR-7222-3p, mmu-miR-6371, mmu-miR-674-5p, mmu-miR-7088-5p, mmu-miR-3095-3p, mmu-miR-6418-3p, mmu-miR-1264-5p, mmu-miR-5623-5p, mmu-miR-6958-3p, mmu-miR-7227-5p, mmu-miR-669e-5p, mmu-miR-7215-3p, mmu-miR-6995-3p, mmu-miR-145a-3p, mmu-miR-6924-5p, mmu-miR-12188-5p, mmu-miR-7015-3p |
| circRNA01985, | mmu-miR-12202-3p, mmu-miR-320-5p, mmu-miR-6946-3p, mmu-miR-7028-3p, mmu-miR-7030-3p, mmu-miR-298-3p, mmu-miR-6909-3p, mmu-miR-290b-5p, mmu-miR-9768-3p, mmu-miR-6901-3p, mmu-miR-382-3p, mmu-miR-7031-5p, mmu-miR-7242-5p, mmu-miR-6974-3p, mmu-miR-5615-5p, mmu-miR-6966-5p, mmu-miR-6957-3p, mmu-miR-551b-3p, mmu-miR-3091-3p, mmu-miR-338-3p, mmu-miR-873a-5p, mmu-miR-2861, mmu-miR-7657-3p, mmu-miR-134-5p, mmu-miR-7041-3p, mmu-miR-7093-3p, mmu-miR-3620-3p, mmu-miR-217-5p, mmu-miR-7030-5p, mmu-miR-6923-3p |
| circRNA00825 | mmu-miR-877-3p, mmu-miR-8119, mmu-miR-186-3p, mmu-miR-7012-5p, mmu-miR-6974-5p, mmu-miR-7030-5p, mmu-miR-7226-5p, mmu-miR-504-3p, mmu-miR-7057-5p, mmu-miR-6418-5p, mmu-miR-7058-5p, mmu-miR-7212-3p, mmu-miR-8113, mmu-miR-188-3p, mmu-miR-497b, mmu-miR-130a-5p, mmu-miR-1249-5p, mmu-miR-1904, mmu-miR-501-5p, mmu-miR-7018-5p, mmu-miR-5114, mmu-miR-7050-5p, mmu-miR-105, mmu-miR-3473a, mmu-miR-6539, mmu-miR-330-5p, mmu-miR-6769b-3p, mmu-miR-6403, mmu-miR-190b-3p, mmu-miR-669c-3p |
| circRNA03309 | mmu-miR-378d, mmu-miR-7050-5p, mmu-miR-185-3p, mmu-miR-301a-5p, mmu-miR-3113-3p, mmu-miR-7012-5p, mmu-miR-3095-5p, mmu-miR-6952-3p, mmu-miR-12189-3p, mmu-miR-149-5p, mmu-miR-6395, mmu-miR-7084-3p, mmu-miR-92a-2-5p, mmu-miR-6418-5p, mmu-miR-702-5p, mmu-miR-12192-5p, mmu-miR-328-3p, mmu-miR-6379, mmu-miR-874-3p, mmu-miR-6931-5p, mmu-miR-7026-3p, mmu-miR-6907-5p, mmu-miR-106a-3p, mmu-miR-5623-3p, mmu-miR-7035-3p, mmu-miR-700-5p, mmu-miR-6936-5p, mmu-miR-7649-5p, mmu-miR-24-3p, mmu-miR-150-3p |
| circRNA02418 | mmu-miR-7092-3p, mmu-miR-129b-5p, mmu-miR-7678-3p, mmu-miR-130a-5p, mmu-miR-6935-3p, mmu-miR-7036b-3p, mmu-miR-7116-3p, mmu-miR-6384, mmu-miR-6344, mmu-miR-12205-5p, mmu-miR-7680-3p, mmu-miR-6984-3p, mmu-miR-6999-3p, mmu-miR-12180-3p, mmu-miR-26a-2-3p, mmu-miR-203-5p, mmu-miR-3057-5p, mmu-miR-6964-3p, mmu-miR-7657-3p, mmu-miR-23b-5p, mmu-miR-5101, mmu-miR-7231-3p, mmu-miR-7663-5p, mmu-miR-672-5p, mmu-miR-670-3p, mmu-miR-691, mmu-miR-181a-1-3p, mmu-miR-6928-3p, mmu-miR-3099-5p, mmu-miR-434-3p |
| circRNA04488 | mmu-miR-7044-5p, mmu-miR-6982-5p, mmu-miR-6930-5p, mmu-miR-7028-5p, mmu-miR-667-5p, mmu-miR-7081-5p, mmu-miR-3075-3p, mmu-miR-7669-3p, mmu-miR-30c-1-3p, mmu-miR-3066-5p, mmu-miR-5623-5p, mmu-miR-449c-5p, mmu-miR-7019-5p, mmu-miR-485-5p, mmu-miR-449b, mmu-miR-1843a-5p, mmu-miR-5110, mmu-miR-7001-5p, mmu-miR-6360, mmu-miR-7242-3p, mmu-miR-7649-3p, mmu-miR-763, mmu-miR-679-5p, mmu-miR-1982-5p, mmu-miR-7092-3p, mmu-miR-6911-5p, mmu-miR-7061-5p, mmu-miR-6988-5p, mmu-miR-5114, mmu-miR-6929-5p |
| circRNA01605 | mmu-miR-7218-5p, mmu-miR-7652-3p, mmu-miR-6897-5p, mmu-miR-682, mmu-miR-146b-5p, mmu-miR-106a-3p, mmu-miR-328-5p, mmu-miR-383-3p, mmu-miR-146a-5p, mmu-miR-20b-3p, mmu-miR-664-5p, mmu-miR-3076-3p, mmu-miR-7240-5p, mmu-miR-185-5p, mmu-miR-6984-5p, mmu-miR-12188-3p, mmu-miR-7222-5p, mmu-miR-191-3p, mmu-miR-12202-3p, mmu-miR-29b-1-5p, mmu-miR-882, mmu-miR-488-3p, mmu-miR-3110-3p, mmu-miR-7667-5p, mmu-miR-376b-3p, mmu-miR-687, mmu-miR-6396, mmu-miR-194-2-3p, mmu-miR-383-5p, mmu-miR-673-5p |
| circRNA03073 | mmu-miR-7229-3p, mmu-miR-7058-5p, mmu-miR-672-3p, mmu-miR-7033-5p, mmu-miR-7226-5p, mmu-miR-881-5p, mmu-miR-149-3p, mmu-miR-669c-3p, mmu-miR-185-5p, mmu-miR-7661-5p, mmu-miR-466f-3p, mmu-miR-6373, mmu-miR-6969-5p, mmu-miR-7094-1-5p, mmu-miR-6418-5p, mmu-miR-504-3p, mmu-miR-509-5p, mmu-miR-3473a, mmu-miR-142a-5p, mmu-miR-7118-5p, mmu-miR-1264-3p, mmu-miR-6925-5p, mmu-miR-7222-5p, mmu-miR-188-3p, mmu-miR-5110, mmu-miR-466q, mmu-miR-6971-5p, mmu-miR-145a-5p, mmu-miR-3097-5p, mmu-miR-3057-3p |
| circRNA02313 | mmu-miR-7652-3p, mmu-miR-6898-3p, mmu-miR-1933-3p, mmu-miR-298-5p, mmu-miR-7649-3p, mmu-miR-710, mmu-miR-7086-5p, mmu-miR-1199-5p, mmu-miR-1956, mmu-miR-181b-5p, mmu-miR-1903, mmu-miR-3063-3p, mmu-miR-7015-5p, mmu-miR-3473c, mmu-miR-6931-5p, mmu-miR-8092, mmu-miR-7087-3p, mmu-miR-378b, mmu-miR-181d-5p, mmu-miR-7688-3p, mmu-miR-5113, mmu-miR-6398, mmu-miR-7230-5p, mmu-miR-465a-3p, mmu-miR-666-3p, mmu-miR-465b-3p, mmu-miR-465c-3p, mmu-miR-7238-3p, mmu-miR-6965-3p, mmu-miR-5132-5p |
| circRNA00723 | mmu-miR-7016-5p, mmu-miR-466k, mmu-miR-466i-5p, mmu-miR-5623-5p, mmu-miR-6939-5p, mmu-miR-7058-5p, mmu-miR-466d-5p, mmu-miR-669d-5p, mmu-miR-466f, mmu-miR-466l-5p, mmu-miR-3084-5p, mmu-miR-466n-5p, mmu-miR-6986-5p, mmu-miR-709, mmu-miR-451b, mmu-miR-7678-5p, mmu-miR-130b-3p, mmu-miR-669f-5p, mmu-miR-669n, mmu-miR-6921-5p, mmu-miR-7072-5p, mmu-miR-669a-5p, mmu-miR-1187, mmu-miR-669p-5p, mmu-miR-6953-5p, mmu-miR-7063-5p, mmu-miR-466a-5p, mmu-miR-466e-5p, mmu-miR-6922-5p, mmu-miR-767 |
| circRNA00447 | mmu-miR-6948-3p, mmu-miR-28a-3p, mmu-miR-7092-3p, mmu-miR-6917-5p, mmu-miR-8094, mmu-miR-134-5p, mmu-miR-667-5p, mmu-miR-712-5p, mmu-miR-193b-5p, mmu-miR-30c-1-3p, mmu-miR-708-3p, mmu-miR-144-5p, mmu-miR-7021-5p, mmu-miR-27b-5p, mmu-miR-7043-5p, mmu-miR-1942, mmu-miR-669h-5p, mmu-miR-7211-3p, mmu-miR-8108, mmu-miR-3105-3p, mmu-miR-369-5p, mmu-miR-6373, mmu-miR-6992-5p, mmu-miR-8093, mmu-miR-219a-1-3p, mmu-miR-181b-2-3p, mmu-miR-6408, mmu-miR-6415, mmu-miR-6975-5p, mmu-miR-6237 |
| circRNA03673 | mmu-miR-1952, mmu-miR-421-3p, mmu-miR-7655-5p, mmu-miR-185-3p, mmu-miR-883b-5p, mmu-miR-494-5p, mmu-miR-3071-3p, mmu-miR-3062-5p, mmu-miR-7658-3p, mmu-miR-6945-5p, mmu-miR-7077-5p, mmu-miR-667-5p, mmu-miR-7686-5p, mmu-miR-7222-5p, mmu-miR-673-3p, mmu-miR-679-3p, mmu-miR-124b-3p, mmu-miR-148b-3p, mmu-miR-410-5p, mmu-miR-3110-3p, mmu-miR-154-5p, mmu-miR-470-5p, mmu-miR-3099-5p, mmu-miR-1188-5p, mmu-miR-6403, mmu-miR-8112, mmu-miR-1894-5p, mmu-miR-3971, mmu-miR-6982-3p, mmu-miR-669h-5p |
| circRNA04655 | mmu-miR-669a-5p, mmu-miR-669p-5p, mmu-miR-12205-5p, mmu-miR-5627-3p, mmu-miR-8108, mmu-miR-3064-5p, mmu-miR-7054-5p, mmu-miR-196b-5p, mmu-miR-693-5p, mmu-miR-669f-5p, mmu-miR-669o-5p, mmu-miR-6393, mmu-miR-6400, mmu-miR-7070-3p, mmu-miR-7659-3p, mmu-miR-196a-5p, mmu-miR-743a-5p, mmu-miR-669l-5p, mmu-miR-140-5p, mmu-miR-672-3p, mmu-miR-3098-3p, mmu-miR-323-3p, mmu-miR-1231-3p, mmu-miR-7053-3p, mmu-miR-3095-5p, mmu-miR-132-3p, mmu-miR-22-5p, mmu-miR-7021-3p, mmu-let-7f-5p, mmu-miR-500-5p |
| circRNA00747 | mmu-miR-1958, mmu-miR-7092-3p, mmu-miR-6967-3p, mmu-miR-6380, mmu-miR-19b-2-5p, mmu-miR-211-5p, mmu-miR-6240, mmu-miR-6951-5p, mmu-miR-7012-5p, mmu-miR-1950, mmu-miR-6964-3p, mmu-miR-669b-5p, mmu-miR-26a-2-3p, mmu-miR-106a-5p, mmu-miR-153-5p, mmu-miR-7661-5p, mmu-miR-677-3p, mmu-miR-204-5p, mmu-miR-6341, mmu-miR-7061-3p, mmu-miR-136-5p, mmu-miR-7651-3p, mmu-miR-5619-5p, mmu-miR-686, mmu-miR-292b-5p, mmu-miR-7679-5p, mmu-miR-106b-5p, mmu-miR-301b-3p, mmu-miR-6899-3p, mmu-miR-294-5p |
| circRNA03674 | mmu-miR-9768-3p, mmu-miR-7077-5p, mmu-miR-7027-5p, mmu-miR-1952, mmu-miR-421-3p, mmu-miR-6934-5p, mmu-miR-7226-5p, mmu-miR-124b-3p, mmu-miR-7655-5p, mmu-miR-185-3p, mmu-miR-883b-5p, mmu-miR-7010-5p, mmu-miR-494-5p, mmu-miR-1188-5p, mmu-miR-3071-3p, mmu-miR-3062-5p, mmu-miR-653-3p, mmu-miR-7054-5p, mmu-miR-7646-3p, mmu-miR-7658-3p, mmu-miR-7683-3p, mmu-miR-6945-5p, mmu-miR-7047-5p, mmu-miR-667-5p, mmu-miR-7686-5p, mmu-miR-6901-3p, mmu-miR-8106, mmu-miR-7046-5p, mmu-miR-188-3p, mmu-miR-6983-5p |
| circRNA03831 | mmu-miR-7092-3p, mmu-miR-7651-3p, mmu-miR-30d-3p, mmu-miR-30e-3p, mmu-miR-30a-3p, mmu-miR-3084-3p, mmu-miR-17-3p, mmu-miR-6899-3p, mmu-miR-6935-3p, mmu-miR-106a-3p, mmu-miR-7238-3p, mmu-miR-20b-3p, mmu-miR-666-3p, mmu-miR-222-5p, mmu-miR-7650-3p, mmu-miR-29b-3p, mmu-miR-29c-3p, mmu-miR-6999-3p, mmu-miR-760-3p, mmu-miR-146a-3p, mmu-miR-742-5p, mmu-miR-466o-3p, mmu-miR-29a-3p, mmu-miR-7214-5p, mmu-miR-320-3p, mmu-miR-6928-3p, mmu-miR-7038-5p, mmu-miR-3112-3p, mmu-miR-3572-3p, mmu-miR-410-3p |
| circRNA06452 | mmu-miR-3473c, mmu-miR-7013-5p, mmu-miR-7649-3p, mmu-miR-6539, mmu-miR-1960, mmu-miR-1a-2-5p, mmu-miR-12187-3p, mmu-miR-1a-1-5p, mmu-miR-133c, mmu-miR-691, mmu-miR-130a-5p, mmu-miR-6537-5p, mmu-miR-133a-3p, mmu-miR-133b-3p, mmu-miR-12184-3p, mmu-miR-7041-5p, mmu-miR-669e-5p, mmu-miR-6946-3p, mmu-miR-693-5p, mmu-miR-7663-5p, mmu-miR-5129-3p, mmu-miR-6975-3p, mmu-miR-499-5p, mmu-miR-6945-5p, mmu-miR-590-5p, mmu-miR-154-3p, mmu-miR-3106-5p, mmu-miR-3971, mmu-miR-7063-3p, mmu-miR-6919-3p |
| circRNA08673 | mmu-miR-7021-3p, mmu-miR-7030-3p, mmu-miR-6987-3p, mmu-miR-7008-3p, mmu-miR-7077-3p, mmu-miR-6965-3p, mmu-miR-6973b-3p, mmu-miR-6976-3p, mmu-miR-7039-3p, mmu-miR-6966-3p, mmu-miR-7048-3p, mmu-miR-7002-3p, mmu-miR-421-3p, mmu-miR-9768-3p, mmu-miR-7063-3p, mmu-miR-7077-5p, mmu-miR-12191-3p, mmu-miR-7092-3p, mmu-miR-3104-3p, mmu-miR-207, mmu-miR-7027-5p, mmu-miR-1952, mmu-miR-6934-5p, mmu-miR-532-3p, mmu-miR-7226-5p, mmu-miR-124b-3p, mmu-miR-7686-5p, mmu-miR-7655-5p, mmu-miR-185-3p, mmu-miR-211-3p |
| circRNA06661 | mmu-miR-335-3p, mmu-miR-147-5p, mmu-miR-7227-5p, mmu-miR-6539, mmu-miR-6926-5p, mmu-miR-200a-3p, mmu-miR-7674-3p, mmu-miR-6907-5p, mmu-miR-296-3p, mmu-miR-12187-3p, mmu-miR-468-5p, mmu-miR-568, mmu-miR-3967, mmu-miR-194-1-3p, mmu-miR-450a-1-3p, mmu-miR-369-3p, mmu-miR-7b-5p, mmu-miR-141-3p, mmu-miR-208b-5p, mmu-miR-23b-3p, mmu-miR-409-5p, mmu-miR-466f-3p, mmu-miR-466i-3p, mmu-miR-216a-3p, mmu-miR-374c-5p, mmu-miR-216b-3p, mmu-miR-6405, mmu-miR-7065-5p, mmu-miR-450b-3p, mmu-miR-330-5p |
| circRNA08440 | mmu-miR-667-5p, mmu-miR-138-5p, mmu-miR-17-3p, mmu-miR-7216-5p, mmu-miR-335-3p, mmu-miR-5622-3p, mmu-miR-3097-5p, mmu-miR-7005-3p, mmu-miR-8104, mmu-miR-203-5p, mmu-miR-5620-5p, mmu-miR-382-5p, mmu-miR-5101, mmu-miR-466b-5p, mmu-miR-466o-5p, mmu-miR-7088-5p, mmu-miR-326-5p, mmu-miR-3971, mmu-miR-7234-5p, mmu-miR-7087-5p, mmu-miR-3109-3p, mmu-miR-12202-5p, mmu-miR-665-3p, mmu-miR-7649-3p, mmu-miR-7237-5p, mmu-miR-666-3p, mmu-miR-6933-3p, mmu-miR-124-5p, mmu-miR-466c-5p, mmu-miR-1b-3p |
| circRNA09919 | mmu-miR-1982-3p, mmu-miR-7054-3p, mmu-miR-7026-3p, mmu-miR-6958-3p, mmu-miR-741-3p, mmu-miR-3074-5p, mmu-miR-181b-2-3p, mmu-miR-501-5p, mmu-miR-1188-5p, mmu-miR-652-3p, mmu-miR-30d-3p, mmu-miR-377-3p, mmu-miR-6998-3p, mmu-miR-489-5p, mmu-miR-7228-3p, mmu-miR-7667-3p, mmu-miR-7226-5p, mmu-miR-7648-5p, mmu-miR-760-3p, mmu-miR-6911-3p, mmu-miR-7065-3p, mmu-miR-7678-3p, mmu-miR-879-5p, mmu-miR-7037-5p, mmu-miR-30a-3p, mmu-miR-181b-1-3p, mmu-miR-7068-5p, mmu-miR-7659-5p, mmu-miR-1291, mmu-miR-143-3p |

**Supplementary** **Table 3 Differentially expressed miRNAs in the hippocampus of 5×FAD mice.**

| **miRNA** | **log2FoldChange** | ***p-*value** |
| --- | --- | --- |
| mmu-let-7a-5p | -0.02411869 | 0.897681291 |
| mmu-let-7a-1-3p | -0.307772568 | 0.073148179 |
| mmu-let-7c-2-3p | -0.307772568 | 0.073148179 |
| mmu-let-7a-2-3p | -0.713427012 | 0.117024125 |
| mmu-let-7b-5p | 0.218230701 | 0.321187193 |
| mmu-let-7b-3p | 0.098780781 | 0.597827129 |
| mmu-let-7c-5p | 0.118896944 | 0.572286362 |
| mmu-let-7c-1-3p | 0.118740288 | 0.738404653 |
| mmu-let-7d-5p | -0.017876265 | 0.926729945 |
| mmu-let-7d-3p | 0.002441203 | 0.987742004 |
| mmu-let-7e-5p | -0.022475005 | 0.914017582 |
| mmu-let-7e-3p | 0.04614275 | 0.824747126 |
| mmu-let-7f-5p | -0.172203878 | 0.346852998 |
| mmu-let-7f-1-3p | -0.089561903 | 0.718874809 |
| mmu-novel-5 | -0.162144842 | 0.376189223 |
| mmu-let-7f-2-3p | -0.308925149 | 0.25356087 |
| mmu-let-7g-5p | -0.118555879 | 0.5379304 |
| mmu-let-7g-3p | 0.076648538 | 0.889487522 |
| mmu-let-7i-5p | -0.159509794 | 0.427309189 |
| mmu-let-7i-3p | 0.016833078 | 0.941441486 |
| mmu-let-7j | -0.352220189 | 0.066125062 |
| mmu-let-7k | 0.592482882 | 0.237099419 |
| mmu-miR-100-5p | -0.226519413 | 0.438023189 |
| mmu-miR-100-3p | 0.503717065 | 0.443539034 |
| mmu-miR-101a-5p | -0.125752669 | 0.742246361 |
| mmu-miR-101a-3p | 0.017048556 | 0.927068099 |
| mmu-miR-101c | 0.017046264 | 0.927100714 |
| mmu-miR-101b-5p | -0.475143652 | 0.901530955 |
| mmu-miR-101b-3p | 0.132246614 | 0.632562145 |
| mmu-miR-103-1-5p | 1.380395091 | 0.715139819 |
| mmu-miR-103-3p | -0.133760001 | 0.573143529 |
| mmu-miR-103-2-5p | 0.226110749 | 0.872592424 |
| mmu-miR-106a-5p | -0.031252416 | 0.954118426 |
| mmu-miR-106b-5p | 0.409464475 | 0.12191651 |
| mmu-miR-106b-3p | 0.276184727 | 0.139184015 |
| mmu-miR-107-5p | -0.050950259 | 0.969158388 |
| mmu-miR-107-3p | 0.032742808 | 0.871280439 |
| mmu-miR-10a-5p | 0.327396698 | 0.200650084 |
| mmu-miR-10a-3p | 0.781128165 | 0.487989614 |
| mmu-miR-10b-5p | 0.194166141 | 0.568511285 |
| mmu-miR-10b-3p | 0.938702528 | 0.505720935 |
| mmu-miR-1187 | 2.00600096 | 0.309547176 |
| mmu-miR-1188-5p | 0.395566078 | 0.619184876 |
| mmu-miR-1188-3p | -0.381619012 | 0.796713067 |
| mmu-miR-1191a | -0.045457854 | 0.900720751 |
| mmu-miR-1191b-5p | -0.524378104 | 0.647380086 |
| mmu-miR-1191b-3p | 0.418839528 | 0.880395082 |
| mmu-miR-1192 | 0.486628736 | 0.905071015 |
| mmu-miR-1193-5p | -0.235664336 | 0.831545851 |
| mmu-miR-1193-3p | -0.419183101 | 0.063070373 |
| mmu-miR-1194 | -0.161186307 | 0.748188617 |
| mmu-miR-1195 | 0.869604617 | 0.105484352 |
| mmu-miR-1197-3p | -0.2895305 | 0.395121597 |
| mmu-miR-1198-5p | -0.069443612 | 0.773739541 |
| mmu-miR-1198-3p | 0.276003115 | 0.612931668 |
| mmu-miR-122-5p | 0.246263017 | 0.645805833 |
| mmu-miR-1224-5p | 0.313206402 | 0.410211199 |
| mmu-miR-1224-3p | 1.225123778 | 0.476497014 |
| mmu-novel-107 | 0.325055335 | 0.388172941 |
| mmu-miR-1231-5p | 1.993966667 | 0.537058569 |
| mmu-miR-1231-3p | -1.086761746 | 0.641906324 |
| mmu-miR-124-5p | 0.307374849 | 0.27315457 |
| mmu-miR-124-3p | 0.332832994 | 0.236501565 |
| mmu-miR-1247-5p | 0.174116713 | 0.726871372 |
| mmu-miR-1247-3p | 0.548520204 | 0.772467349 |
| mmu-miR-1249-5p | -0.475143254 | 0.891326251 |
| mmu-miR-1249-3p | 0.105860395 | 0.650068613 |
| mmu-miR-1251-5p | 0.385716647 | 0.512007684 |
| mmu-miR-1251-3p | -0.47514326 | 0.89150293 |
| mmu-miR-1258-5p | 0.486628736 | 0.905071015 |
| mmu-miR-1258-3p | 2.965030658 | 0.257476717 |
| mmu-miR-125a-5p | -0.237779239 | 0.278292736 |
| mmu-miR-125a-3p | 0.415823054 | 0.177951079 |
| mmu-novel-263 | -0.190843346 | 0.381647871 |
| mmu-miR-125b-5p | -0.318254468 | 0.213988104 |
| mmu-miR-125b-1-3p | 0.216636456 | 0.390097173 |
| mmu-miR-125b-2-3p | -0.221973887 | 0.221056633 |
| mmu-miR-1264-5p | 0.225824004 | 0.784671591 |
| mmu-miR-1264-3p | 0.051951368 | 0.948553046 |
| mmu-miR-126a-5p | 0.104733536 | 0.631029016 |
| mmu-miR-126a-3p | -0.117940967 | 0.534283788 |
| mmu-miR-126b-3p | 1.005957869 | 0.668296347 |
| mmu-miR-127-5p | 0.058677234 | 0.848594784 |
| mmu-miR-127-3p | -0.067404285 | 0.79080282 |
| mmu-miR-128-1-5p | -0.125491861 | 0.610645561 |
| mmu-miR-128-3p | -0.017712261 | 0.915758877 |
| mmu-miR-128-2-5p | -0.099120846 | 0.951317542 |
| mmu-miR-129-5p | 0.029718436 | 0.906078875 |
| mmu-miR-129-1-3p | -0.53114321 | 0.0134574 |
| mmu-miR-129-2-3p | -0.60794037 | 0.019740798 |
| mmu-miR-1291 | -1.436924616 | 0.724728327 |
| mmu-miR-1298-5p | 0.362577145 | 0.743512998 |
| mmu-miR-1298-3p | -0.145164632 | 0.70946898 |
| mmu-miR-129b-3p | -2.516040207 | 0.498035283 |
| mmu-miR-1306-5p | -1.07498576 | 0.618315209 |
| mmu-miR-1306-3p | -1.436924616 | 0.724728327 |
| mmu-miR-130a-5p | 0.376141617 | 0.653744044 |
| mmu-miR-130a-3p | 0.510566475 | 0.034751705 |
| mmu-miR-130b-5p | 0.502355758 | 0.13063421 |
| mmu-miR-130b-3p | 0.673478758 | 0.205477491 |
| mmu-miR-132-5p | 0.189084064 | 0.422141961 |
| mmu-miR-132-3p | 0.127851539 | 0.48348635 |
| mmu-miR-133a-5p | -0.966959615 | 0.181259128 |
| mmu-miR-133a-3p | -0.178791047 | 0.497149725 |
| mmu-miR-133b-3p | 0.126299143 | 0.684012081 |
| mmu-miR-134-5p | 0.365474327 | 0.154534108 |
| mmu-miR-134-3p | 0.264929249 | 0.33129721 |
| mmu-miR-135a-5p | -0.478385388 | 0.021578533 |
| mmu-miR-135a-1-3p | 1.331840859 | 0.122146042 |
| mmu-miR-135a-2-3p | 0.002973482 | 0.99407419 |
| mmu-miR-135b-5p | 0.593031436 | 0.086185532 |
| mmu-miR-135b-3p | 0.262024978 | 0.746767624 |
| mmu-miR-136-5p | 0.204060273 | 0.470827778 |
| mmu-miR-136-3p | -0.068084521 | 0.735612418 |
| mmu-miR-137-5p | 0.454229832 | 0.321657292 |
| mmu-miR-137-3p | -0.171604327 | 0.485292694 |
| mmu-miR-138-5p | 0.102126549 | 0.618435647 |
| mmu-miR-138-1-3p | 0.361080973 | 0.273707008 |
| mmu-miR-138-2-3p | 0.130255514 | 0.668779728 |
| mmu-miR-139-5p | -0.087762031 | 0.733258703 |
| mmu-miR-139-3p | -0.029739955 | 0.916262333 |
| mmu-miR-140-5p | -0.173385397 | 0.442072466 |
| mmu-miR-140-3p | 0.015808616 | 0.927170548 |
| mmu-miR-141-5p | 7.257811034 | 1 |
| mmu-miR-141-3p | 5.655714613 | 1 |
| mmu-miR-142a-5p | 0.844397582 | 0.011642981 |
| mmu-miR-142a-3p | 0.763674383 | 0.029690809 |
| mmu-miR-142b | 3.181952552 | 0.190571182 |
| mmu-miR-143-5p | 0.081635146 | 0.700488751 |
| mmu-miR-143-3p | 0.242717813 | 0.295151909 |
| mmu-miR-144-5p | -0.405946576 | 0.201171258 |
| mmu-miR-144-3p | 0.214959282 | 0.554047114 |
| mmu-miR-145a-5p | -0.570701918 | 0.005348058 |
| mmu-miR-145a-3p | 0.117024367 | 0.575426761 |
| mmu-miR-145b | -0.065856734 | 0.931742563 |
| mmu-miR-146a-5p | 0.714854503 | 0.102741325 |
| mmu-miR-146a-3p | 0.161570465 | 0.836889535 |
| mmu-miR-146b-5p | -0.175304248 | 0.440030034 |
| mmu-miR-146b-3p | 0.342827565 | 0.425208036 |
| mmu-miR-148a-5p | 0.296549389 | 0.410283976 |
| mmu-miR-148a-3p | -0.112055205 | 0.665879467 |
| mmu-miR-148b-5p | -0.181013149 | 0.478999579 |
| mmu-miR-148b-3p | -0.280540206 | 0.186072689 |
| mmu-miR-149-5p | 0.050171373 | 0.806936127 |
| mmu-miR-150-5p | 0.183725017 | 0.298568085 |
| mmu-miR-150-3p | -0.343898291 | 0.502102487 |
| mmu-miR-151-5p | 0.09723874 | 0.677623363 |
| mmu-miR-151-3p | 0.060980746 | 0.801622489 |
| mmu-miR-152-5p | -0.052920033 | 0.864500386 |
| mmu-miR-152-3p | 0.191493309 | 0.244856811 |
| mmu-miR-153-5p | 1.679319373 | 0.101291555 |
| mmu-miR-153-3p | -0.203669666 | 0.408438462 |
| mmu-miR-154-5p | 0.084614882 | 0.739347934 |
| mmu-miR-154-3p | 0.169022448 | 0.370692524 |
| mmu-miR-155-5p | 0.533129112 | 0.25323028 |
| mmu-miR-155-3p | -0.390285972 | 0.879702321 |
| mmu-miR-15a-5p | 0.246366585 | 0.321196313 |
| mmu-miR-15a-3p | 0.142657671 | 0.625917865 |
| mmu-miR-15b-5p | -0.102718188 | 0.746580468 |
| mmu-miR-15b-3p | -0.042919014 | 0.891734815 |
| mmu-miR-16-5p | 0.025629613 | 0.927593816 |
| mmu-miR-16-1-3p | 0.08155131 | 0.854875214 |
| mmu-miR-16-2-3p | 0.142822505 | 0.766614837 |
| mmu-miR-1668 | -1.436924616 | 0.724728327 |
| mmu-miR-17-5p | 0.195283313 | 0.45344723 |
| mmu-miR-17-3p | 0.208656152 | 0.561528218 |
| mmu-miR-181a-5p | 0.180540463 | 0.483520702 |
| mmu-miR-181a-1-3p | 0.033703987 | 0.876012331 |
| mmu-miR-181a-2-3p | 0.964444329 | 0.009372984 |
| mmu-miR-181b-5p | -0.042969627 | 0.790438371 |
| mmu-miR-181b-1-3p | 0.380209759 | 0.198739733 |
| mmu-miR-181b-2-3p | 2.168460796 | 0.160743019 |
| mmu-miR-181c-5p | 0.438664384 | 0.068364625 |
| mmu-miR-181c-3p | 0.181933297 | 0.361244954 |
| mmu-miR-181d-5p | 0.172479651 | 0.411949321 |
| mmu-miR-181d-3p | 0.333198307 | 0.596286089 |
| mmu-miR-182-5p | 5.428187599 | 1 |
| mmu-miR-182-3p | 4.982657754 | 1 |
| mmu-miR-183-5p | 5.625652035 | 1 |
| mmu-miR-183-3p | 5.405121944 | 1 |
| mmu-miR-1839-5p | -0.270455064 | 0.436842596 |
| mmu-miR-1839-3p | -1.687664178 | 0.034450635 |
| mmu-miR-184-3p | -0.268939921 | 0.52886074 |
| mmu-miR-1843a-5p | -0.438216554 | 0.052563234 |
| mmu-miR-1843a-3p | -0.613533838 | 0.467253874 |
| mmu-miR-1843b-5p | -0.434886351 | 0.054717646 |
| mmu-miR-1843b-3p | 0.34259547 | 0.749931523 |
| mmu-miR-185-5p | -0.50251416 | 0.019657575 |
| mmu-miR-185-3p | 0.651108733 | 0.1304401 |
| mmu-miR-186-5p | -0.537171562 | 0.058677151 |
| mmu-miR-186-3p | -0.315879359 | 0.598675324 |
| mmu-miR-187-5p | -0.124215617 | 0.688577078 |
| mmu-miR-187-3p | 0.433918412 | 0.029485175 |
| mmu-miR-188-5p | 0.439033298 | 0.320460594 |
| mmu-miR-188-3p | 0.226461148 | 0.559458655 |
| mmu-miR-1895 | 0.508833842 | 0.704385828 |
| mmu-miR-18a-5p | 0.106185467 | 0.834958893 |
| mmu-miR-18a-3p | 0.335884189 | 0.767503108 |
| mmu-miR-18b-3p | -1.436924616 | 0.724728327 |
| mmu-miR-190a-5p | -0.03407412 | 0.910505333 |
| mmu-miR-190a-3p | -0.487095648 | 0.166180394 |
| mmu-miR-190b-5p | 0.114021454 | 0.79992415 |
| mmu-miR-190b-3p | -0.020632833 | 0.992347881 |
| mmu-miR-191-5p | -0.319462232 | 0.263458344 |
| mmu-miR-191-3p | 0.088997555 | 0.7438111 |
| mmu-miR-1912-5p | 0.803792257 | 0.430677307 |
| mmu-miR-1912-3p | -0.187817015 | 0.863738174 |
| mmu-miR-192-5p | -0.315212169 | 0.145278036 |
| mmu-miR-192-3p | 0.246381218 | 0.884158091 |
| mmu-miR-1927 | -0.167022631 | 0.837484126 |
| mmu-miR-1929-5p | -0.088481811 | 0.963558765 |
| mmu-miR-1929-3p | 0.486628736 | 0.905071015 |
| mmu-miR-1930-5p | 0.055263894 | 0.925249842 |
| mmu-miR-1930-3p | 1.388015401 | 0.730997715 |
| mmu-miR-1931 | -1.295866507 | 0.64311074 |
| mmu-miR-1932 | 0.486628736 | 0.905071015 |
| mmu-miR-1933-3p | -1.214233548 | 0.361792939 |
| mmu-miR-1934-5p | 0.627153769 | 0.514224623 |
| mmu-miR-1934-3p | -0.687685199 | 0.805514012 |
| mmu-miR-1936 | -2.886501946 | 0.134393765 |
| mmu-miR-193a-5p | -0.026969981 | 0.962729437 |
| mmu-miR-193a-3p | 1.143315122 | 0.42225607 |
| mmu-miR-193b-5p | 0.171386933 | 0.85658753 |
| mmu-miR-193b-3p | 0.123258086 | 0.697797513 |
| mmu-miR-194-5p | -0.033425019 | 0.870419166 |
| mmu-miR-194-2-3p | 1.396468455 | 0.194415976 |
| mmu-miR-1941-5p | 0.486628736 | 0.905071015 |
| mmu-miR-1941-3p | -0.417045436 | 0.806975101 |
| mmu-miR-1943-5p | -0.388390538 | 0.498616612 |
| mmu-miR-1943-3p | 0.12593231 | 0.940131448 |
| mmu-miR-1945 | -1.67755237 | 0.278964432 |
| mmu-miR-1946a | 0.418835885 | 0.880827786 |
| mmu-miR-1946b | -0.709335956 | 0.799672513 |
| mmu-miR-1947-5p | 0.696694119 | 0.193049039 |
| mmu-miR-1947-3p | 0.200145737 | 0.927680404 |
| mmu-miR-1948-5p | -0.537039637 | 0.403783131 |
| mmu-miR-1948-3p | -0.156476492 | 0.842430029 |
| mmu-miR-1949 | 1.74285558 | 0.172464105 |
| mmu-miR-1951 | 0.486628736 | 0.905071015 |
| mmu-miR-1954 | 0.486628736 | 0.905071015 |
| mmu-miR-1955-5p | -0.513938214 | 0.333392946 |
| mmu-miR-1955-3p | -0.106461239 | 0.935258757 |
| mmu-miR-1957a | 0.94123205 | 0.251343941 |
| mmu-miR-195a-5p | 0.209435214 | 0.392755255 |
| mmu-miR-195a-3p | 0.103305214 | 0.748705944 |
| mmu-miR-195b | 0.309677214 | 0.814728895 |
| mmu-miR-1960 | 0.537198106 | 0.747296195 |
| mmu-miR-1961 | 0.884942442 | 0.637243567 |
| mmu-miR-1963 | 0.486628736 | 0.905071015 |
| mmu-miR-1964-5p | -1.436924616 | 0.724728327 |
| mmu-miR-1964-3p | -1.35741419 | 0.037749015 |
| mmu-miR-1966-5p | 0.486628736 | 0.905071015 |
| mmu-miR-1966-3p | 0.742702754 | 0.566614498 |
| mmu-miR-1968-5p | -0.521413446 | 0.430954795 |
| mmu-miR-1968-3p | -0.475143652 | 0.901530955 |
| mmu-miR-1969 | -2.566408499 | 0.008142318 |
| mmu-miR-196a-1-3p | 0.486628736 | 0.905071015 |
| mmu-miR-1971 | -1.436924616 | 0.724728327 |
| mmu-miR-1981-5p | -0.161487429 | 0.465379885 |
| mmu-miR-1981-3p | -0.276472578 | 0.311891155 |
| mmu-miR-1982-3p | 0.166206319 | 0.757010263 |
| mmu-miR-1983 | -0.457796969 | 0.084248486 |
| mmu-miR-199a-5p | 0.347683917 | 0.179333096 |
| mmu-miR-199a-3p | 0.435218667 | 0.018240207 |
| mmu-miR-199b-3p | 0.435218667 | 0.018240207 |
| mmu-miR-199b-5p | 0.268421334 | 0.4496114 |
| mmu-miR-19a-3p | -0.525796267 | 0.091905551 |
| mmu-miR-19b-3p | -0.469109895 | 0.106127003 |
| mmu-miR-19b-1-5p | 0.996324778 | 0.709463373 |
| mmu-miR-1a-1-5p | -2.168503822 | 0.423042744 |
| mmu-miR-1a-3p | -0.280353287 | 0.265071339 |
| mmu-miR-1b-5p | -0.417045436 | 0.806975101 |
| mmu-miR-1b-3p | 0.291138464 | 0.834567264 |
| mmu-miR-200a-5p | 4.392060663 | 1 |
| mmu-miR-200a-3p | 5.477753084 | 1 |
| mmu-miR-200b-5p | 4.811214422 | 1 |
| mmu-miR-200b-3p | 4.759931848 | 1 |
| mmu-miR-200c-5p | 3.006744563 | 0.316167212 |
| mmu-miR-200c-3p | 5.278983496 | 1 |
| mmu-miR-201-5p | -0.383503133 | 0.673339533 |
| mmu-miR-201-3p | -1.436924616 | 0.724728327 |
| mmu-miR-202-5p | 0.025643383 | 0.972460895 |
| mmu-miR-202-3p | 0.486628736 | 0.905071015 |
| mmu-miR-203-5p | -1.436924616 | 0.724728327 |
| mmu-miR-203-3p | 0.575743777 | 0.026009821 |
| mmu-miR-204-5p | 0.309692514 | 0.463946116 |
| mmu-miR-204-3p | 0.918842905 | 0.088378651 |
| mmu-miR-205-5p | 0.996713288 | 0.065347084 |
| mmu-miR-206-3p | 0.286136749 | 0.529018141 |
| mmu-miR-208a-3p | 0.486628736 | 0.905071015 |
| mmu-miR-208b-3p | 0.524535914 | 0.729548267 |
| mmu-miR-20a-5p | -0.043685484 | 0.852367652 |
| mmu-miR-20a-3p | -0.152917711 | 0.627197071 |
| mmu-miR-20b-5p | 0.514114464 | 0.228562251 |
| mmu-miR-20b-3p | 0.486628736 | 0.905071015 |
| mmu-miR-210-5p | -0.07773605 | 0.913934614 |
| mmu-miR-210-3p | 0.165189865 | 0.587502968 |
| mmu-miR-211-5p | 0.593716236 | 0.147582935 |
| mmu-miR-212-5p | -0.383843651 | 0.12485164 |
| mmu-miR-212-3p | 0.016882729 | 0.923894187 |
| mmu-novel-101 | -0.179275607 | 0.483448337 |
| mmu-miR-2137 | 2.372815347 | 0.359917735 |
| mmu-miR-214-5p | 1.637170658 | 0.291103179 |
| mmu-miR-214-3p | 0.681231292 | 0.208765719 |
| mmu-miR-215-5p | -0.302591343 | 0.570069883 |
| mmu-miR-216a-5p | 0.788793879 | 0.420575114 |
| mmu-miR-216a-3p | -1.496763651 | 0.303770815 |
| mmu-miR-216b-5p | -0.08415691 | 0.911264691 |
| mmu-miR-216b-3p | 0.892582311 | 0.629081951 |
| mmu-miR-216c-5p | 0.486628736 | 0.905071015 |
| mmu-miR-217-5p | -0.240367707 | 0.73453649 |
| mmu-miR-217-3p | -1.011321289 | 0.629166904 |
| mmu-miR-218-5p | 0.019449861 | 0.932980375 |
| mmu-miR-218-1-3p | -0.027804891 | 0.963899737 |
| mmu-miR-218-2-3p | 0.103112859 | 0.833468543 |
| mmu-miR-219a-5p | 0.200221842 | 0.45395348 |
| mmu-miR-219a-1-3p | -0.201457047 | 0.747704981 |
| mmu-miR-219a-2-3p | -0.202493585 | 0.288058804 |
| mmu-miR-219b-3p | 0.439290488 | 0.880960819 |
| mmu-miR-219c-5p | 0.404866795 | 0.890836454 |
| mmu-miR-219c-3p | -0.475143248 | 0.891142935 |
| mmu-miR-21a-5p | -0.082610077 | 0.776708228 |
| mmu-miR-21a-3p | -1.436924616 | 0.724728327 |
| mmu-miR-21b | 0.206354734 | 0.918589104 |
| mmu-miR-21c | 1.422126101 | 0.517770572 |
| mmu-miR-22-5p | 0.426904298 | 0.099087016 |
| mmu-miR-22-3p | 0.040735295 | 0.868393924 |
| mmu-miR-221-5p | -0.731859817 | 0.002938092 |
| mmu-miR-221-3p | -0.225715394 | 0.341728592 |
| mmu-miR-222-5p | -0.088977219 | 0.772514248 |
| mmu-miR-222-3p | -0.182437347 | 0.313279662 |
| mmu-miR-223-5p | 1.17554937 | 0.520878559 |
| mmu-miR-223-3p | 0.147794136 | 0.619460472 |
| mmu-miR-224-5p | 0.995180317 | 0.071113362 |
| mmu-miR-224-3p | 0.191722612 | 0.930771391 |
| mmu-miR-23a-5p | -2.93836049 | 0.254005516 |
| mmu-miR-23a-3p | -0.064489928 | 0.745712924 |
| mmu-miR-23b-5p | -0.793119782 | 0.216249411 |
| mmu-miR-23b-3p | -0.182931261 | 0.293645569 |
| mmu-miR-24-1-5p | -0.024982029 | 0.918982434 |
| mmu-miR-24-3p | 0.042722168 | 0.837801408 |
| mmu-miR-24-2-5p | 0.197348108 | 0.331579347 |
| mmu-miR-25-5p | 0.172254545 | 0.765610194 |
| mmu-miR-25-3p | 0.248053383 | 0.170852259 |
| mmu-miR-26a-5p | -0.232957262 | 0.282755347 |
| mmu-miR-26a-1-3p | -0.305648999 | 0.525621879 |
| mmu-miR-26a-2-3p | 0.039415719 | 0.881250371 |
| mmu-miR-26b-5p | 0.01918973 | 0.912868832 |
| mmu-miR-26b-3p | 0.085514407 | 0.790177168 |
| mmu-miR-27a-5p | 0.497576113 | 0.378179674 |
| mmu-miR-27a-3p | 0.143463907 | 0.375912989 |
| mmu-miR-27b-5p | 0.069990658 | 0.791303031 |
| mmu-miR-27b-3p | -0.240221245 | 0.244001641 |
| mmu-miR-28a-5p | -0.070310773 | 0.707152184 |
| mmu-miR-28a-3p | -0.247462617 | 0.282887237 |
| mmu-miR-28c | -0.083786868 | 0.67376388 |
| mmu-miR-28b | -1.315242871 | 0.635800987 |
| mmu-miR-293-3p | 0.486628736 | 0.905071015 |
| mmu-miR-296-5p | 0.603900042 | 0.069225416 |
| mmu-miR-296-3p | 0.231260717 | 0.544032206 |
| mmu-miR-297a-5p | 3.238944443 | 0.062978975 |
| mmu-miR-669m-5p | 1.411780137 | 0.53280062 |
| mmu-miR-466m-5p | 1.411780137 | 0.53280062 |
| mmu-miR-297b-3p | 1.043880228 | 0.053046232 |
| mmu-miR-297a-3p | 1.043880228 | 0.053046232 |
| mmu-miR-297c-3p | 1.043880228 | 0.053046232 |
| mmu-miR-297b-5p | 0.089058557 | 0.960973302 |
| mmu-miR-297c-5p | 0.486628736 | 0.905071015 |
| mmu-miR-298-5p | 0.010122588 | 0.97021547 |
| mmu-miR-298-3p | -0.095689832 | 0.862327348 |
| mmu-miR-299a-5p | -0.170766217 | 0.502847276 |
| mmu-miR-299a-3p | 0.009356604 | 0.979880528 |
| mmu-miR-299b-5p | -0.174206286 | 0.494313459 |
| mmu-miR-299b-3p | 0.013672815 | 0.970503603 |
| mmu-miR-29a-5p | -0.054625289 | 0.861577552 |
| mmu-miR-29a-3p | -0.161563049 | 0.321184626 |
| mmu-miR-29b-1-5p | -0.393633651 | 0.512575816 |
| mmu-miR-29b-3p | -0.267970715 | 0.240763187 |
| mmu-miR-29b-2-5p | -0.231966682 | 0.308713778 |
| mmu-miR-29c-5p | -0.338079932 | 0.057957979 |
| mmu-miR-29c-3p | 0.035566077 | 0.825791878 |
| mmu-miR-300-5p | 0.140058182 | 0.741186432 |
| mmu-miR-300-3p | -0.016125327 | 0.934726623 |
| mmu-miR-301a-5p | -0.078481299 | 0.724854588 |
| mmu-miR-301a-3p | -0.266046241 | 0.24916261 |
| mmu-miR-301b-5p | -0.460644556 | 0.696643464 |
| mmu-miR-301b-3p | 0.283839006 | 0.646871954 |
| mmu-miR-302a-5p | 0.486628736 | 0.905071015 |
| mmu-miR-302b-3p | -1.627431379 | 0.463673795 |
| mmu-miR-302c-3p | 0.486628736 | 0.905071015 |
| mmu-miR-302d-3p | 0.405472397 | 0.8848111 |
| mmu-miR-3057-5p | -0.234135503 | 0.49515967 |
| mmu-miR-3057-3p | 0.722590849 | 0.330383716 |
| mmu-miR-3058-5p | 0.420173596 | 0.561890032 |
| mmu-miR-3058-3p | -0.475143652 | 0.901530955 |
| mmu-miR-3059-5p | -0.003326345 | 0.988059987 |
| mmu-miR-3059-3p | -0.482944176 | 0.719607355 |
| mmu-miR-3060-3p | 0.036294526 | 0.922543272 |
| mmu-miR-3061-5p | -1.436924616 | 0.724728327 |
| mmu-miR-3062-5p | -0.020673486 | 0.977917575 |
| mmu-miR-3064-5p | -1.295956452 | 0.642095461 |
| mmu-miR-3064-3p | -1.436924616 | 0.724728327 |
| mmu-miR-3065-5p | 0.982482734 | 0.702195542 |
| mmu-miR-3066-5p | -0.810039306 | 0.213375185 |
| mmu-miR-3066-3p | -0.417931653 | 0.860323666 |
| mmu-miR-3067-5p | 0.418835885 | 0.880827786 |
| mmu-miR-3068-5p | -0.141908703 | 0.595198901 |
| mmu-miR-3068-3p | -0.323782624 | 0.417302671 |
| mmu-miR-3069-5p | -2.168732801 | 0.240706584 |
| mmu-miR-3069-3p | -1.436924616 | 0.724728327 |
| mmu-miR-3070-5p | -0.199405351 | 0.715556857 |
| mmu-miR-3070-3p | -0.475143237 | 0.890804407 |
| mmu-miR-3070-2-3p | 2.025919386 | 0.355433426 |
| mmu-miR-3072-5p | -1.07498576 | 0.618315209 |
| mmu-miR-3072-3p | -0.042144782 | 0.852865455 |
| mmu-miR-3074-5p | 0.156394248 | 0.789331093 |
| mmu-miR-3074-1-3p | -0.085382218 | 0.854669753 |
| mmu-miR-3075-5p | 0.486628736 | 0.905071015 |
| mmu-miR-3076-5p | -0.270275792 | 0.850932804 |
| mmu-miR-3076-3p | -0.429627152 | 0.53264595 |
| mmu-miR-3078-5p | -0.448405931 | 0.106655342 |
| mmu-miR-3078-3p | 0.048480339 | 0.973125386 |
| mmu-miR-3079-5p | 1.380395091 | 0.715139819 |
| mmu-miR-3079-3p | 1.419943146 | 0.724874287 |
| mmu-miR-3080-5p | 1.024268283 | 0.331129166 |
| mmu-miR-3080-3p | 2.360293333 | 0.426387245 |
| mmu-miR-3081-3p | 0.424045913 | 0.820057217 |
| mmu-miR-3082-5p | 1.993966667 | 0.537058569 |
| mmu-miR-3082-3p | -0.375540232 | 0.210415528 |
| mmu-miR-3083-5p | -0.084967948 | 0.802382267 |
| mmu-miR-3083-3p | -0.480259777 | 0.381329257 |
| mmu-miR-3084-5p | 0.486628736 | 0.905071015 |
| mmu-miR-3084-3p | -2.305616711 | 0.280683407 |
| mmu-miR-3085-5p | -0.45153431 | 0.601664623 |
| mmu-miR-3085-3p | -0.107945226 | 0.646722424 |
| mmu-miR-3086-5p | -0.322227807 | 0.368315166 |
| mmu-miR-3086-3p | -0.255792328 | 0.832084844 |
| mmu-miR-3087-5p | -2.129266601 | 0.379038482 |
| mmu-miR-3087-3p | -0.190715801 | 0.717899094 |
| mmu-miR-3088-5p | 0.486628736 | 0.905071015 |
| mmu-miR-3088-3p | 0.733513468 | 0.495042608 |
| mmu-miR-3089-5p | -1.436924616 | 0.724728327 |
| mmu-miR-3089-3p | 0.402535268 | 0.83337325 |
| mmu-miR-3090-3p | -2.275533165 | 0.504726832 |
| mmu-miR-3091-5p | 0.486628736 | 0.905071015 |
| mmu-miR-3091-3p | 0.742872382 | 0.686416129 |
| mmu-miR-3093-5p | 0.189114882 | 0.468809257 |
| mmu-miR-3093-3p | -0.272140312 | 0.656201845 |
| mmu-miR-3094-5p | 0.016019124 | 0.989394277 |
| mmu-miR-3094-3p | -1.230809659 | 0.560492799 |
| mmu-miR-3095-5p | 0.185402705 | 0.854703046 |
| mmu-miR-3095-3p | -0.291955669 | 0.783721069 |
| mmu-miR-3097-5p | 0.897301567 | 0.371714097 |
| mmu-miR-3097-3p | -0.42995355 | 0.735652759 |
| mmu-miR-3098-5p | -0.606637553 | 0.809251687 |
| mmu-miR-3098-3p | 0.405517379 | 0.884376667 |
| mmu-miR-3099-5p | 0.486628736 | 0.905071015 |
| mmu-miR-3099-3p | 0.266376678 | 0.23566898 |
| mmu-miR-30a-5p | -0.234237509 | 0.181208901 |
| mmu-miR-30a-3p | 0.05094288 | 0.801966177 |
| mmu-miR-30b-5p | 0.057302109 | 0.7737427 |
| mmu-miR-30b-3p | 0.02820612 | 0.919046882 |
| mmu-miR-30c-5p | -0.354933382 | 0.022918473 |
| mmu-miR-30c-1-3p | 0.017187052 | 0.944946578 |
| mmu-miR-30c-2-3p | -0.444988355 | 0.030933014 |
| mmu-miR-30d-5p | -0.183217508 | 0.401172751 |
| mmu-miR-30d-3p | 0.095444955 | 0.682313139 |
| mmu-miR-30e-5p | -0.315552589 | 0.076406563 |
| mmu-miR-30e-3p | 0.026725563 | 0.867268462 |
| mmu-miR-30f | -0.316869757 | 0.606788111 |
| mmu-miR-31-5p | -0.244589906 | 0.153701406 |
| mmu-miR-31-3p | 0.094136266 | 0.834822905 |
| mmu-miR-3100-5p | 1.364507625 | 0.719476388 |
| mmu-miR-3101-5p | -0.023408044 | 0.984205892 |
| mmu-miR-3101-3p | 0.486628736 | 0.905071015 |
| mmu-miR-3102-5p.2-5p | 0.000479621 | 0.999448051 |
| mmu-miR-3102-3p.2-3p | 0.214504686 | 0.603180415 |
| mmu-miR-3102-3p | -0.332075217 | 0.190445776 |
| mmu-miR-3103-5p | -1.295956452 | 0.642095461 |
| mmu-miR-3103-3p | 1.538855009 | 0.424306432 |
| mmu-miR-3104-5p | 0.486628736 | 0.905071015 |
| mmu-miR-3104-3p | -0.064939744 | 0.957811359 |
| mmu-miR-3105-5p | 1.96783924 | 0.491129529 |
| mmu-miR-3105-3p | 0.586709004 | 0.42017808 |
| mmu-miR-3106-5p | 2.017906773 | 0.322569848 |
| mmu-miR-3106-3p | -0.397180744 | 0.87828193 |
| mmu-miR-3108-5p | 0.486628736 | 0.905071015 |
| mmu-miR-3109-5p | -0.475143254 | 0.891326251 |
| mmu-miR-3109-3p | 2.360293333 | 0.426387245 |
| mmu-miR-3110-5p | 0.892582311 | 0.629081951 |
| mmu-miR-3112-5p | -1.436924616 | 0.724728327 |
| mmu-miR-3112-3p | -1.436924616 | 0.724728327 |
| mmu-miR-3113-5p | 1.419943146 | 0.724874287 |
| mmu-miR-3154 | -1.436924616 | 0.724728327 |
| mmu-miR-32-5p | 0.591870333 | 0.018239726 |
| mmu-miR-32-3p | 0.604047081 | 0.271921107 |
| mmu-miR-320-3p | 0.015973538 | 0.943906717 |
| mmu-miR-322-5p | -0.160500638 | 0.454413141 |
| mmu-miR-322-3p | 0.197668313 | 0.4753354 |
| mmu-miR-323-5p | 0.473212184 | 0.25937749 |
| mmu-miR-323-3p | 0.123572642 | 0.470922627 |
| mmu-miR-324-5p | 0.035205205 | 0.845038708 |
| mmu-miR-324-3p | 0.191737817 | 0.412394514 |
| mmu-miR-325-5p | -0.033065744 | 0.875534476 |
| mmu-miR-325-3p | -0.230496959 | 0.504914093 |
| mmu-miR-326-3p | 0.336098787 | 0.14304367 |
| mmu-miR-328-3p | -0.109001924 | 0.584747881 |
| mmu-miR-329-5p | -0.609989341 | 0.02659617 |
| mmu-miR-329-3p | -0.208720156 | 0.344368788 |
| mmu-miR-33-5p | 1.059581781 | 0.02805246 |
| mmu-miR-33-3p | 0.575295069 | 0.110645528 |
| mmu-miR-330-5p | -0.052483951 | 0.792512832 |
| mmu-miR-330-3p | -0.259708923 | 0.221913975 |
| mmu-miR-331-5p | -0.215449948 | 0.943512516 |
| mmu-miR-331-3p | -0.096477625 | 0.781591093 |
| mmu-miR-335-5p | -0.716001891 | 0.013212407 |
| mmu-miR-335-3p | -0.60109567 | 0.001522984 |
| mmu-miR-337-5p | 0.036690394 | 0.860647175 |
| mmu-miR-337-3p | 0.21605029 | 0.440062667 |
| mmu-miR-338-5p | -0.147403085 | 0.541375197 |
| mmu-miR-338-3p | -0.281385638 | 0.211534008 |
| mmu-miR-339-5p | 0.507309338 | 0.004778206 |
| mmu-miR-339-3p | 0.3294454 | 0.319041118 |
| mmu-miR-340-5p | -0.016535203 | 0.92300916 |
| mmu-miR-340-3p | -0.257171982 | 0.180822257 |
| mmu-miR-341-5p | -0.078030078 | 0.954000407 |
| mmu-miR-341-3p | 0.029789052 | 0.926615888 |
| mmu-miR-342-5p | 0.198297992 | 0.580803544 |
| mmu-miR-342-3p | -0.073791362 | 0.787008872 |
| mmu-miR-344-5p | -0.437145679 | 0.746734976 |
| mmu-miR-344-3p | -0.015375775 | 0.952945043 |
| mmu-miR-344b-3p | -0.580484338 | 0.034146964 |
| mmu-miR-344c-5p | 1.362660406 | 0.493812656 |
| mmu-miR-344c-3p | -0.120267695 | 0.721040949 |
| mmu-miR-344d-3p | -0.756704155 | 0.00064589 |
| mmu-miR-344d-1-5p | -0.624032212 | 0.476110372 |
| mmu-miR-344d-2-5p | -1.436924616 | 0.724728327 |
| mmu-miR-344d-3-5p | -0.451734401 | 0.067007792 |
| mmu-miR-344e-5p | -1.295866507 | 0.64311074 |
| mmu-miR-344e-3p | -0.311220296 | 0.794560377 |
| mmu-miR-344h-5p | -1.295866507 | 0.64311074 |
| mmu-miR-344f-5p | -0.277133783 | 0.734255281 |
| mmu-miR-344f-3p | 0.712934785 | 0.138343384 |
| mmu-miR-344g-5p | -2.25262031 | 0.511938136 |
| mmu-miR-344g-3p | 0.481076735 | 0.620954203 |
| mmu-miR-344h-3p | -1.138502816 | 0.396745786 |
| mmu-miR-344i | -0.894085165 | 0.683820512 |
| mmu-miR-345-5p | -0.134826248 | 0.627700016 |
| mmu-miR-345-3p | -0.538675588 | 0.012237539 |
| mmu-miR-346-5p | -0.169735466 | 0.482306533 |
| mmu-miR-346-3p | -1.988173635 | 0.421792628 |
| mmu-miR-3470a | 0.961871416 | 0.014268202 |
| mmu-miR-3470b | 1.121627382 | 0.011950383 |
| mmu-miR-3471 | 1.292408215 | 0.297667565 |
| mmu-miR-3472 | 1.234062906 | 0.298951473 |
| mmu-miR-3473a | 2.479576116 | 0.091117596 |
| mmu-miR-3473b | 0.567296917 | 0.692835687 |
| mmu-miR-3473d | -0.02014596 | 0.986075161 |
| mmu-miR-3473e | 0.180723512 | 0.913218842 |
| mmu-miR-3473f | 1.388015401 | 0.730997715 |
| mmu-miR-3473g | 0.214699198 | 0.929879118 |
| mmu-miR-3474 | 1.957209093 | 0.548292466 |
| mmu-miR-3475-5p | -0.47514328 | 0.892084864 |
| mmu-miR-3475-3p | -0.405602423 | 0.202754733 |
| mmu-miR-34a-5p | 0.156631568 | 0.642271538 |
| mmu-miR-34a-3p | 1.022327688 | 0.336740132 |
| mmu-miR-34b-5p | 0.210963306 | 0.593766871 |
| mmu-miR-34b-3p | 0.390198884 | 0.270067549 |
| mmu-miR-34c-5p | 0.003610403 | 0.992313606 |
| mmu-miR-34c-3p | 0.24129739 | 0.554716593 |
| mmu-miR-350-5p | -0.099034222 | 0.796896352 |
| mmu-miR-350-3p | 0.140329616 | 0.482215026 |
| mmu-miR-351-5p | -0.10793915 | 0.675823513 |
| mmu-miR-351-3p | 0.753100701 | 0.458800744 |
| mmu-miR-3535 | -0.43710373 | 0.441844612 |
| mmu-miR-3544-3p | -1.436924616 | 0.724728327 |
| mmu-miR-3547-3p | -0.120040715 | 0.895264165 |
| mmu-miR-3552 | 0.200540249 | 0.670713578 |
| mmu-miR-3569-3p | 1.440214771 | 0.547076499 |
| mmu-miR-3572-5p | 0.486628736 | 0.905071015 |
| mmu-miR-3572-3p | 0.512915611 | 0.635475586 |
| mmu-miR-361-5p | -0.300828779 | 0.302287357 |
| mmu-miR-361-3p | -0.402663587 | 0.059535713 |
| mmu-miR-362-5p | -0.420660198 | 0.125293548 |
| mmu-miR-362-3p | 0.154193947 | 0.52768318 |
| mmu-miR-363-5p | -1.057552193 | 0.598530737 |
| mmu-miR-363-3p | 0.322048429 | 0.444984326 |
| mmu-miR-365-1-5p | -0.335372013 | 0.860770284 |
| mmu-miR-365-3p | -0.35410009 | 0.221354356 |
| mmu-miR-365-2-5p | -1.313965166 | 0.368483222 |
| mmu-miR-369-5p | -0.207985651 | 0.225132573 |
| mmu-miR-369-3p | -0.132514931 | 0.436915003 |
| mmu-miR-370-5p | -0.098186395 | 0.729411469 |
| mmu-miR-370-3p | -0.216863408 | 0.302395867 |
| mmu-miR-374b-5p | 0.380295833 | 0.264854565 |
| mmu-miR-374c-5p | 0.379853305 | 0.265184672 |
| mmu-miR-374c-3p | -0.153112351 | 0.521262807 |
| mmu-miR-375-3p | 0.37199254 | 0.15735551 |
| mmu-miR-376a-5p | -0.251393218 | 0.212028647 |
| mmu-miR-376a-3p | 0.638121165 | 0.114528729 |
| mmu-miR-376b-5p | -0.070139874 | 0.690223924 |
| mmu-miR-376b-3p | 0.027571817 | 0.89115076 |
| mmu-miR-376c-5p | 0.194633098 | 0.584454099 |
| mmu-miR-376c-3p | 0.060129353 | 0.818144298 |
| mmu-miR-377-5p | -0.246582946 | 0.255517224 |
| mmu-miR-377-3p | -0.555128603 | 0.052669696 |
| mmu-miR-378a-5p | 0.390179549 | 0.073933349 |
| mmu-miR-378a-3p | 0.247674183 | 0.2115528 |
| mmu-novel-11 | 0.390179549 | 0.073933349 |
| mmu-miR-378b | 0.291133809 | 0.410027949 |
| mmu-miR-378c | 0.301314325 | 0.131401428 |
| mmu-miR-378d | 0.594646921 | 0.016792295 |
| mmu-miR-379-5p | -0.020130006 | 0.938601268 |
| mmu-miR-379-3p | -0.291835088 | 0.099081206 |
| mmu-miR-380-5p | -0.14182724 | 0.64652832 |
| mmu-miR-380-3p | -0.31533629 | 0.145755455 |
| mmu-miR-381-5p | -0.112637139 | 0.759136732 |
| mmu-miR-381-3p | -0.111576391 | 0.644185821 |
| mmu-miR-382-5p | -0.087245358 | 0.697285841 |
| mmu-miR-382-3p | -0.228182593 | 0.234936271 |
| mmu-miR-383-5p | -0.217777249 | 0.357187084 |
| mmu-miR-383-3p | -0.210511427 | 0.724429152 |
| mmu-miR-384-5p | -0.405190699 | 0.100085115 |
| mmu-miR-384-3p | -0.230692115 | 0.197484222 |
| mmu-miR-3962 | 0.002962548 | 0.994732942 |
| mmu-miR-3963 | 1.12008573 | 0.031800522 |
| mmu-miR-3964 | 1.148756783 | 0.323047017 |
| mmu-miR-3965 | -2.275533165 | 0.504726832 |
| mmu-miR-3966 | 0.515909581 | 0.596458019 |
| mmu-miR-3967 | -0.145545194 | 0.9614684 |
| mmu-miR-3968 | 0.711494385 | 0.002538336 |
| mmu-miR-3969 | 0.717625299 | 0.233327323 |
| mmu-miR-3970 | -0.077168847 | 0.823090361 |
| mmu-miR-3971 | 1.957209093 | 0.548292466 |
| mmu-miR-409-5p | -0.554556961 | 0.028848173 |
| mmu-miR-409-3p | -0.238692695 | 0.151783627 |
| mmu-miR-410-5p | 0.157947745 | 0.873992476 |
| mmu-miR-410-3p | -0.128880697 | 0.51815571 |
| mmu-miR-411-5p | -0.098525298 | 0.681915336 |
| mmu-miR-411-3p | -0.360174494 | 0.057619052 |
| mmu-miR-412-5p | -0.317521333 | 0.140893657 |
| mmu-miR-412-3p | -0.067817105 | 0.886973595 |
| mmu-miR-421-5p | 0.335776436 | 0.792343634 |
| mmu-miR-421-3p | -0.270039765 | 0.127138859 |
| mmu-miR-423-5p | -0.177812159 | 0.38417225 |
| mmu-miR-423-3p | -0.089490744 | 0.62510227 |
| mmu-miR-425-5p | -0.103772663 | 0.662474043 |
| mmu-miR-425-3p | -0.163996643 | 0.467435739 |
| mmu-miR-429-3p | 5.180026931 | 1 |
| mmu-miR-431-5p | 0.287866938 | 0.303065761 |
| mmu-miR-431-3p | -0.168259299 | 0.350893594 |
| mmu-miR-433-5p | -0.3068505 | 0.137610977 |
| mmu-miR-433-3p | -0.095543281 | 0.706582912 |
| mmu-miR-434-5p | -0.739160369 | 6.36E-06 |
| mmu-miR-434-3p | -0.239567411 | 0.197783409 |
| mmu-miR-448-5p | 0.160361204 | 0.798205256 |
| mmu-miR-448-3p | 0.597786269 | 0.58534606 |
| mmu-miR-449a-5p | 0.292552696 | 0.565518294 |
| mmu-miR-449a-3p | -1.574543963 | 0.595739738 |
| mmu-miR-449b | 1.983481473 | 0.540267438 |
| mmu-miR-449c-5p | 2.372815347 | 0.359917735 |
| mmu-miR-449c-3p | 0.486628736 | 0.905071015 |
| mmu-miR-450a-5p | 0.182947089 | 0.536516213 |
| mmu-miR-450a-1-3p | 0.194110533 | 0.803961098 |
| mmu-miR-450a-2-3p | -0.383116508 | 0.806428403 |
| mmu-miR-450b-5p | -0.576177348 | 0.143194642 |
| mmu-miR-450b-3p | 0.915414867 | 0.314723919 |
| mmu-miR-451a | 0.229180414 | 0.362365506 |
| mmu-miR-452-5p | 1.403548184 | 0.708807238 |
| mmu-miR-452-3p | 0.486628736 | 0.905071015 |
| mmu-miR-455-5p | 0.348363908 | 0.44824403 |
| mmu-miR-455-3p | 0.405815045 | 0.163324317 |
| mmu-miR-465a-5p | -1.792620152 | 0.481729657 |
| mmu-miR-465a-3p | 1.388015401 | 0.730997715 |
| mmu-miR-465b-3p | 1.388015401 | 0.730997715 |
| mmu-miR-465c-3p | 1.388015401 | 0.730997715 |
| mmu-miR-465b-5p | 0.486628736 | 0.905071015 |
| mmu-miR-465c-5p | 0.486628736 | 0.905071015 |
| mmu-miR-466a-5p | 1.380395091 | 0.715139819 |
| mmu-miR-466a-3p | -0.195329867 | 0.52584809 |
| mmu-miR-466b-3p | -0.195329867 | 0.52584809 |
| mmu-miR-466c-3p | -0.195329867 | 0.52584809 |
| mmu-miR-466e-3p | -0.195329867 | 0.52584809 |
| mmu-miR-466p-5p | 1.380395091 | 0.715139819 |
| mmu-miR-466p-3p | -0.195329867 | 0.52584809 |
| mmu-miR-466b-5p | 0.131210234 | 0.920497757 |
| mmu-miR-466o-5p | 0.131210234 | 0.920497757 |
| mmu-miR-466e-5p | 0.539176225 | 0.707175534 |
| mmu-miR-466c-5p | -0.163574491 | 0.790985933 |
| mmu-miR-466h-3p | 1.650748092 | 0.099810369 |
| mmu-miR-466d-5p | 1.978469362 | 0.541785938 |
| mmu-miR-466d-3p | -0.095533938 | 0.737927784 |
| mmu-miR-466n-5p | 1.978469362 | 0.541785938 |
| mmu-miR-466f-5p | 2.360293333 | 0.426387245 |
| mmu-miR-466f-3p | 0.195673369 | 0.869535329 |
| mmu-miR-466f | 2.360293333 | 0.426387245 |
| mmu-miR-466g | 0.306381097 | 0.57893866 |
| mmu-miR-466h-5p | 0.744673913 | 0.646748185 |
| mmu-miR-466i-5p | 1.685278162 | 0.001581993 |
| mmu-miR-466i-3p | 1.067773146 | 0.10744174 |
| mmu-miR-466j | 1.339961124 | 0.740237326 |
| mmu-miR-466k | -0.475143652 | 0.901530955 |
| mmu-miR-466l-3p | 1.419943146 | 0.724874287 |
| mmu-miR-466m-3p | -1.436924616 | 0.724728327 |
| mmu-miR-466n-3p | 0.381218221 | 0.758595008 |
| mmu-miR-466o-3p | -1.537779222 | 0.608631699 |
| mmu-miR-466q | 0.7053173 | 0.741304172 |
| mmu-miR-467a-5p | -0.209764329 | 0.536066607 |
| mmu-miR-467a-3p | 0.234850103 | 0.448342776 |
| mmu-miR-467b-5p | -0.209764329 | 0.536066607 |
| mmu-miR-467d-3p | 0.234850103 | 0.448342776 |
| mmu-miR-467b-3p | 0.982482734 | 0.702195542 |
| mmu-miR-467c-5p | -0.360051225 | 0.456670827 |
| mmu-miR-467c-3p | 0.816207251 | 0.655275679 |
| mmu-miR-467d-5p | 0.661874974 | 0.108390392 |
| mmu-miR-467e-5p | 0.612372128 | 0.079904195 |
| mmu-miR-467e-3p | 2.026144898 | 0.365058652 |
| mmu-miR-467f | -2.275533165 | 0.504726832 |
| mmu-miR-467g | -2.55731867 | 0.485957836 |
| mmu-miR-470-5p | -0.047701235 | 0.982294863 |
| mmu-miR-471-5p | -0.475143652 | 0.901530955 |
| mmu-miR-471-3p | 0.486628736 | 0.905071015 |
| mmu-miR-483-5p | 1.448724145 | 0.212821517 |
| mmu-miR-483-3p | 0.764358698 | 0.350169656 |
| mmu-miR-484 | -0.059290398 | 0.792607062 |
| mmu-miR-485-5p | -0.339636418 | 0.087289402 |
| mmu-miR-485-3p | 0.075211604 | 0.677021515 |
| mmu-miR-486a-5p | -0.1128341 | 0.6981169 |
| mmu-miR-486a-3p | 0.453728273 | 0.621688863 |
| mmu-miR-486b-5p | -0.1128341 | 0.6981169 |
| mmu-miR-486b-3p | 0.453728273 | 0.621688863 |
| mmu-miR-487b-5p | -0.380433655 | 0.643237695 |
| mmu-miR-487b-3p | 0.05394809 | 0.724866952 |
| mmu-miR-488-5p | -0.727297157 | 0.013445694 |
| mmu-miR-488-3p | -0.225200435 | 0.188531065 |
| mmu-miR-489-3p | -2.525243507 | 0.429754336 |
| mmu-miR-490-5p | 1.036710814 | 0.018927744 |
| mmu-miR-490-3p | 0.72427113 | 0.112651261 |
| mmu-miR-491-5p | -0.212589823 | 0.377013019 |
| mmu-miR-491-3p | 0.26536018 | 0.554188294 |
| mmu-miR-493-5p | -0.085653692 | 0.761522997 |
| mmu-miR-493-3p | -0.145090882 | 0.812852745 |
| mmu-miR-494-5p | 1.973150242 | 0.603808174 |
| mmu-miR-494-3p | -0.581468146 | 0.001723741 |
| mmu-miR-495-5p | -0.708817668 | 0.356466408 |
| mmu-miR-495-3p | -0.5060617 | 0.008064462 |
| mmu-miR-496a-5p | -0.188782217 | 0.90005588 |
| mmu-miR-496a-3p | -0.169944056 | 0.362355544 |
| mmu-miR-497a-5p | 0.273256088 | 0.275565691 |
| mmu-miR-497a-3p | 0.701050964 | 0.605622379 |
| mmu-miR-497b | 2.598945878 | 0.150340926 |
| mmu-miR-499-5p | 0.272962454 | 0.266372053 |
| mmu-miR-499-3p | 0.425862971 | 0.89814895 |
| mmu-miR-500-5p | 0.244287332 | 0.720086179 |
| mmu-miR-500-3p | 0.449572389 | 0.023488174 |
| mmu-miR-501-5p | -0.312918629 | 0.233140127 |
| mmu-miR-501-3p | -0.128780072 | 0.643638332 |
| mmu-miR-503-5p | 0.145869679 | 0.716274746 |
| mmu-miR-503-3p | -0.072680008 | 0.82887042 |
| mmu-miR-504-5p | 0.071741261 | 0.73449653 |
| mmu-miR-504-3p | -0.784719261 | 0.583268046 |
| mmu-miR-505-5p | -0.099098592 | 0.867217488 |
| mmu-miR-505-3p | -0.358622442 | 0.255944234 |
| mmu-miR-5099 | 0.07315796 | 0.780480231 |
| mmu-miR-5100 | 0.452370206 | 0.654388866 |
| mmu-miR-5101 | 0.686877194 | 0.608526147 |
| mmu-miR-5103 | -0.993777127 | 0.521404746 |
| mmu-miR-5104 | 0.486628736 | 0.905071015 |
| mmu-miR-5106 | 1.403548184 | 0.708807238 |
| mmu-miR-5107-3p | 0.486628736 | 0.905071015 |
| mmu-miR-511-5p | -0.000703315 | 0.999550923 |
| mmu-miR-511-3p | 1.199428857 | 0.011023973 |
| mmu-miR-5113 | -1.436924616 | 0.724728327 |
| mmu-miR-5121 | 4.467184493 | 9.38E-09 |
| mmu-miR-5122 | -0.675230677 | 0.700580505 |
| mmu-miR-5123 | -1.06559519 | 0.74895328 |
| mmu-miR-5125 | 0.055477931 | 0.926197312 |
| mmu-miR-5126 | 0.889116615 | 0.505567574 |
| mmu-miR-5128 | -0.834077824 | 0.638170184 |
| mmu-miR-5129-5p | 0.968085321 | 0.234218391 |
| mmu-miR-5129-3p | 0.304158372 | 0.312548091 |
| mmu-miR-5132-5p | 1.092192154 | 0.624731453 |
| mmu-miR-5132-3p | -0.516134802 | 0.81613928 |
| mmu-miR-5134-5p | -1.436924616 | 0.724728327 |
| mmu-miR-5134-3p | 1.364507625 | 0.719476388 |
| mmu-miR-532-5p | 0.047481478 | 0.815462641 |
| mmu-miR-532-3p | 0.382903452 | 0.05669247 |
| mmu-miR-539-5p | -0.054249646 | 0.863473766 |
| mmu-miR-539-3p | 0.399550707 | 0.758514109 |
| mmu-miR-540-5p | -0.153709989 | 0.530107715 |
| mmu-miR-540-3p | -0.07256874 | 0.765942918 |
| mmu-miR-541-5p | -0.803837722 | 4.43E-05 |
| mmu-miR-541-3p | -0.483631359 | 0.029895753 |
| mmu-miR-542-5p | 0.308689738 | 0.827141421 |
| mmu-miR-542-3p | 0.286807385 | 0.364412745 |
| mmu-miR-543-5p | -0.419748581 | 0.256065113 |
| mmu-miR-543-3p | -0.142595171 | 0.55743407 |
| mmu-miR-544-5p | 0.136662221 | 0.595801965 |
| mmu-miR-544-3p | -0.267610625 | 0.350845556 |
| mmu-miR-547-5p | 0.486628736 | 0.905071015 |
| mmu-miR-547-3p | -0.198036222 | 0.637268666 |
| mmu-miR-551b-5p | -0.580265866 | 0.134209933 |
| mmu-miR-551b-3p | -0.104246448 | 0.637624731 |
| mmu-miR-5615-5p | 0.088502929 | 0.93813216 |
| mmu-miR-5615-3p | 1.403548184 | 0.708807238 |
| mmu-miR-5616-5p | 0.486628736 | 0.905071015 |
| mmu-miR-5617-5p | -0.071638854 | 0.957096537 |
| mmu-miR-5617-3p | -0.865040871 | 0.568052112 |
| mmu-miR-5619-5p | -0.210261108 | 0.897866173 |
| mmu-miR-5619-3p | -0.475143622 | 0.900866421 |
| mmu-miR-5620-5p | 0.484791247 | 0.754585643 |
| mmu-miR-5620-3p | 1.388015401 | 0.730997715 |
| mmu-miR-5621-5p | 0.486628736 | 0.905071015 |
| mmu-miR-5621-3p | -0.059880606 | 0.915621374 |
| mmu-miR-5623-5p | -1.315215045 | 0.636796657 |
| mmu-miR-5624-5p | -0.475143237 | 0.890804407 |
| mmu-miR-5624-3p | 0.540990961 | 0.818192768 |
| mmu-miR-5626-3p | 2.978673681 | 0.243444312 |
| mmu-miR-5627-5p | 0.486628736 | 0.905071015 |
| mmu-miR-5627-3p | 0.486628736 | 0.905071015 |
| mmu-miR-5709-5p | -0.867640443 | 0.518028382 |
| mmu-miR-5710 | 0.404866795 | 0.890836454 |
| mmu-miR-574-5p | 0.82913867 | 0.107325661 |
| mmu-miR-574-3p | 0.293610473 | 0.263809345 |
| mmu-miR-582-5p | -0.145304928 | 0.536201787 |
| mmu-miR-582-3p | -0.523091821 | 0.009652076 |
| mmu-miR-592-5p | 0.610912167 | 0.050308474 |
| mmu-miR-592-3p | 0.850902514 | 0.443840695 |
| mmu-miR-598-5p | -0.481644737 | 0.658581716 |
| mmu-miR-598-3p | -0.171494914 | 0.539977018 |
| mmu-miR-615-3p | -1.427826555 | 0.517710466 |
| mmu-miR-6236 | 0.487963607 | 0.57107644 |
| mmu-miR-6238 | 0.732130371 | 0.639404536 |
| mmu-miR-6239 | 1.749320802 | 0.316662368 |
| mmu-miR-6240 | 1.683445529 | 0.00909313 |
| mmu-miR-6371 | -1.436924616 | 0.724728327 |
| mmu-miR-6380 | 2.360293333 | 0.426387245 |
| mmu-miR-6384 | 0.486628736 | 0.905071015 |
| mmu-miR-6390 | 0.486628736 | 0.905071015 |
| mmu-miR-6392-5p | 0.538375636 | 0.66943574 |
| mmu-miR-6395 | 0.351007231 | 0.596094726 |
| mmu-miR-6412 | 2.570242602 | 0.205433438 |
| mmu-miR-6418-3p | -0.317864192 | 0.745000223 |
| mmu-miR-6419 | 0.486628736 | 0.905071015 |
| mmu-miR-6481 | 0.938968766 | 0.289576231 |
| mmu-miR-6516-5p | 0.839146286 | 0.688798999 |
| mmu-miR-6516-3p | 0.486628736 | 0.905071015 |
| mmu-miR-652-5p | -0.081221976 | 0.879283124 |
| mmu-miR-652-3p | 0.040631293 | 0.857138143 |
| mmu-miR-653-5p | -0.836279649 | 0.317527768 |
| mmu-miR-6537-3p | -0.895497844 | 0.708955871 |
| mmu-miR-6538 | 1.131811122 | 0.269241887 |
| mmu-miR-6539 | -0.054362619 | 0.979895964 |
| mmu-miR-6540-5p | -0.100453011 | 0.656625474 |
| mmu-miR-6540-3p | 0.907557675 | 0.337747532 |
| mmu-miR-6546-5p | 1.419943146 | 0.724874287 |
| mmu-miR-664-5p | -0.304095793 | 0.848587801 |
| mmu-miR-664-3p | -0.437832075 | 0.324569042 |
| mmu-miR-665-5p | -1.261713681 | 0.584023151 |
| mmu-miR-665-3p | 0.202616369 | 0.424430711 |
| mmu-miR-666-5p | -0.265672822 | 0.255983866 |
| mmu-miR-666-3p | 0.584812176 | 0.220711093 |
| mmu-miR-667-5p | -0.347593182 | 0.302711999 |
| mmu-miR-667-3p | -0.288836426 | 0.171258827 |
| mmu-miR-668-5p | -0.922745312 | 0.42257728 |
| mmu-miR-668-3p | -0.287222123 | 0.139040761 |
| mmu-miR-669a-5p | -0.025748268 | 0.943710558 |
| mmu-miR-669a-3p | 0.243538882 | 0.460537775 |
| mmu-miR-669o-3p | 0.243538882 | 0.460537775 |
| mmu-miR-669p-5p | -0.025748268 | 0.943710558 |
| mmu-miR-669a-3-3p | 0.57615927 | 0.781640894 |
| mmu-miR-669b-5p | 0.588298143 | 0.328421904 |
| mmu-miR-669b-3p | 1.433315652 | 0.566686712 |
| mmu-miR-669c-5p | 0.320777326 | 0.637896889 |
| mmu-miR-669c-3p | 0.155336866 | 0.943865225 |
| mmu-miR-669d-5p | 0.569262625 | 0.398194687 |
| mmu-miR-669e-5p | -0.908674813 | 0.261872222 |
| mmu-miR-669e-3p | 1.584514345 | 0.34324856 |
| mmu-miR-669f-5p | 1.835861411 | 0.044532017 |
| mmu-miR-669f-3p | 0.644616504 | 0.314159266 |
| mmu-miR-669h-5p | 0.271669489 | 0.702821823 |
| mmu-miR-669h-3p | 1.364507625 | 0.719476388 |
| mmu-miR-669k-5p | 0.5712749 | 0.626545228 |
| mmu-miR-669i | 1.923316019 | 0.617646858 |
| mmu-miR-669l-5p | -0.551540591 | 0.662714758 |
| mmu-miR-669l-3p | 0.486628736 | 0.905071015 |
| mmu-miR-669m-3p | 0.049304145 | 0.98003021 |
| mmu-miR-669o-5p | -0.18238143 | 0.792941931 |
| mmu-miR-669p-3p | 1.759875577 | 0.452095059 |
| mmu-miR-670-5p | 0.351441071 | 0.452973633 |
| mmu-miR-670-3p | 1.306778702 | 0.193225879 |
| mmu-miR-671-5p | -0.101077449 | 0.925286122 |
| mmu-miR-671-3p | 0.443049652 | 0.715705417 |
| mmu-miR-6715-5p | 1.029774798 | 0.319797994 |
| mmu-miR-672-5p | 0.283906787 | 0.26316983 |
| mmu-miR-672-3p | 0.058793434 | 0.878552625 |
| mmu-miR-673-5p | -0.368793939 | 0.094222484 |
| mmu-miR-673-3p | -0.244882939 | 0.368322575 |
| mmu-miR-674-5p | 0.036188479 | 0.849656061 |
| mmu-miR-674-3p | -0.076272658 | 0.657321067 |
| mmu-novel-283 | 0.056488446 | 0.770577183 |
| mmu-miR-676-5p | 0.009392944 | 0.967226648 |
| mmu-miR-676-3p | 0.243201724 | 0.196550945 |
| mmu-miR-6769b-5p | 0.528134529 | 0.753364621 |
| mmu-miR-6769b-3p | 0.871116526 | 0.670895008 |
| mmu-miR-677-5p | -0.146341727 | 0.934166629 |
| mmu-miR-679-5p | -0.880014284 | 0.037277812 |
| mmu-miR-679-3p | -0.526399303 | 0.509263103 |
| mmu-miR-682 | 1.419943146 | 0.724874287 |
| mmu-miR-6896-5p | -0.450094961 | 0.265159151 |
| mmu-miR-6896-3p | -1.436924616 | 0.724728327 |
| mmu-miR-6897-5p | 0.486628736 | 0.905071015 |
| mmu-miR-6898-5p | 0.486628736 | 0.905071015 |
| mmu-miR-6898-3p | 0.486628736 | 0.905071015 |
| mmu-miR-6899-3p | -0.047929689 | 0.977591544 |
| mmu-miR-690 | 0.858525576 | 0.002051366 |
| mmu-miR-6900-5p | -0.967356566 | 0.393842093 |
| mmu-miR-6900-3p | -1.436924616 | 0.724728327 |
| mmu-miR-6901-5p | -0.012222119 | 0.995464005 |
| mmu-miR-6901-3p | 0.223585564 | 0.865222471 |
| mmu-miR-6902-5p | -0.018688965 | 0.993089051 |
| mmu-miR-6902-3p | -1.436924616 | 0.724728327 |
| mmu-miR-6903-5p | 0.996571821 | 0.697624981 |
| mmu-miR-6903-3p | 1.978469362 | 0.541785938 |
| mmu-miR-6905-3p | 1.411972839 | 0.521203398 |
| mmu-miR-6906-5p | -0.475143225 | 0.890451144 |
| mmu-miR-6906-3p | -1.436924616 | 0.724728327 |
| mmu-miR-6907-3p | -0.519838638 | 0.777356382 |
| mmu-miR-6908-5p | -2.25262031 | 0.511938136 |
| mmu-miR-6908-3p | 0.486628736 | 0.905071015 |
| mmu-miR-6909-5p | 0.991623214 | 0.710967049 |
| mmu-miR-6910-3p | 1.744828457 | 0.418644784 |
| mmu-miR-6911-5p | -1.436924616 | 0.724728327 |
| mmu-miR-6911-3p | -0.081542821 | 0.931984122 |
| mmu-miR-6912-5p | -2.275533165 | 0.504726832 |
| mmu-miR-6912-3p | -2.93836049 | 0.254005516 |
| mmu-miR-6913-5p | -0.475143237 | 0.890804407 |
| mmu-miR-6913-3p | -0.27693391 | 0.896736768 |
| mmu-miR-6914-3p | -0.960467656 | 0.58448846 |
| mmu-miR-6915-5p | -1.436924616 | 0.724728327 |
| mmu-miR-6915-3p | -1.436924616 | 0.724728327 |
| mmu-miR-6916-3p | 0.486628736 | 0.905071015 |
| mmu-miR-6918-5p | -1.412983344 | 0.299930783 |
| mmu-miR-6919-5p | -0.321885286 | 0.82907205 |
| mmu-miR-6920-5p | 0.486628736 | 0.905071015 |
| mmu-miR-6920-3p | -1.436924616 | 0.724728327 |
| mmu-miR-6921-5p | 1.339961124 | 0.740237326 |
| mmu-miR-6921-3p | 1.339961124 | 0.740237326 |
| mmu-miR-6922-3p | 1.978469362 | 0.541785938 |
| mmu-miR-6925-5p | 1.952112232 | 0.549613728 |
| mmu-miR-6926-5p | 1.388015401 | 0.730997715 |
| mmu-miR-6927-3p | 0.486628736 | 0.905071015 |
| mmu-miR-6928-5p | 0.162730944 | 0.936872934 |
| mmu-miR-6928-3p | 1.422164712 | 0.529246609 |
| mmu-miR-6929-3p | -1.574543963 | 0.595739738 |
| mmu-miR-693-3p | -1.436924616 | 0.724728327 |
| mmu-miR-6931-3p | 0.486628736 | 0.905071015 |
| mmu-miR-6932-5p | -0.699658665 | 0.779376242 |
| mmu-miR-6932-3p | 0.486628736 | 0.905071015 |
| mmu-miR-6933-5p | -0.010911185 | 0.993026633 |
| mmu-miR-6933-3p | -1.436924616 | 0.724728327 |
| mmu-miR-6934-5p | -0.055725169 | 0.981011668 |
| mmu-miR-6935-3p | 0.486628736 | 0.905071015 |
| mmu-miR-6936-5p | -2.516040207 | 0.498035283 |
| mmu-miR-6936-3p | 0.755015568 | 0.62058507 |
| mmu-miR-6937-5p | 0.943417456 | 0.161924119 |
| mmu-miR-6937-3p | -0.08776891 | 0.932046835 |
| mmu-miR-6938-3p | -1.436924616 | 0.724728327 |
| mmu-miR-694 | -0.334235524 | 0.674426591 |
| mmu-miR-6940-3p | -0.608436406 | 0.609179086 |
| mmu-miR-6941-5p | 0.486628736 | 0.905071015 |
| mmu-miR-6941-3p | 0.486628736 | 0.905071015 |
| mmu-miR-6942-5p | 0.381214735 | 0.909477416 |
| mmu-miR-6943-5p | 0.486628736 | 0.905071015 |
| mmu-miR-6943-3p | 1.02926428 | 0.68700643 |
| mmu-miR-6944-5p | 0.382587187 | 0.90303006 |
| mmu-miR-6944-3p | -0.376592458 | 0.357381823 |
| mmu-miR-6945-3p | 0.609336993 | 0.722335225 |
| mmu-miR-6946-5p | 1.941335612 | 0.552895676 |
| mmu-miR-6946-3p | -0.335372013 | 0.860770284 |
| mmu-miR-6947-5p | 1.403548184 | 0.708807238 |
| mmu-miR-6948-5p | -0.812434145 | 0.361091784 |
| mmu-miR-6948-3p | -0.413499173 | 0.669030097 |
| mmu-miR-6950-3p | 0.486628736 | 0.905071015 |
| mmu-miR-6951-5p | -0.115893087 | 0.902778039 |
| mmu-miR-6952-5p | 0.454336398 | 0.884374757 |
| mmu-miR-6952-3p | -0.043251133 | 0.986980632 |
| mmu-miR-6953-3p | -0.185057808 | 0.833881714 |
| mmu-miR-6955-5p | 1.96783924 | 0.491129529 |
| mmu-miR-6955-3p | 0.404866795 | 0.890836454 |
| mmu-miR-6958-3p | -1.436924616 | 0.724728327 |
| mmu-miR-6959-5p | 1.759875577 | 0.452095059 |
| mmu-miR-6959-3p | -1.066309771 | 0.747072066 |
| mmu-miR-696 | 0.486628736 | 0.905071015 |
| mmu-miR-6962-5p | 2.372815347 | 0.359917735 |
| mmu-miR-6962-3p | -0.051300486 | 0.982513035 |
| mmu-miR-6964-3p | 1.941335612 | 0.552895676 |
| mmu-miR-6966-5p | 0.642122356 | 0.708572473 |
| mmu-miR-6967-5p | 1.411972839 | 0.521203398 |
| mmu-miR-6970-5p | 0.918925709 | 0.652815946 |
| mmu-miR-6970-3p | -0.426765677 | 0.617744709 |
| mmu-miR-6971-3p | 1.109775208 | 0.618523474 |
| mmu-miR-6973b-3p | 0.41878717 | 0.886847101 |
| mmu-miR-6975-3p | -1.436924616 | 0.724728327 |
| mmu-miR-6976-5p | -1.436924616 | 0.724728327 |
| mmu-miR-6977-3p | -0.185699355 | 0.740414193 |
| mmu-miR-6979-3p | -0.475143622 | 0.900866421 |
| mmu-miR-698-5p | -2.275533165 | 0.504726832 |
| mmu-miR-698-3p | -1.436924616 | 0.724728327 |
| mmu-miR-6980-3p | 1.380395091 | 0.715139819 |
| mmu-miR-6982-5p | -1.436924616 | 0.724728327 |
| mmu-miR-6982-3p | 0.514067461 | 0.648792439 |
| mmu-miR-6983-5p | -0.475143602 | 0.900417779 |
| mmu-miR-6983-3p | 1.005978046 | 0.678539537 |
| mmu-miR-6984-5p | -0.47514328 | 0.892084864 |
| mmu-miR-6984-3p | 0.277672866 | 0.880139897 |
| mmu-miR-6985-5p | 0.486628736 | 0.905071015 |
| mmu-miR-6985-3p | 0.941422732 | 0.645277258 |
| mmu-miR-6986-5p | 2.980497203 | 0.185619717 |
| mmu-miR-6987-5p | -2.927255144 | 0.312445041 |
| mmu-miR-6989-3p | -1.059000868 | 0.304793022 |
| mmu-miR-6990-5p | -0.040275675 | 0.967755969 |
| mmu-miR-6991-5p | -0.475143602 | 0.900417779 |
| mmu-miR-6991-3p | 0.439290488 | 0.880960819 |
| mmu-miR-6994-5p | -3.386445261 | 0.196485323 |
| mmu-miR-6994-3p | 0.601946967 | 0.645940838 |
| mmu-miR-6996-5p | 0.650500941 | 0.577410261 |
| mmu-miR-6997-5p | 0.425697891 | 0.891586632 |
| mmu-miR-6997-3p | -1.430123507 | 0.33735947 |
| mmu-miR-6998-5p | 1.339961124 | 0.740237326 |
| mmu-miR-6998-3p | 0.130733115 | 0.93877976 |
| mmu-miR-6999-5p | 1.339961124 | 0.740237326 |
| mmu-miR-6999-3p | 1.011009551 | 0.738807256 |
| mmu-miR-700-5p | 0.373265626 | 0.245201877 |
| mmu-miR-700-3p | 1.339961124 | 0.740237326 |
| mmu-miR-7000-3p | 0.486628736 | 0.905071015 |
| mmu-miR-7004-5p | 0.438583487 | 0.874929839 |
| mmu-miR-7006-3p | -2.25262031 | 0.511938136 |
| mmu-miR-7008-5p | 0.438583487 | 0.874929839 |
| mmu-miR-7008-3p | 0.981845019 | 0.714113825 |
| mmu-miR-7009-3p | 0.486628736 | 0.905071015 |
| mmu-miR-701-5p | 2.980497203 | 0.185619717 |
| mmu-miR-701-3p | 0.449680796 | 0.77758165 |
| mmu-miR-7010-5p | 0.486628736 | 0.905071015 |
| mmu-miR-7012-5p | -1.436924616 | 0.724728327 |
| mmu-miR-7013-5p | -0.24846552 | 0.722081271 |
| mmu-miR-7013-3p | -1.971292818 | 0.387147464 |
| mmu-miR-7015-5p | -0.475143237 | 0.890804407 |
| mmu-miR-7015-3p | -0.283161997 | 0.739108215 |
| mmu-miR-7016-5p | -2.516040207 | 0.498035283 |
| mmu-miR-7016-3p | -1.436924616 | 0.724728327 |
| mmu-miR-7017-5p | 3.368750193 | 0.105130997 |
| mmu-miR-7018-3p | -1.436924616 | 0.724728327 |
| mmu-miR-7019-5p | -1.436924616 | 0.724728327 |
| mmu-miR-7019-3p | 1.952112232 | 0.549613728 |
| mmu-miR-702-5p | -1.578650505 | 0.598830144 |
| mmu-miR-702-3p | 0.001891886 | 0.998005224 |
| mmu-miR-7021-5p | 1.025959055 | 0.307352054 |
| mmu-miR-7021-3p | 0.486628736 | 0.905071015 |
| mmu-miR-7022-5p | 0.425697891 | 0.891586632 |
| mmu-miR-7022-3p | -1.436924616 | 0.724728327 |
| mmu-miR-7024-5p | 1.380395091 | 0.715139819 |
| mmu-miR-7024-3p | 2.391684183 | 0.354273348 |
| mmu-miR-7025-3p | 0.263666757 | 0.881010214 |
| mmu-miR-7026-5p | -0.559005435 | 0.682598445 |
| mmu-miR-7027-5p | -1.436924616 | 0.724728327 |
| mmu-miR-7027-3p | -0.808623946 | 0.565320853 |
| mmu-miR-7029-5p | -1.436924616 | 0.724728327 |
| mmu-miR-7029-3p | -2.305616711 | 0.280683407 |
| mmu-miR-703 | 0.486628736 | 0.905071015 |
| mmu-miR-7031-5p | 1.403548184 | 0.708807238 |
| mmu-miR-7031-3p | 0.486628736 | 0.905071015 |
| mmu-miR-7032-3p | 2.235282595 | 0.259281496 |
| mmu-miR-7034-3p | 0.405472397 | 0.8848111 |
| mmu-miR-7036a-5p | 0.486628736 | 0.905071015 |
| mmu-miR-7036a-3p | -2.757785938 | 0.360996028 |
| mmu-miR-7037-5p | -1.315261827 | 0.635126118 |
| mmu-miR-7037-3p | 1.339961124 | 0.740237326 |
| mmu-miR-7038-5p | -1.537299289 | 0.607033397 |
| mmu-miR-7038-3p | -0.47514328 | 0.892084864 |
| mmu-miR-7039-5p | 0.425862971 | 0.89814895 |
| mmu-miR-7039-3p | -2.25262031 | 0.511938136 |
| mmu-miR-704 | 0.849917245 | 0.424110829 |
| mmu-miR-7040-5p | 0.486628736 | 0.905071015 |
| mmu-miR-7041-5p | -1.436924616 | 0.724728327 |
| mmu-miR-7042-5p | 1.388015401 | 0.730997715 |
| mmu-miR-7043-3p | -1.988072381 | 0.420921115 |
| mmu-miR-7044-3p | 2.411886909 | 0.410802766 |
| mmu-miR-7046-3p | -0.649353775 | 0.744099014 |
| mmu-miR-7047-5p | 0.142632724 | 0.94851098 |
| mmu-miR-7047-3p | 0.260481029 | 0.455498804 |
| mmu-miR-7048-5p | 0.486628736 | 0.905071015 |
| mmu-miR-7048-3p | 1.941335612 | 0.552895676 |
| mmu-miR-7051-5p | 0.486628736 | 0.905071015 |
| mmu-miR-7052-5p | 1.380395091 | 0.715139819 |
| mmu-miR-7052-3p | -0.47514326 | 0.89150293 |
| mmu-miR-7054-5p | -0.350955084 | 0.864117339 |
| mmu-miR-7054-3p | 0.486628736 | 0.905071015 |
| mmu-miR-7055-5p | -2.25262031 | 0.511938136 |
| mmu-miR-7055-3p | -1.261713681 | 0.584023151 |
| mmu-miR-7056-5p | 0.486628736 | 0.905071015 |
| mmu-miR-7056-3p | -2.011456038 | 0.620417902 |
| mmu-miR-7057-3p | 0.486628736 | 0.905071015 |
| mmu-miR-7058-3p | -0.208423155 | 0.938535121 |
| mmu-miR-7059-5p | 0.317831455 | 0.807179312 |
| mmu-miR-706 | -1.125547727 | 0.297805695 |
| mmu-miR-7061-5p | 2.006083459 | 0.594594404 |
| mmu-miR-7062-5p | 0.439290488 | 0.880960819 |
| mmu-miR-7063-5p | -0.304963829 | 0.755992236 |
| mmu-miR-7063-3p | -0.475143254 | 0.891326251 |
| mmu-miR-7064-5p | 1.380395091 | 0.715139819 |
| mmu-miR-7065-5p | -1.436924616 | 0.724728327 |
| mmu-miR-7065-3p | -0.364986868 | 0.764358267 |
| mmu-miR-7066-5p | 0.996555386 | 0.698389844 |
| mmu-miR-7066-3p | 2.001215214 | 0.327363226 |
| mmu-miR-7068-3p | 1.327923101 | 0.186312797 |
| mmu-miR-7069-5p | 1.403548184 | 0.708807238 |
| mmu-miR-7069-3p | -2.275533165 | 0.504726832 |
| mmu-miR-7070-5p | -0.47514326 | 0.89150293 |
| mmu-miR-7070-3p | -1.295866507 | 0.64311074 |
| mmu-miR-7071-5p | 0.486628736 | 0.905071015 |
| mmu-miR-7073-5p | 1.377539829 | 0.522375287 |
| mmu-miR-7075-5p | 0.486628736 | 0.905071015 |
| mmu-miR-7075-3p | 0.762759838 | 0.626564067 |
| mmu-miR-7077-5p | -1.436924616 | 0.724728327 |
| mmu-miR-7077-3p | -1.436924616 | 0.724728327 |
| mmu-miR-708-5p | 0.022119332 | 0.916686222 |
| mmu-miR-708-3p | 0.215645157 | 0.318349716 |
| mmu-miR-7080-5p | -0.547798284 | 0.390665656 |
| mmu-miR-7080-3p | -0.543222109 | 0.04624282 |
| mmu-miR-7081-3p | -0.184620741 | 0.901700925 |
| mmu-miR-7083-5p | 0.947175999 | 0.532702503 |
| mmu-miR-7084-5p | -1.436924616 | 0.724728327 |
| mmu-miR-7085-5p | 0.486628736 | 0.905071015 |
| mmu-miR-7085-3p | 1.994065782 | 0.329426582 |
| mmu-miR-7087-5p | 0.176264711 | 0.931784742 |
| mmu-miR-709 | 0.646442434 | 0.603814818 |
| mmu-miR-7092-5p | -0.388781874 | 0.742460654 |
| mmu-miR-7092-3p | -0.180405451 | 0.913001736 |
| mmu-miR-7093-5p | -1.436924616 | 0.724728327 |
| mmu-miR-7093-3p | -1.000767277 | 0.510388409 |
| mmu-miR-7115-5p | -1.786311568 | 0.318170914 |
| mmu-miR-7115-3p | -1.806817692 | 0.477693277 |
| mmu-miR-7116-5p | -2.011456038 | 0.620417902 |
| mmu-miR-7116-3p | -0.389203325 | 0.869438706 |
| mmu-miR-7117-5p | -1.436924616 | 0.724728327 |
| mmu-miR-7118-5p | -1.436924616 | 0.724728327 |
| mmu-miR-7118-3p | -0.475143602 | 0.900417779 |
| mmu-miR-712-5p | 0.971429693 | 0.639781173 |
| mmu-miR-712-3p | -1.436924616 | 0.724728327 |
| mmu-miR-7211-3p | 1.364507625 | 0.719476388 |
| mmu-miR-7213-5p | 1.742061131 | 0.402799524 |
| mmu-miR-7214-5p | -1.436924616 | 0.724728327 |
| mmu-miR-7220-5p | -0.289761387 | 0.71415737 |
| mmu-miR-7220-3p | 0.855397695 | 0.594589732 |
| mmu-miR-7224-5p | -0.475143225 | 0.890451144 |
| mmu-miR-7224-3p | 0.782975192 | 0.079059845 |
| mmu-miR-7226-3p | -0.735347584 | 0.561447011 |
| mmu-miR-7227-3p | -1.436924616 | 0.724728327 |
| mmu-miR-7231-5p | -0.639891266 | 0.798042762 |
| mmu-miR-7235-3p | 0.114219005 | 0.914689033 |
| mmu-miR-7236-5p | 0.486628736 | 0.905071015 |
| mmu-miR-7236-3p | -0.358824608 | 0.56032504 |
| mmu-miR-7237-3p | 0.700629623 | 0.747139052 |
| mmu-miR-7240-5p | 0.269539105 | 0.743585836 |
| mmu-miR-7240-3p | -0.112847787 | 0.936339881 |
| mmu-miR-7243-3p | -1.251672407 | 0.361132357 |
| mmu-miR-741-3p | 0.538541891 | 0.463387534 |
| mmu-miR-743a-3p | 1.364507625 | 0.719476388 |
| mmu-miR-743b-3p | -2.757785938 | 0.360996028 |
| mmu-miR-744-5p | 0.249108463 | 0.334028647 |
| mmu-miR-744-3p | 0.43003612 | 0.049906722 |
| mmu-miR-758-5p | 0.247208517 | 0.901843117 |
| mmu-miR-758-3p | -0.505756523 | 0.026250599 |
| mmu-miR-760-5p | -0.703598889 | 0.567086819 |
| mmu-miR-760-3p | -0.165496801 | 0.447270186 |
| mmu-miR-764-5p | 0.302380666 | 0.64199826 |
| mmu-miR-764-3p | 0.353408913 | 0.390513704 |
| mmu-miR-7646-5p | 1.428940174 | 0.516153121 |
| mmu-miR-7649-3p | 1.419943146 | 0.724874287 |
| mmu-miR-7651-5p | -1.020277511 | 0.385033113 |
| mmu-miR-7655-5p | -1.436924616 | 0.724728327 |
| mmu-miR-7655-3p | -1.436924616 | 0.724728327 |
| mmu-miR-7656-5p | -0.199210579 | 0.909767758 |
| mmu-miR-7656-3p | 0.486628736 | 0.905071015 |
| mmu-miR-7658-3p | -1.436924616 | 0.724728327 |
| mmu-miR-7659-5p | 0.447413054 | 0.652282968 |
| mmu-miR-7661-3p | 1.400370847 | 0.565622455 |
| mmu-miR-7662-3p | 1.364507625 | 0.719476388 |
| mmu-miR-7663-5p | 0.486628736 | 0.905071015 |
| mmu-miR-7663-3p | 0.486628736 | 0.905071015 |
| mmu-miR-7664-5p | 0.590901282 | 0.76142923 |
| mmu-miR-7664-3p | 0.404136953 | 0.713075915 |
| mmu-miR-7665-5p | -2.275533165 | 0.504726832 |
| mmu-miR-7666-3p | -1.436924616 | 0.724728327 |
| mmu-miR-7667-5p | -2.516040207 | 0.498035283 |
| mmu-miR-7667-3p | 0.438583487 | 0.874929839 |
| mmu-miR-7668-5p | -0.359123113 | 0.861080031 |
| mmu-miR-7668-3p | -0.475143622 | 0.900866421 |
| mmu-miR-7669-5p | -0.480334596 | 0.757194114 |
| mmu-miR-767 | -2.741204576 | 0.366128791 |
| mmu-miR-7671-5p | -0.475143652 | 0.901530955 |
| mmu-miR-7674-5p | 0.486628736 | 0.905071015 |
| mmu-miR-7674-3p | -1.066020245 | 0.747832307 |
| mmu-miR-7675-3p | -2.275533165 | 0.504726832 |
| mmu-miR-7679-5p | 0.486628736 | 0.905071015 |
| mmu-miR-7682-5p | -1.806936996 | 0.476985549 |
| mmu-miR-7682-3p | -1.436924616 | 0.724728327 |
| mmu-miR-7685-5p | 0.438583487 | 0.874929839 |
| mmu-miR-7685-3p | -1.295956452 | 0.642095461 |
| mmu-miR-7687-5p | -1.436924616 | 0.724728327 |
| mmu-miR-7687-3p | 0.142632724 | 0.94851098 |
| mmu-miR-7688-5p | 0.388306762 | 0.688999246 |
| mmu-miR-7689-3p | -1.0074095 | 0.366077834 |
| mmu-miR-770-5p | -0.070542147 | 0.836188448 |
| mmu-miR-770-3p | 0.266882024 | 0.376464873 |
| mmu-miR-7a-5p | -0.429818832 | 0.035831789 |
| mmu-miR-7a-1-3p | -0.032803272 | 0.869823803 |
| mmu-miR-7a-2-3p | 0.016464104 | 0.933384274 |
| mmu-novel-305 | 0.014369692 | 0.941881138 |
| mmu-miR-7b-5p | -0.312858649 | 0.127679254 |
| mmu-miR-7b-3p | 0.096033829 | 0.825951064 |
| mmu-miR-802-5p | 1.533793661 | 0.210028914 |
| mmu-miR-802-3p | 0.486628736 | 0.905071015 |
| mmu-miR-8097 | 0.486628736 | 0.905071015 |
| mmu-miR-8103 | 0.428476875 | 0.670652539 |
| mmu-miR-8106 | 1.923316019 | 0.617646858 |
| mmu-miR-8107 | -1.241369868 | 0.557479135 |
| mmu-miR-8111 | -0.299446278 | 0.775506242 |
| mmu-miR-8112 | 0.171053909 | 0.777401599 |
| mmu-miR-8113 | -1.436924616 | 0.724728327 |
| mmu-miR-8114 | -1.436924616 | 0.724728327 |
| mmu-miR-8115 | 2.703842326 | 0.256878747 |
| mmu-miR-8118 | 2.391684183 | 0.354273348 |
| mmu-miR-8120 | 0.222933447 | 0.82625959 |
| mmu-miR-871-3p | -0.039717827 | 0.97636703 |
| mmu-miR-872-5p | 0.214486436 | 0.233342418 |
| mmu-miR-872-3p | -0.01920449 | 0.947184381 |
| mmu-miR-873a-5p | 0.132261568 | 0.540716393 |
| mmu-miR-873a-3p | -0.162074717 | 0.491464378 |
| mmu-miR-874-5p | -0.133889395 | 0.666633653 |
| mmu-miR-874-3p | -0.316732112 | 0.109151289 |
| mmu-miR-876-5p | -0.13728868 | 0.71090367 |
| mmu-miR-877-5p | -0.367174358 | 0.249492474 |
| mmu-miR-877-3p | 0.031787729 | 0.907527546 |
| mmu-miR-878-5p | 0.486628736 | 0.905071015 |
| mmu-miR-878-3p | 0.486628736 | 0.905071015 |
| mmu-miR-879-5p | 0.291815791 | 0.413850823 |
| mmu-miR-879-3p | 0.293124842 | 0.483743215 |
| mmu-miR-880-3p | -2.157306429 | 0.368957753 |
| mmu-miR-881-3p | -3.35817888 | 0.08802375 |
| mmu-miR-9-5p | 0.046066655 | 0.857395422 |
| mmu-miR-9-3p | -0.366990254 | 0.039503656 |
| mmu-miR-92a-3p | -0.099496671 | 0.541175989 |
| mmu-miR-92a-1-5p | 1.012042476 | 0.588676388 |
| mmu-miR-92b-5p | -0.041365306 | 0.908037996 |
| mmu-miR-92b-3p | 0.093680713 | 0.620442057 |
| mmu-miR-93-5p | 0.041461449 | 0.838540582 |
| mmu-miR-93-3p | 0.076240006 | 0.748321354 |
| mmu-miR-935 | -1.218999109 | 0.499464862 |
| mmu-miR-96-5p | 5.392313827 | 1 |
| mmu-miR-96-3p | 4.546817851 | 1 |
| mmu-miR-9769-3p | 1.906167817 | 0.247803816 |
| mmu-miR-98-5p | -0.174941302 | 0.401599088 |
| mmu-miR-98-3p | -0.557206792 | 0.07971676 |
| mmu-miR-99a-5p | -0.092049593 | 0.700897661 |
| mmu-miR-99a-3p | -0.13453674 | 0.605308464 |
| mmu-miR-99b-5p | 0.000970813 | 0.9971484 |
| mmu-miR-99b-3p | 0.34340189 | 0.158884051 |

**Supplementary Table 4 Overlapped miRNAs in predicted and sequenced miRNAs**

| **circRNAs** | **miRNAs** |
| --- | --- |
| circRNA03556 | mmu-miR-1903, mmu-miR-12183-5p, mmu-miR-761, mmu-miR-7231-3p, mmu-miR-207, mmu-miR-384-3p, mmu-miR-7054-3p, mmu-miR-7032-5p, mmu-miR-686, mmu-miR-6961-3p, mmu-miR-19b-2-5p, mmu-miR-8097, mmu-miR-6925-5p, mmu-miR-877-3p |
| circRNA03725 | mmu-miR-7094-1-5p, mmu-miR-181b-5p, mmu-miR-181d-5p, mmu-miR-9768-3p, mmu-miR-6970-5p, mmu-miR-12205-5p, mmu-miR-6414, mmu-miR-3068-5p, mmu-miR-7116-3p, mmu-miR-7b-5p, mmu-miR-146b-5p, mmu-miR-669g, mmu-miR-34b-3p, mmu-miR-141-5p, mmu-miR-1904 |
| circRNA01979 | mmu-miR-107-5p, mmu-miR-6946-3p, mmu-miR-1903, mmu-miR-103-1-5p, mmu-miR-103-2-5p, mmu-miR-6999-3p, mmu-miR-3099-5p, mmu-miR-320-5p, mmu-miR-6344, mmu-miR-7231-3p, mmu-miR-12183-5p, mmu-miR-3098-3p, mmu-miR-7116-3p, mmu-miR-6932-3p, mmu-miR-7092-3p, mmu-miR-6935-3p, mmu-miR-693-3p, mmu-miR-207, mmu-miR-7b-5p, mmu-miR-7028-3p |
| circRNA00533 | mmu-miR-7680-5p, mmu-miR-30d-3p, mmu-miR-30e-3p, mmu-miR-34b-3p, mmu-miR-5619-5p, mmu-miR-290b-3p, mmu-miR-6963-5p, mmu-miR-7013-5p, mmu-miR-146b-5p, mmu-miR-6951-5p, mmu-miR-6901-3p, mmu-miR-761, mmu-miR-145b, mmu-miR-7659-5p, mmu-miR-7663-5p, mmu-miR-145a-5p, mmu-miR-146a-5p |
| circRNA01891 | mmu-miR-6946-3p, mmu-miR-145a-3p, mmu-miR-7116-3p, mmu-miR-7092-3p, mmu-miR-107-5p, mmu-miR-26a-2-3p, mmu-miR-103-1-5p, mmu-miR-103-2-5p, mmu-miR-691, mmu-miR-7231-3p, mmu-miR-130a-5p, mmu-miR-6932-3p, mmu-miR-3081-5p, mmu-miR-6951-3p, mmu-miR-6967-3p, mmu-miR-6935-3p, mmu-miR-3074-5p, mmu-miR-7008-3p, mmu-miR-710, mmu-miR-12202-3p, mmu-miR-3068-5p, mmu-miR-7084-3p |
| circRNA03723 | mmu-miR-466b-5p, mmu-miR-466o-5p, mmu-miR-7661-5p, mmu-miR-466i-5p, mmu-miR-6970-5p, mmu-miR-3112-3p, mmu-miR-3068-5p, mmu-miR-297c-5p, mmu-miR-1187, mmu-miR-297a-5p, mmu-miR-6984-3p, mmu-miR-6414, mmu-let-7f-5p, mmu-miR-466c-5p, mmu-miR-466l-5p, mmu-miR-466a-5p, mmu-miR-466e-5p, mmu-miR-6925-5p, mmu-miR-7094-1-5p |
| circRNA04809 | mmu-miR-6998-3p, mmu-miR-667-5p, mmu-miR-320-5p, mmu-miR-7030-5p, mmu-miR-6921-5p, mmu-miR-6899-3p, mmu-miR-7688-3p, mmu-miR-6948-3p, mmu-miR-6958-3p, mmu-miR-23b-5p, mmu-miR-1903, mmu-miR-6919-3p, mmu-miR-7652-3p, mmu-miR-7663-5p, mmu-miR-12183-5p, mmu-miR-7226-5p |
| circRNA00343 | mmu-miR-3081-5p, mmu-miR-669d-5p, mmu-miR-8097, mmu-miR-290b-3p, mmu-miR-216b-3p, mmu-miR-7031-5p, mmu-miR-6393, mmu-miR-7683-3p, mmu-miR-297a-5p, mmu-miR-297c-5p, mmu-miR-742-5p, mmu-miR-7027-5p, mmu-miR-9768-5p, mmu-miR-7010-5p, mmu-miR-298-3p, mmu-miR-873a-5p |
| circRNA03712 | mmu-miR-153-5p, mmu-miR-466q, mmu-miR-27b-5p, mmu-miR-1904, mmu-miR-6951-5p, mmu-miR-3095-5p, mmu-miR-30a-3p, mmu-miR-30e-3p, mmu-miR-7680-5p, mmu-miR-700-5p, mmu-miR-693-3p, mmu-miR-7013-3p, mmu-miR-12205-5p, mmu-miR-145b, mmu-miR-6963-5p, mmu-miR-384-3p, mmu-miR-6999-3p |
| circRNA01543 | mmu-miR-7649-3p, mmu-miR-6961-3p, mmu-miR-204-5p, mmu-miR-211-5p, mmu-miR-7116-3p, mmu-miR-3473c, mmu-miR-9768-5p, mmu-miR-1947-5p, mmu-miR-691 |
| circRNA03390 | mmu-miR-669g, mmu-miR-7033-5p, mmu-miR-7013-3p, mmu-miR-3095-3p, mmu-miR-743a-5p, mmu-miR-6987-5p, mmu-miR-6900-5p, mmu-miR-709, mmu-miR-5619-5p, mmu-miR-26b-5p, mmu-miR-871-5p, mmu-miR-7032-5p, mmu-miR-7218-5p |
| circRNA03847 | mmu-miR-693-3p, mmu-miR-743a-5p, mmu-miR-6405, mmu-miR-871-5p, mmu-miR-141-5p, mmu-miR-7018-5p, mmu-miR-450a-1-3p, mmu-miR-1947-5p |
| circRNA00982 | mmu-miR-6951-5p, mmu-miR-7214-5p, mmu-miR-489-5p, mmu-miR-6951-3p, mmu-miR-6344, mmu-miR-883b-5p, mmu-miR-103-1-5p, mmu-miR-103-2-5p, mmu-miR-107-5p, mmu-miR-6899-3p, mmu-miR-26b-5p, mmu-miR-7116-3p, mmu-miR-7010-5p, mmu-miR-1958, mmu-miR-8092 |
| circRNA02067 | mmu-miR-6900-5p, mmu-miR-677-3p, mmu-miR-1291, mmu-miR-8094, mmu-miR-712-5p, mmu-miR-6987-5p, mmu-miR-1843a-5p, mmu-miR-7088-5p, mmu-miR-3095-3p, mmu-miR-5623-5p, mmu-miR-6958-3p, mmu-miR-7227-5p, mmu-miR-669e-5p, mmu-miR-145a-3p |
| circRNA01985 | mmu-miR-12202-3p, mmu-miR-320-5p, mmu-miR-6946-3p, mmu-miR-7028-3p, mmu-miR-7030-3p, mmu-miR-298-3p, mmu-miR-9768-3p, mmu-miR-6901-3p, mmu-miR-7031-5p, mmu-miR-873a-5p, mmu-miR-7657-3p, mmu-miR-134-5p, mmu-miR-7030-5p |
| circRNA00825 | mmu-miR-877-3p, mmu-miR-7012-5p, mmu-miR-7030-5p, mmu-miR-7226-5p, mmu-miR-504-3p, mmu-miR-6418-5p, mmu-miR-7058-5p, mmu-miR-188-3p, mmu-miR-130a-5p, mmu-miR-1904, mmu-miR-501-5p, mmu-miR-7018-5p, mmu-miR-5114, mmu-miR-7050-5p, mmu-miR-3473a, mmu-miR-6539, mmu-miR-330-5p, mmu-miR-6403, mmu-miR-669c-3p |
| circRNA03309 | mmu-miR-7050-5p, mmu-miR-185-3p, mmu-miR-7012-5p, mmu-miR-3095-5p, mmu-miR-7084-3p, mmu-miR-6418-5p, mmu-miR-6931-5p, mmu-miR-7026-3p, mmu-miR-6907-5p, mmu-miR-106a-3p, mmu-miR-700-5p |
| circRNA02418 | mmu-miR-7092-3p, mmu-miR-7678-3p, mmu-miR-130a-5p, mmu-miR-6935-3p, mmu-miR-7116-3p, mmu-miR-6344, mmu-miR-12205-5p, mmu-miR-6984-3p, mmu-miR-6999-3p, mmu-miR-26a-2-3p, mmu-miR-203-5p, mmu-miR-6964-3p, mmu-miR-7657-3p, mmu-miR-23b-5p, mmu-miR-5101, mmu-miR-7231-3p, mmu-miR-7663-5p, mmu-miR-691, mmu-miR-6928-3p, mmu-miR-3099-5p |
| circRNA04488 | mmu-miR-667-5p, mmu-miR-30c-1-3p, mmu-miR-5623-5p, mmu-miR-1843a-5p, mmu-miR-5110, mmu-miR-7649-3p, mmu-miR-7092-3p, mmu-miR-5114 |
| circRNA01605 | mmu-miR-7218-5p, mmu-miR-7652-3p, mmu-miR-146b-5p, mmu-miR-106a-3p, mmu-miR-146a-5p, mmu-miR-20b-3p, mmu-miR-185-5p, mmu-miR-7222-5p, mmu-miR-12202-3p, mmu-miR-3110-3p |
| circRNA03073 | mmu-miR-7058-5p, mmu-miR-672-3p, mmu-miR-7033-5p, mmu-miR-7226-5p, mmu-miR-669c-3p, mmu-miR-185-5p, mmu-miR-7661-5p, mmu-miR-466f-3p, mmu-miR-6373, mmu-miR-7094-1-5p, mmu-miR-6418-5p, mmu-miR-504-3p, mmu-miR-3473a, mmu-miR-6925-5p, mmu-miR-7222-5p, mmu-miR-188-3p, mmu-miR-5110, mmu-miR-466q, mmu-miR-145a-5p, mmu-miR-3097-5p |
| circRNA02313 | mmu-miR-7652-3p, mmu-miR-7649-3p, mmu-miR-710, mmu-miR-181b-5p, mmu-miR-1903, mmu-miR-3473c, mmu-miR-6931-5p, mmu-miR-8092, mmu-miR-181d-5p, mmu-miR-7688-3p, mmu-miR-666-3p, mmu-miR-7238-3p, mmu-miR-6965-3p |
| circRNA00723 | mmu-miR-466i-5p, mmu-miR-5623-5p, mmu-miR-7058-5p, mmu-miR-669d-5p, mmu-miR-466l-5p, mmu-miR-709, mmu-miR-669f-5p, mmu-miR-6921-5p, mmu-miR-669a-5p, mmu-miR-1187, mmu-miR-669p-5p, mmu-miR-466a-5p, mmu-miR-466e-5p |
| circRNA00447 | mmu-miR-6948-3p, mmu-miR-7092-3p, mmu-miR-8094, mmu-miR-134-5p, mmu-miR-667-5p, mmu-miR-712-5p, mmu-miR-30c-1-3p, mmu-miR-27b-5p, mmu-miR-669h-5p, mmu-miR-8108, mmu-miR-6373, mmu-miR-181b-2-3p |
| circRNA03673 | mmu-miR-1952, mmu-miR-421-3p, mmu-miR-7655-5p, mmu-miR-185-3p, mmu-miR-883b-5p, mmu-miR-494-5p, mmu-miR-3071-3p, mmu-miR-3062-5p, mmu-miR-7658-3p, mmu-miR-6945-5p, mmu-miR-7077-5p, mmu-miR-667-5p, mmu-miR-7686-5p, mmu-miR-7222-5p, mmu-miR-124b-3p, mmu-miR-3110-3p, mmu-miR-3099-5p, mmu-miR-1188-5p, mmu-miR-6403, mmu-miR-3971, mmu-miR-669h-5p |
| circRNA04655 | mmu-miR-669a-5p, mmu-miR-669p-5p, mmu-miR-12205-5p, mmu-miR-8108, mmu-miR-7054-5p, mmu-miR-693-5p, mmu-miR-669f-5p, mmu-miR-6393, mmu-miR-743a-5p, mmu-miR-672-3p, mmu-miR-3098-3p, mmu-miR-3095-5p, mmu-miR-7021-3p, mmu-let-7f-5p |
| circRNA00747 | mmu-miR-1958, mmu-miR-7092-3p, mmu-miR-6967-3p, mmu-miR-19b-2-5p, mmu-miR-211-5p, mmu-miR-6951-5p, mmu-miR-7012-5p, mmu-miR-6964-3p, mmu-miR-26a-2-3p, mmu-miR-153-5p, mmu-miR-7661-5p, mmu-miR-677-3p, mmu-miR-204-5p, mmu-miR-7651-3p, mmu-miR-5619-5p, mmu-miR-686, mmu-miR-6899-3p |
| circRNA03674 | mmu-miR-9768-3p, mmu-miR-7077-5p, mmu-miR-7027-5p, mmu-miR-1952, mmu-miR-421-3p, mmu-miR-6934-5p, mmu-miR-7226-5p, mmu-miR-124b-3p, mmu-miR-7655-5p, mmu-miR-185-3p, mmu-miR-883b-5p, mmu-miR-7010-5p, mmu-miR-494-5p, mmu-miR-1188-5p, mmu-miR-3071-3p, mmu-miR-3062-5p, mmu-miR-7054-5p, mmu-miR-7658-3p, mmu-miR-7683-3p, mmu-miR-6945-5p, mmu-miR-667-5p, mmu-miR-7686-5p, mmu-miR-6901-3p, mmu-miR-188-3p |
| circRNA03831 | mmu-miR-7092-3p, mmu-miR-7651-3p, mmu-miR-30d-3p, mmu-miR-30e-3p, mmu-miR-30a-3p, mmu-miR-17-3p, mmu-miR-6899-3p, mmu-miR-6935-3p, mmu-miR-106a-3p, mmu-miR-7238-3p, mmu-miR-20b-3p, mmu-miR-666-3p, mmu-miR-6999-3p, mmu-miR-760-3p, mmu-miR-742-5p, mmu-miR-7214-5p, mmu-miR-6928-3p, mmu-miR-3112-3p |
| circRNA06452 | mmu-miR-3473c, mmu-miR-7013-5p, mmu-miR-7649-3p, mmu-miR-6539, mmu-miR-12187-3p, mmu-miR-691, mmu-miR-130a-5p, mmu-miR-669e-5p, mmu-miR-6946-3p, mmu-miR-693-5p, mmu-miR-7663-5p, mmu-miR-6945-5p, mmu-miR-3971, mmu-miR-7063-3p, mmu-miR-6919-3p |
| circRNA08673 | mmu-miR-7021-3p, mmu-miR-7030-3p, mmu-miR-7008-3p, mmu-miR-6965-3p, mmu-miR-421-3p, mmu-miR-9768-3p, mmu-miR-7063-3p, mmu-miR-7077-5p, mmu-miR-7092-3p, mmu-miR-207, mmu-miR-7027-5p, mmu-miR-1952, mmu-miR-6934-5p, mmu-miR-7226-5p, mmu-miR-124b-3p, mmu-miR-7686-5p, mmu-miR-7655-5p, mmu-miR-185-3p |
| circRNA06661 | mmu-miR-335-3p, mmu-miR-7227-5p, mmu-miR-6539, mmu-miR-6907-5p, mmu-miR-12187-3p, mmu-miR-450a-1-3p, mmu-miR-7b-5p, mmu-miR-466f-3p, mmu-miR-216b-3p, mmu-miR-6405, mmu-miR-330-5p |
| circRNA08440 | mmu-miR-667-5p, mmu-miR-17-3p, mmu-miR-335-3p, mmu-miR-3097-5p, mmu-miR-203-5p, mmu-miR-5101, mmu-miR-466b-5p, mmu-miR-466o-5p, mmu-miR-7088-5p, mmu-miR-3971, mmu-miR-7649-3p, mmu-miR-666-3p, mmu-miR-466c-5p |
| circRNA09919 | mmu-miR-7054-3p, mmu-miR-7026-3p, mmu-miR-6958-3p, mmu-miR-3074-5p, mmu-miR-181b-2-3p, mmu-miR-501-5p, mmu-miR-1188-5p, mmu-miR-30d-3p, mmu-miR-6998-3p, mmu-miR-489-5p, mmu-miR-7226-5p, mmu-miR-760-3p, mmu-miR-7678-3p, mmu-miR-30a-3p, mmu-miR-7659-5p, mmu-miR-1291 |

**Supplementary Table 5 Key miRNAs in the circRNA-miRNA network**

| **miRNAs** | **Degree** | **Betweenness Centrality** | **Closeness Centrality** | **Neighborhood Connectivity** |
| --- | --- | --- | --- | --- |
| mmu-miR-7092-3p | 8 | 0.095627 | 0.288509 | 18.375 |
| mmu-miR-7116-3p | 6 | 0.062243 | 0.270332 | 18.5 |
| mmu-miR-667-5p | 6 | 0.055892 | 0.263982 | 18 |
| mmu-miR-7649-3p | 5 | 0.059372 | 0.250265 | 17.6 |
| mmu-miR-7663-5p | 4 | 0.027006 | 0.258206 | 19.25 |
| mmu-miR-6999-3p | 4 | 0.020656 | 0.264871 | 21 |
| mmu-miR-6951-5p | 4 | 0.025265 | 0.252677 | 20.75 |
| mmu-miR-6946-3p | 4 | 0.028238 | 0.252677 | 17 |
| mmu-miR-6935-3p | 4 | 0.009519 | 0.253763 | 20 |
| mmu-miR-6899-3p | 4 | 0.023295 | 0.256522 | 19.75 |
| mmu-miR-185-3p | 4 | 0.020768 | 0.231827 | 17.25 |
| mmu-miR-130a-5p | 4 | 0.023003 | 0.251331 | 18.5 |
| mmu-miR-7b-5p | 3 | 0.025793 | 0.245067 | 18 |
| mmu-miR-7655-5p | 3 | 0.002633 | 0.225621 | 18 |
| mmu-miR-7077-5p | 3 | 0.002633 | 0.225621 | 18 |
| mmu-miR-7027-5p | 3 | 0.015146 | 0.239837 | 18 |
| mmu-miR-7012-5p | 3 | 0.024048 | 0.234359 | 16.33333 |
| mmu-miR-7010-5p | 3 | 0.026849 | 0.252677 | 19.33333 |
| mmu-miR-6958-3p | 3 | 0.01376 | 0.227141 | 19.66667 |
| mmu-miR-693-3p | 3 | 0.040518 | 0.240081 | 18.66667 |
| mmu-miR-6925-5p | 3 | 0.02363 | 0.224548 | 18 |
| mmu-miR-6901-3p | 3 | 0.025195 | 0.241556 | 18 |
| mmu-miR-666-3p | 3 | 0.021729 | 0.238384 | 20 |
| mmu-miR-6539 | 3 | 0.022584 | 0.237903 | 18.66667 |
| mmu-miR-5623-5p | 3 | 0.027739 | 0.231373 | 18.66667 |
| mmu-miR-5619-5p | 3 | 0.025055 | 0.239594 | 18.66667 |
| mmu-miR-421-3p | 3 | 0.002633 | 0.225621 | 18 |
| mmu-miR-3971 | 3 | 0.012772 | 0.234592 | 20 |
| mmu-miR-30e-3p | 3 | 0.012771 | 0.25 | 23.33333 |
| mmu-miR-30d-3p | 3 | 0.01329 | 0.246347 | 23.33333 |
| mmu-miR-30a-3p | 3 | 0.018969 | 0.249471 | 23.66667 |
| mmu-miR-3099-5p | 3 | 0.012884 | 0.243802 | 18.66667 |
| mmu-miR-3095-5p | 3 | 0.026206 | 0.230469 | 19.33333 |
| mmu-miR-3068-5p | 3 | 0.022474 | 0.254037 | 20 |
| mmu-miR-26a-2-3p | 3 | 0.004542 | 0.23671 | 19 |
| mmu-miR-188-3p | 3 | 0.014601 | 0.239351 | 16.33333 |
| mmu-miR-146b-5p | 3 | 0.030709 | 0.23506 | 19.33333 |
| mmu-miR-1188-5p | 3 | 0.014147 | 0.234359 | 20.33333 |
| mmu-miR-107-5p | 3 | 0.004706 | 0.245833 | 18.33333 |
| mmu-miR-103-2-5p | 3 | 0.004706 | 0.245833 | 18.33333 |
| mmu-miR-103-1-5p | 3 | 0.004706 | 0.245833 | 18.33333 |

**Supplementary Table 6 The circRNA-related gene in KEGG pathway analysis**

|  | Term | Description | LogP | Log(q-value) | Symbols |
| --- | --- | --- | --- | --- | --- |
| Upreguated circRNAs-related KEGG pathway | mmu04360 | Axon guidance | -15.7769 | -13.247 | Bmpr2,Camk2b,Camk2g,Dcc,Efna3,Efna5,Efnb3,Enah,Epha3,Epha4,Epha5,Epha6,Epha7,Epha8,Ephb3,Ephb6,Fyn,Fzd3,Gnai3,Itgb1,L1cam,Limk2,Nfatc2,Nfatc3,Nras,Nrp1,Pak3,Pik3r3,Plxna1,Plxna2,Ppp3ca,Ppp3r1,Robo1,Cxcl12,Sema3a,Sema4b,Sema4c,Sema4f,Sema5a,Sema6b,Slit2,Src,Wnt4,Mapk1,Sema4g,Arhgef12,Pik3cb,Ntng1,Unc5a,Camk2d,Srgap1,Lrrc4,Ablim2,Plxna4,Rgma,Srgap3,Lrig2 |
|  | mmu04010 | MAPK signaling pathway | -10.4315 | -8.2541 | Angpt1,Atf2,Cacna1b,Cacna1e,Cacnb2,Cacnb4,Casp3,Crk,Crkl,Csf1,Efna3,Efna5,Elk4,Erbb4,Mecom,Fasl,Fgf1,Fgf4,Hgf,Igf1,Igf1r,Insr,Max,Mef2c,Kitl,Nfatc3,Ngf,Nlk,Nras,Ntrk2,Pdgfb,Pdgfra,Pdgfrb,Ppp3ca,Ppp3r1,Ppp5c,Rasgrp1,Rps6ka1,Sos1,Tgfb3,Tgfbr1,Traf6,Akt3,Map3k1,Map3k2,Map3k3,Mapk1,Mapk14,Mapk8,Cacna1h,Fgf23,Map3k20,Rras2,Dusp6,Tab2,Dusp16,Map3k13,Rps6ka5,Arrb1,Braf,Rap1a,Rps6ka3,Rasa2,Rap1b,Taok1,Pla2g4e |
|  | mmu04068 | FoxO signaling pathway | -8.98069 | -7.0526 | Bcl2l11,Ccnd2,Cdkn1b,S1pr1,Fasl,Grm1,Foxg1,Igf1,Igf1r,Il10,Insr,Smad3,Smad4,Nlk,Nras,Pik3r3,Pten,Rbl2,Slc2a4,Sos1,Stat3,Tgfb3,Tgfbr1,Akt3,Mapk1,Mapk14,Mapk8,Homer1,Homer2,Foxo4,Foxo1,Fbxo32,Setd7,Pik3cb,Braf,Agap2,Ccnb1 |
|  | mmu04722 | Neurotrophin signaling pathway | -8.24577 | -6.4937 | Bcl2,Camk2b,Camk2g,Camk4,Crk,Crkl,Fasl,Sh2b3,Ngf,Nras,Ntrk2,Pik3r3,Prkcd,Rps6ka1,Shc1,Sort1,Sos1,Traf6,Akt3,Map3k1,Map3k3,Mapk1,Mapk14,Mapk8,Prdm4,Rps6ka5,Pik3cb,Camk2d,Braf,Rap1a,Rps6ka3,Rap1b,Shc4,Frs2 |
|  | mmu04014 | Ras signaling pathway | -7.89315 | -6.2081 | Abl2,Angpt1,Arf6,Csf1,Efna3,Efna5,Fasl,Fgf1,Fgf4,Gng4,Grin2b,Hgf,Igf1,Igf1r,Insr,Kitl,Ngf,Nras,Ntrk2,Pak3,Pdgfb,Pdgfra,Pdgfrb,Pik3r3,Rab5b,Rasa3,Rasal1,Rasgrp1,Ralgds,Rgl1,Shc1,Sos1,Tiam1,Akt3,Ets1,Ets2,Mapk1,Mapk8,Foxo4,Rassf1,Fgf23,Rras2,Plce1,Pik3cb,Rap1a,Rasa2,Rap1b,Rasal2,Pla2g3,Shc4,Pla2g4e |
|  | mmu04015 | Rap1 signaling pathway | -7.31533 | -5.6882 | Adcy6,Angpt1,Ctnnd1,Cnr1,Crk,Crkl,Csf1,Efna3,Efna5,Enah,Fgf1,Fgf4,Gnai3,Grin2b,Magi1,Hgf,Igf1,Igf1r,Insr,Itgb1,Kitl,Ngf,Nras,Pdgfb,Pdgfra,Pdgfrb,Pik3r3,Plcb1,Ralgds,Src,Tiam1,Akt3,Mapk1,Mapk14,Magi2,Vav3,Fgf23,Tln2,Plce1,Pik3cb,Prkd3,Braf,Rap1a,Rapgef6,Rap1b,Dock4,Sipa1l2 |
|  | mmu04140 | Autophagy - animal | -6.89037 | -5.3144 | Bcl2,Hif1a,Igf1r,Itpr1,Nras,Pik3r3,Prkcd,Pten,Rab1a,Rab7,Rheb,Traf6,Akt3,Mapk1,Mapk8,Ulk2,Rragd,Rras2,Snap29,Stx17,Trp53inp2,Rps6kb1,C9orf72,Mtmr3,Ddit4,Pik3cb,Atg2b,Atg16l1,Mtmr4,Zfyve1,Smcr8,Atg9a,Nrbf2,Rubcn,Atg14 |
|  | mmu04550 | Signaling pathways regulating pluripotency of stem cells | -6.05952 | -4.5375 | Acvr1,Acvr1b,Zfhx3,Bmpr2,Smarcad1,Fzd3,Fzd5,Hoxa1,Hoxb1,Igf1,Igf1r,Il6st,Smad2,Smad3,Smad4,Smad5,Nras,Pik3r3,Rest,Skil,Stat3,Tcf3,Wnt9b,Wnt4,Wnt8b,Akt3,Esrrb,Mapk1,Mapk14,Pcgf3,Pik3cb,Kat6a,Acvr1c |
|  | mmu04024 | cAMP signaling pathway | -5.70857 | -4.3825 | Adcy6,Adcyap1,Adrb1,Adrb2,Atp1b1,Atp1b2,Atp2b2,Camk2b,Camk2g,Camk4,Creb1,Edn1,Gnai3,Grin2b,Hhip,Htr1a,Htr4,Pde4a,Pde4b,Pik3r3,Ppara,Ppp1cc,Slc9a1,Tiam1,Akt3,Pde10a,Mapk1,Mapk8,Gria3,Gabbr1,Vav3,Rras2,Atp1b4,Plce1,Pik3cb,Atp1a2,Camk2d,Braf,Rap1a,Rap1b,Creb5,Pde4d,Abcc4,Atp2b4 |
|  | mmu04390 | Hippo signaling pathway | -4.10715 | -3.1838 | Bmpr2,Ccnd2,Dlg4,Fgf1,Fzd3,Fzd5,Lats1,Smad2,Smad3,Smad4,Serpine1,Ppp1cc,Tcf7l2,Tead1,Tgfb3,Tgfbr1,Wnt9b,Wnt4,Wnt8b,Ywhag,Ywhaz,Dlg2,Lats2,Rassf1,Sav1,Dlg5,Ppp2r2a,Rassf6,Nkd1,Bbc3,Gdf6 |
| Downreguated circRNAs-related KEGG pathway | mmu04360 | Axon guidance | -20.2065 | -17.676 | Rhoa,Bmpr2,Camk2b,Cfl2,Dpysl2,Dcc,Efna3,Efna5,Efnb1,Efnb3,Enah,Epha4,Epha5,Epha6,Epha7,Epha8,Ephb2,Ephb3,Ephb6,Fyn,Fzd3,Gnai1,Itgb1,Kras,L1cam,Limk2,Met,Nck2,Nfatc3,Nras,Nrp1,Pak1,Pak3,Pik3ca,Pik3r1,Pik3r3,Plxna2,Ppp3r1,Ppp3r2,Robo1,Rock2,Sema4b,Sema4c,Sema4f,Sema5a,Sema6a,Slit2,Src,Unc5c,Wnt4,Mapk1,Sema4g,Plxnc1,Gsk3b,Pik3cb,Ntng1,Camk2d,Plxnb2,Unc5d,Pak6,Ablim2,Ssh2,Pak5,Plxna4,Rgma,Srgap3,Lrig2 |
|  | mmu04140 | Autophagy - animal | -14.9129 | -12.769 | Bcl2,Bcl2l1,Rb1cc1,Ctsl,Hif1a,Igf1r,Itpr1,Kras,Rab8a,Mras,Nras,Pik3ca,Pik3r1,Pik3r3,Prkacb,Prkcd,Pten,Rab1a,Rab7,Rheb,Traf6,Ulk1,Akt3,Map2k1,Mapk1,Mapk10,Mapk8,Mapk9,Ulk2,Rragd,Gabarap,Mlst8,Gabarapl1,Tsc1,Rras2,Stx17,Trp53inp2,C9orf72,Ddit4,Pik3cb,Vmp1,Atg2b,Prkaa1,Prkaa2,Mtmr4,Zfyve1,Smcr8,Atg9a,Nrbf2,Rubcn,Atg14 |
|  | mmu04150 | mTOR signaling pathway | -8.99522 | -7.6111 | Rhoa,Atp6v1a,Atp6v1b2,Fzd3,Grb10,Igf1r,Insr,Kras,Nras,Pik3ca,Pik3r1,Pik3r3,Pten,Rheb,Rps6ka1,Sos1,Sos2,Ulk1,Wnt9b,Wnt2b,Wnt4,Wnt7a,Akt3,Map2k1,Mapk1,Eif4e2,Ulk2,Rragd,Clip1,Gsk3b,Mlst8,Tsc1,Seh1l,Ddit4,Pik3cb,Rictor,Prkaa1,Prkaa2,Braf,Rps6ka3,Fnip1,Mapkap1,Rnf152,Fnip2 |
|  | mmu04010 | MAPK signaling pathway | -14.8221 | -12.769 | Angpt1,Atf2,Cacna1b,Cacna1e,Cacnb2,Cacnb4,Crk,Crkl,Csf1,Dusp2,Efna3,Efna5,Elk4,Erbb4,Ereg,Mecom,Fgf1,Gng12,Hspa1b,Igf1r,Il1r1,Insr,Kdr,Kras,Met,Kitl,Mras,Mapt,Nfatc3,Nlk,Nras,Ntrk2,Pak1,Pdgfb,Pdgfra,Pdgfrb,Prkacb,Ppp3r1,Ppp3r2,Rasgrf2,Rasgrp1,Rps6ka1,Sos1,Sos2,Srf,Tgfa,Tgfb3,Tgfbr1,Traf6,Akt3,Mapk7,Map2k1,Map2k7,Map3k1,Map3k12,Map3k2,Map3k3,Mapk1,Mapk10,Mapk8,Mapk9,Map4k4,Mapk8ip3,Cacna1h,Stk4,Fgf23,Map3k20,Rras2,Dusp6,Tab2,Dusp16,Map3k13,Rps6ka5,Rapgef2,Arrb1,Braf,Rap1a,Rps6ka3,Rasa2,Taok1 |
|  | mmu04722 | Neurotrophin signaling pathway | -11.1794 | -9.6492 | Rhoa,Bcl2,Camk2b,Camk4,Crk,Crkl,Kras,Sh2b3,Nras,Ntrk2,Pik3ca,Pik3r1,Pik3r3,Prkcd,Rps6ka1,Shc1,Sos1,Sos2,Traf6,Akt3,Mapk7,Map2k1,Map2k7,Map3k1,Map3k3,Mapk1,Mapk10,Mapk8,Mapk9,Foxo3,Gsk3b,Prdm4,Rps6ka5,Pik3cb,Rapgef1,Camk2d,Braf,Rap1a,Rps6ka3,Shc4,Frs2 |
|  | mmu04725 | Cholinergic synapse | -7.98842 | -6.8384 | Chrnb2,Adcy6,Bcl2,Cacna1b,Camk2b,Camk4,Creb1,Fyn,Gnai1,Gnao1,Gnb1,Gnb4,Gng12,Gng4,Itpr1,Itpr2,Jak2,Kcnj3,Kcnq2,Kras,Nras,Pik3ca,Pik3r1,Pik3r3,Prkacb,Plcb1,Akt3,Map2k1,Mapk1,Pik3cb,Camk2d,Kcnq5,Creb5,Adcy1 |
|  | mmu04151 | PI3K-Akt signaling pathway | -8.40278 | -7.1863 | Angpt1,Atf2,Bcl2,Bcl2l1,Bcl2l11,Ccnd2,Ccnd3,Cdk6,Col4a3,Col4a4,Col6a2,Col6a3,Col1a2,Creb1,Csf1,Efna3,Efna5,Erbb4,Ereg,Fgf1,G6pc,Gnb1,Gnb4,Gng12,Gng4,Ibsp,Igf1r,Il6ra,Insr,Itga5,Itga6,Itgb1,Jak2,Kdr,Kras,Met,Kitl,Nras,Ntrk2,Osm,Pdgfb,Pdgfra,Pdgfrb,Pik3ca,Pik3r1,Pik3r3,Prlr,Pten,Rbl2,Rheb,Sos1,Sos2,Tgfa,Tnc,Ywhaq,Akt3,Map2k1,Mapk1,Eif4e2,Magi2,Foxo3,Gsk3b,Mlst8,Fgf23,Tsc1,Ppp2r2a,Ddit4,Pik3cb,Itga9,Prkaa1,Prkaa2,Itga1,Ppp2r5a,Creb5,Ppp2r3a,Phlpp2 |
|  | mmu04211 | Longevity regulating pathway | -7.55189 | -6.4367 | Adcy6,Atf2,Camk4,Rb1cc1,Creb1,Igf1r,Insr,Kras,Nras,Pik3ca,Pik3r1,Pik3r3,Prkacb,Ppargc1a,Rheb,Ulk1,Akt3,Eif4e2,Foxo1,Foxo3,Tsc1,Adipor2,Adipor1,Pik3cb,Sirt1,Prkaa1,Prkaa2,Creb5,Adcy1 |
|  | mmu04724 | Glutamatergic synapse | -5.25674 | -4.3986 | Adcy6,Dlg4,Gnai1,Gnao1,Gnb1,Gnb4,Gng12,Gng4,Gria2,Grik2,Grin2b,Grm1,Itpr1,Itpr2,Kcnj3,Prkacb,Plcb1,Ppp3r1,Ppp3r2,Slc1a2,Mapk1,Homer1,Homer2,Slc38a2,Slc17a7,Slc17a6,Dlgap1,Grin3a,Adcy1 |
|  | mmu04727 | GABAergic synapse | -4.35295 | -3.6423 | Adcy6,Cacna1b,Gabra3,Gabra6,Gabrb2,Gabrb3,Slc6a12,Gad2,Gnai1,Gnao1,Gnb1,Gnb4,Gng12,Gng4,Prkacb,Src,Gabarap,Gabarapl1,Slc38a2,Trak2,Slc6a1,Gabbr2,Adcy1 |

**Supplementary** **Table 7 Comparison of the circRNAs and miRNAs with GEO dataset**

| miRNAs | Present study | | Published dataset | | | |
| --- | --- | --- | --- | --- | --- | --- |
|  | P value | logFC | P value | logFC | GEO dataset | sample |
| circRNA00825  (mmu_circ_0000404) | 0.001808 | 1.140773 | 0.034506 | 1.50723 | GSE158995 | APP/PS1 mice  hippocampus |
| circRNA01891  (mmu_circRNA_000079) | 0.012664 | 19.93244 | 0.100637 | 0.196 | GSE190880 | Aged C57BL/6J mice |
| circRNA03073  (mmu_circRNA_003477) | 0.023002 | -1.325214 | 0.017244 | -0.29 | GSE190880 | Aged C57BL/6J mice |
| circRNA06452  (mmu_circRNA_000947) | 0.015893 | -19.42529 | 0.284910 | -0.122 | GSE190880 | Aged C57BL/6J mice |
| circRNA08440  (mmu_circRNA_015227) | 0.010326 | -20.39221 | 0.001158 | -0.426 | GSE190880 | Aged C57BL/6J mice |
| circRNA01985  (mmu_circ_0001026) | 0.003080 | 1.402530 | 0.049692 | 0.41146 | GSE132177 | APP/PS1 mice |
| mmu-miR-669f-5p | 0.044532 | 1.835861 | 4.97E-02 | 0.832444 | GSE129053 | Aβ_1-42_ induced AD model rats hippocampus |
| mmu-miR-6240 | 0.009093 | 1.683446 | 0.003318 | -0.41981 | GSE52023 | Tg6799 mice hippocampus |
| mmu-miR-1969 | 0.008142 | -2.56641 | 6.91E-02 | -0.1157 | GSE166393 | APP/PS1 mice hippocampus |
| mmu-miR-3963 | 0.031801 | 1.120086 | 8.14E-02 | -0.42756 | GSE166393 | APP/PS1 mice hippocampus |
| miR-490-5p | 0.018928 | 1.036711 | 0.34488 | 1.27475 | GSE48028 | Tg2576 mice  hippocampus |
| miR-490-5p | 0.018928 | 1.036711 | 0.260741 | -1.57035 | GSE48028 | APP/PS1 mice  hippocampus |
